# Supplementary material for: Bridging pico-to-nanonewtons with a ratiometric force probe for monitoring nanoscale polymer physics before damage
Source: Nat Commun. 2022 Jan 13;13:303. doi: 10.1038/s41467-022-27972-y (PMC8758707; doi:10.1038/s41467-022-27972-y)
Supplement: Supplementary file 1 — Supplementary Information [file 41467_2022_27972_MOESM1_ESM.pdf]

Supplementary Information for

Bridging Pico-to-Nanonewtons with a Ratiometric Force Probe for Monitoring Nanoscale Polymer Physics Before Damage

Ryota Kotani, Soichi Yokoyama, Shunpei Nobusue, Shigehiro Yamaguchi, Atsuhiro Osuka, Hiroshi Yabu*, Shohei Saito*

Correspondence to: saito.shohei.4c@kuchem.kyoto-u.ac.jp

This PDF file includes:

The legend of Supplementary Movie 1
Supplementary Text
Supplementary Figs. 1 to 68
Supplementary Tables 1 to 24

The Other Supplementary Data:

Supplementary Movie 1

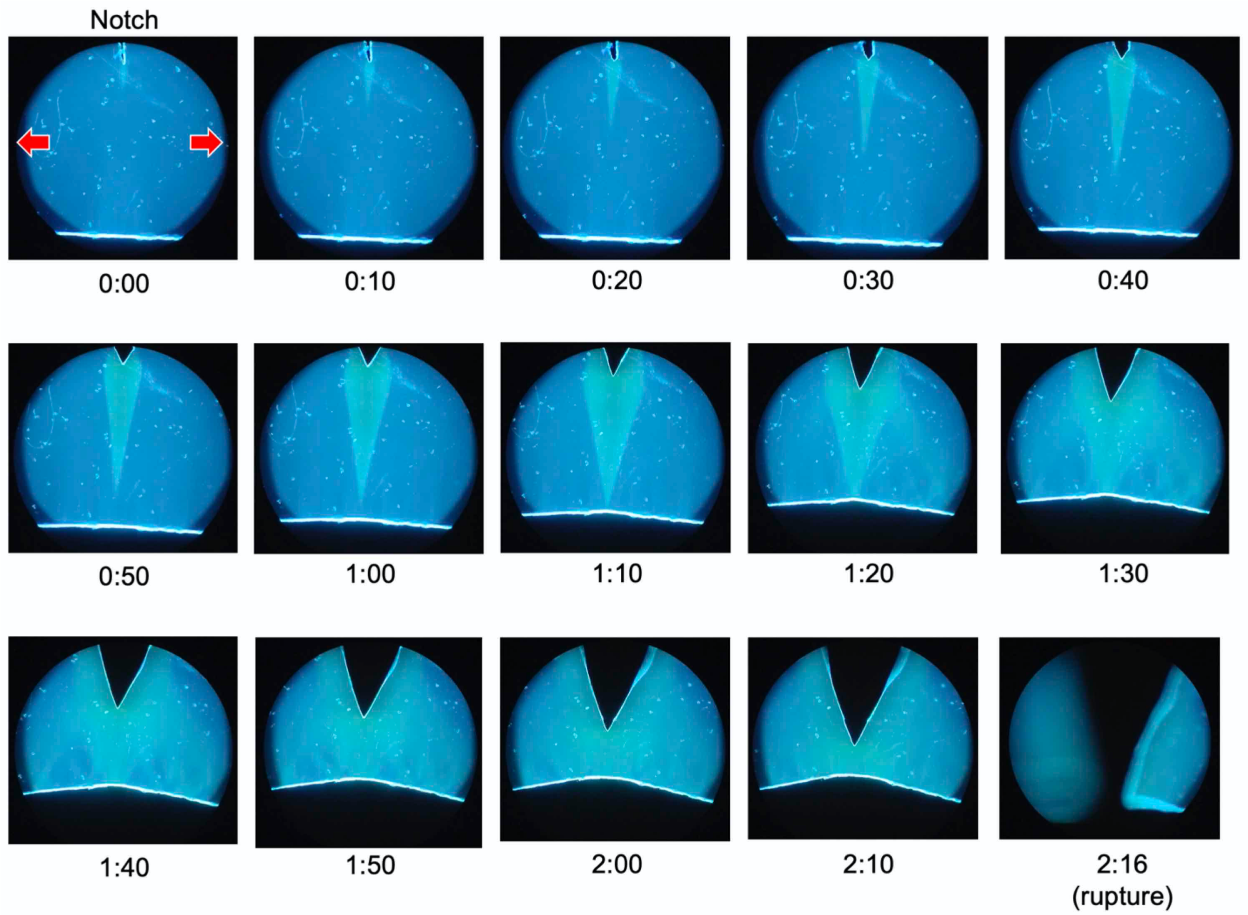

Supplementary Movie 1. Monitoring force transmission of the stretched polycarbonate film chemically doped with the dual fluorescent molecular force probe. Rapid growth of the stressed area was visualized much earlier than crack propagation, demonstrating a lower threshold for the fluorescence switch of the force probe, well below the force required for covalent bond scission. Excitation wavelength: 365 nm. The field of view of the microscope: 1 mm diameter. Stretching rate: 5 μm s^{-1}. Strain rate: 2×10^{-4} s^{-1}.

Synthesis of new compounds

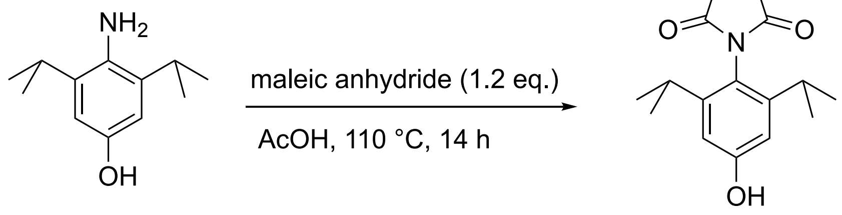

S1

_N_-(4-Hydroxy-2,6-diisopropylphenyl)maleimide (S1)

Under Ar atmosphere, 4-amino-3,5-diisopropylphenol[1] (2.77 g, 14.3 mmol) and maleic anhydride (1.68 g, 17.2 mmol) were added to a round-bottom flask (500 mL). After addition of AcOH (70 mL), the reaction mixture was stirred at 110 °C for 14 h. The resulting mixture diluted with EtOAc was poured into a separatory funnel, washed with saturated aqueous $NaHCO_3$ (3 times) and brine, dried over anhydrous Na_2SO_4 and evaporated. The crude product was purified by silica gel column chromatography (eluent: CH_2Cl_2/EtOAc = 20/1 by volume). Recrystallization from *n*-hexane furnished **S1** as a white solid (3.08 g, 79%).

^{1}H NMR (600 MHz, $CDCl_3$) δ (ppm) 6.87 (s, 2H), 6.69 (s, 2H), 4.80 (s, 1H), 2.56 (sept, J = 6.9 Hz, 2H) and 1.13 (d, J = 6.9 Hz, 12H); ^{13}C NMR (151 MHz, $CDCl_3$) δ (ppm) 170.99, 157.10, 149.54, 134.43, 118.95, 111.26, 29.50 and 23.95; HR-APCI TOF-MS (*m*/*z*) found 273.1358, calcd for $C_{16}H_{19}NO_3$: 273.1365 $[M]^+$.

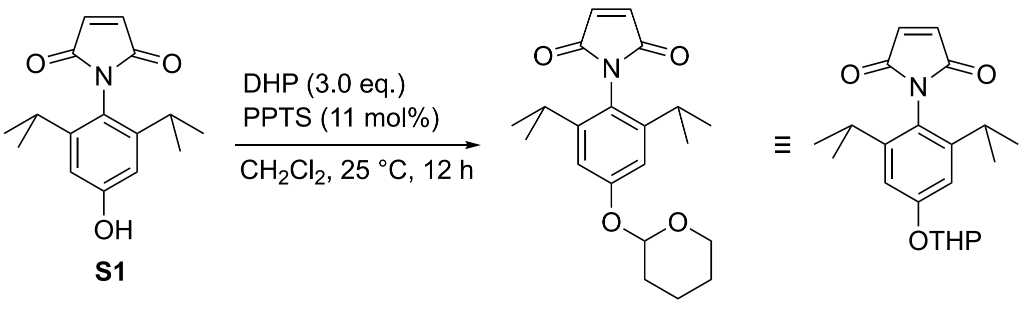

S2

_N_-(2,6-Diisopropyl-4-((tetrahydro-2*H*-pyran-2-yl)oxy)phenyl)maleimide (S2)

Under Ar atmosphere, **S1** (3.08 g, 11.3 mmol) and pyridinium *p*-toluenesulfonate (PPTS, 310 mg, 1.23 mmol) were added to a round-bottom flask (300 mL). After addition of CH_2Cl_2 (85 mL) and 3,4-dihydro-2*H*-pyran (DHP, 2.87 mL, 33.4 mmol) at 0 °C, the reaction mixture was stirred at 25 °C for 12 h. The reaction mixture was quenched with H_2O and poured into a separatory funnel, then washed with brine, dried over anhydrous Na_2SO_4 and evaporated. Recrystallization from CH_2Cl_2/*n*-hexane furnished **S2** as a white solid (3.86 g, 96%).

^{1}H NMR (600 MHz, $CDCl_3$) δ (ppm) 6.92 (s, 2H), 6.86 (s, 2H), 5.45 (m, 1H), 3.92 (m, 1H), 3.63 (m, 1H), 2.57 (sept, J = 6.9 Hz, 2H), 2.02 (m, 1H), 1.87 (m, 2H), 1.67–1.69 (m, 2H), 1.62–1.63 (m, 1H) and 1.13 (d, J = 6.9 Hz, 12H); ^{13}C NMR (151 MHz, $CDCl_3$) δ (ppm) 170.94, 158.76, 148.89, 134.36, 119.52, 112.33, 96.34, 61.94, 30.49, 29.55, 25.36, 23.95 and 18.71; HR-APCI TOF-MS (*m*/*z*) found 357.1946, calcd for $C_{21}H_{27}NO_4$: 357.1946 $[M]^-$.

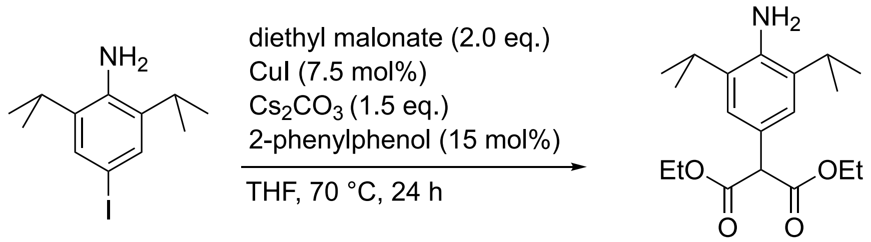

S3

Diethyl 2-(4-amino-3,5-diisopropylphenyl)malonate (S3)

Under Ar atmosphere, 4-iodo-2,6-diisopropylaniline[2] (6.06 g, 20.0 mmol), CuI (285 mg, 1.50 mmol), Cs_2CO_3 (9.78 g, 30.0 mmol) and 2-phenylphenol (510 mg, 3.00 mmol) were added to a round-bottom flask (100 mL). After addition of THF (20 mL) and diethyl malonate (6.10 mL, 40.0 mmol), the reaction mixture was stirred at 70 °C for 24 h. The reaction mixture was quenched with H_2O and diluted with EtOAc. The resulting mixture was poured into a separatory funnel, washed with brine, dried over anhydrous Na_2SO_4 and evaporated. The crude product was purified by silica gel column chromatography (eluent: CH_2Cl_2) and **S3** was obtained as a brown oil (4.00 g, 59%).

^{1}H NMR (600 MHz, $CDCl_3$) δ (ppm) 7.04 (s, 2H), 4.50 (s, 1H), 4.25–4.16 (m, 4H), 3.75 (s, 2H), 2.90 (sept, J = 6.9 Hz, 2H) and 1.28–1.25 (m, 18H); ^{13}C NMR (151 MHz, $CDCl_3$) δ (ppm) 168.96, 140.43, 132.45, 124.04, 122.55, 61.61, 58.06, 28.16, 22.50 and 14.21; HR-APCI TOF-MS (*m*/*z*) found 336.2183, calcd for $C_{19}H_{30}NO_4$: 336.2169 $[M+H]^+$.

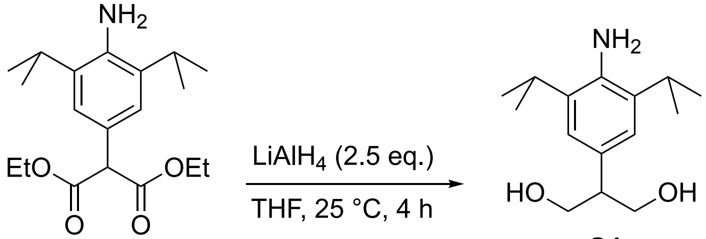

S4

2-(4-Amino-3,5-diisopropylphenyl)propane-1,3-diol (S4)

Under Ar atmosphere, to the suspension of $LiAlH_4$ (396 mg, 10.4 mmol) in THF (10 mL), a THF (10 mL) solution of **S3** (1.52 g, 4.18 mmol) was added at 0 °C. The mixture was stirred at 25 °C for 4 h. The reaction mixture was quenched with saturated aqueous Na_2SO_4, filtered to remove aluminum salts, and washed with EtOAc (50 mL). The filtrate was purified by silica gel column chromatography (eluent: EtOAc). Recrystallization from CH_2Cl_2/*n*-hexane furnished **S4** as a white solid (910 mg, 87%).

^{1}H NMR (600 MHz, $CDCl_3$) δ (ppm) 6.87 (s, 2H), 3.98–3.94 (m, 2H), 3.91–3.88 (m, 2H), 3.70 (s, 2H), 3.03 (tt, J = 7.8, 5.4 Hz, 1H), 2.92 (sept, J = 6.6 Hz, 2H), 1.92 (t, J = 5.4 Hz, 2H) and 1.26 (d, J = 6.6 Hz, 12H); ^{13}C NMR (151 MHz, $CDCl_3$) δ (ppm) 139.44, 132.97, 128.69, 122.43, 66.55, 49.75, 28.09 and 22.52; HR-APCI TOF-MS (*m*/*z*) found 252.1968, calcd for $C_{15}H_{26}NO_2$: 252.1958 $[M+H]^+$.

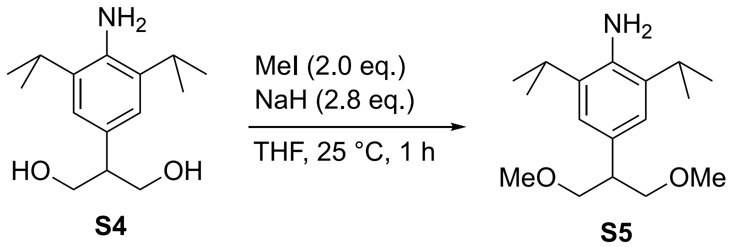

4-(1,3-Dimethoxypropan-2-yl)-2,6-diisopropylaniline (S5)

Under Ar atmosphere, to the suspension of NaH (60% dispersion in paraffin liquid, 401 mg, 10.0 mmol) and MeI (445 μL, 7.16 mmol) in THF (9.0 mL), a THF (4.5 mL) solution of **S4** (900 mg, 3.58 mmol) was added at 0 °C. The mixture was stirred at 25 °C for 1 h. The reaction mixture was diluted with EtOAc (20 mL) and evaporated. The crude product was purified by silica gel column chromatography (eluent: CH_2Cl_2:*n*-hexane:EtO_2 = 1:1:1 by volume) to furnish **S5** as brown oil (932 mg, 93%).

^{1}H NMR (600 MHz, $CDCl_3$) δ (ppm) 6.92 (s, 2H), 3.63 (dd, J = 9.2, 6.8 Hz, 2H), 3.57 (dd, J = 9.7, 6.4 Hz, 2H), 3.39 (s, 6H), 3.06 (tt, J = 9.2, 6.4 Hz, 1H), 2.91 (sept, J = 6.9 Hz, 2H), and 1.27 (d, J = 6.9 Hz, 12H); ^{13}C NMR (151 MHz, $CDCl_3$) δ (ppm) 139.12, 132.45, 130.54, 122.43, 74.79, 58.99, 45.84, 28.13 and 22.56; HR-APCI TOF-MS (*m*/*z*) found 280.2271, calcd for $C_{17}H_{30}NO_2$: 280.2271 $[M+H]^+$.

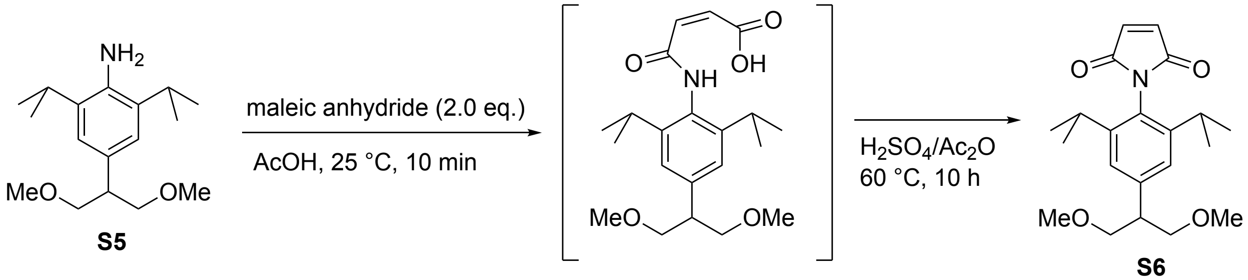

***N*-(4-(1,3-Dimethoxypropan-2-yl)-2,6-diisopropylphenyl)maleimide (S6)**

Under Ar atmosphere, **S5** (1.03 g, 3.69 mmol) and maleic anhydride (723 mg, 7.37 mmol) were added to a Schlenk flask (20 mL). After addition of AcOH (2.21 mL), the reaction mixture was stirred at 25 °C for 10 min. Then, H_2SO_4 (95%, 369 μL) and acetic anhydride (184 μL) were added to the reaction mixture, and the reaction vessel was warmed at 60 °C for 10 h. The reaction mixture was quenched with H_2O and diluted with EtOAc. The solution was poured into a separatory funnel, washed with brine, dried over anhydrous Na_2SO_4 and evaporated. The crude product was purified by silica gel column chromatography (eluent: CH_2Cl_2/Et_2O = 6/1 by volume) and **S6** was obtained as a white solid (1.06 g, 80%).

^{1}H NMR (600 MHz, $CDCl_3$) δ (ppm) 7.12 (s, 2H), 6.88 (s, 2H), 3.67–3.64 (m, 2H), 3.63–3.60 (m, 2H), 3.35 (s, 6H), 3.13 (tt, J =7.2, 6.0 Hz, 1H), 2.59 (sept, J = 6.9 Hz, 2H) and 1.15 (d, J = 6.9 Hz, 12H); ^{13}C NMR (151 MHz, $CDCl_3$) δ (ppm) 170.84, 147.24, 142.95, 134.43, 124.79, 123.94, 74.04, 59.09, 46.37, 29.45, and 24.09; HR-APCI TOF-MS (*m*/*z*) found 359.2096, calcd for $C_{21}H_{30}NO_4$: 359.2091 $[M]^+$.

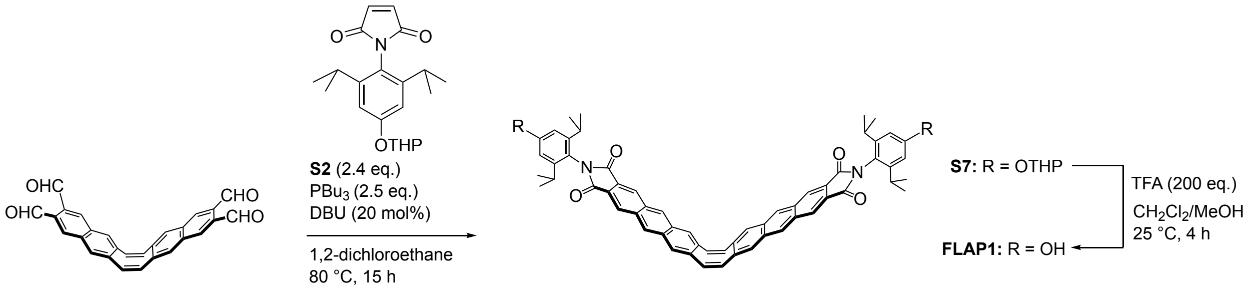

FLAP1

Under Ar atmosphere, **S2** (559 mg, 1.56 mmol) and 1,2-dichloroethane (12 mL) were added to a round-bottom flask (20 mL). After addition of tributylphosphine (410 µL, 1.66 mmol) at 0 °C, the reaction mixture was stirred at 25 °C for 30 min. A 1,2-dichloroethane (10 mL) solution of tetraformyl dinaphthocyclooctatetraene[3,4] (271 mg, 0.651 mmol) was added to the reaction mixture at 0 °C, followed by addition of DBU (19.5 µL, 0.130 mmol). The reaction mixture was stirred at 80 °C for 15 h, and then quenched with H_2O. The resulting mixture was poured into a separatory funnel, washed with brine, dried over anhydrous Na_2SO_4 and evaporated. The crude product was purified by silica gel column chromatography (eluent: CH_2Cl_2/AcOEt = 20/1 by volume), and **S7** was obtained as a yellow solid (138 mg, 20% from tetraformyl dinaphthocyclooctatetraene), which was used for the subsequent reaction. **S7** was added to a round-bottom flask (100 mL). After addition of CH_2Cl_2 (30 mL), MeOH (10 mL), and TFA (2.0 mL, 26 mmol), the reaction mixture was stirred at 25 °C for 4 h. The reaction mixture was quenched with H_2O and poured into a separatory funnel, then washed with brine, dried over anhydrous Na_2SO_4 and evaporated. The crude product was purified by silica gel column chromatography (eluent: CH_2Cl_2/EtOAc = 20/1 by volume). Recrystallization from CH_2Cl_2/*n*-hexane furnished **FLAP1** as a yellow solid (107 mg, 92% from **S7**).

^{1}H NMR (600 MHz, $CDCl_3$) δ (ppm) 8.55 (m, 4H + 4H), 7.94 (s, 4H), 7.24 (s, 4H), 6.75 (s, 4H), 4.90 (s, 2H), 2.70 (sept, J = 6.9 Hz, 4H) and 1.14 (d, J = 6.9 Hz, 24H); ^{13}C NMR (151 MHz, DMSO-d_6) δ (ppm) 167.47, 158.59, 147.92, 136.12, 133.09, 131.88, 131.44, 129.93, 128.34, 126.56, 125.73, 118.40, 110.50, 28.65 and 23.56; HR-MALDI TOF-MS (*m*/*z*) found 895.33, calcd for $C_{60}H_{51}N_2O_6$: 895.37 $[M+H]^+$. UV/visible absorption (CH_2Cl_2): λ_{max} (ε /M^{-1} cm^{-1}) = 332 nm (9.28×10^4); fluorescence (CH_2Cl_2, λ_{ex} = 340 nm): λ_{max} = 523, 564, and 610 nm, Φ_{FL} = 0.26.

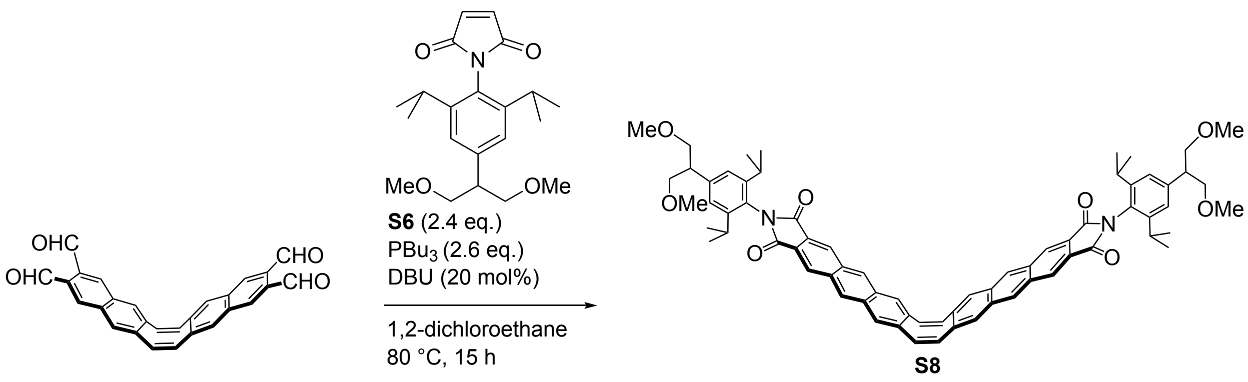

S8

Under Ar atmosphere, **S6** (682 mg, 1.90 mmol) and 1,2-dichloroethane (30 mL) were added to a round-bottom flask (200 mL). After addition of tributylphosphine (507 μL, 2.05 mmol) at 0 °C, the reaction mixture was stirred at 25 °C for 30 min. Then, a 1,2-dichloroethane (15 mL) solution of tetraformyl dinaphthocyclooctatetraene (329 mg, 0.79 mmol) was added to the reaction mixture at 0 °C, followed by addition of DBU (24 μL, 0.16 mmol). The reaction mixture was stirred at 80 °C for 12 h, and then quenched with H_2O. The resulting mixture was poured into a separatory funnel, washed with brine, dried over anhydrous Na_2SO_4 and evaporated. The crude product was purified by silica gel column chromatography (eluent: CH_2Cl_2/Et_2O = 5:1 by volume) and **S8** was obtained as a yellow solid (208 mg, 25% from tetraformyl dinaphthocyclooctatetraene).

^{1}H NMR (600 MHz, $CDCl_3$) δ (ppm) 8.55 (s, 4H + 4H), 7.94 (s, 4H), 7.24 (s, 4H), 7.17 (s, 4H), 3.69–3.62 (m, 8H), 3.37 (s, 12H), 3.16 (tt, J =7.2, 6.0 Hz, 2H), 2.72 (sept, J = 6.8 Hz, 4H) and 1.15 (d, J = 6.8 Hz, 24H); ^{13}C NMR (151 MHz, $CDCl_3$) δ (ppm) 167.88, 146.68, 142.80, 136.63, 133.36, 132.39, 132.20, 129.84, 128.49, 126.64, 125.92, 123.88, 123.56, 74.16, 59.07, 46.37, 29.48, and 24.12; HR-MALDI TOF-MS (m/z) found 1089.53, calcd for $C_{70}H_{70}N_2O_8Na$: 1089.50 $[M+Na]^+$.

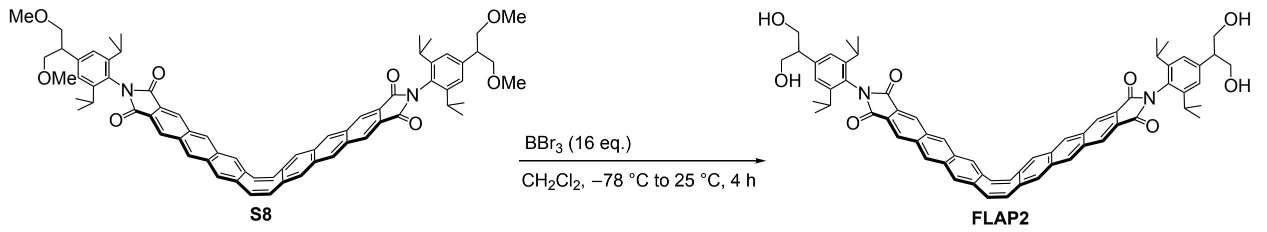

FLAP2
Under Ar atmosphere, **S8** (9.8 mg, 9.2 μmol) and CH_2Cl_2 (0.7 mL) were added to a Schlenck flask (20 mL). After addition of a CH_2Cl_2 solution of BBr_3 (1.0 M, 147 μL) at –78 °C, the reaction mixture was stirred and warmed to 25 °C over 4 h. The reaction mixture was quenched with H_2O and poured into a separatory funnel, then washed with brine, dried over anhydrous Na_2SO_4 and evaporated. The crude product was purified by silica gel column chromatography (eluent: EtOAc) and **FLAP2** was obtained as a yellow solid (6.7 mg, 72%).
^{1}H NMR (600 MHz, $CDCl_3$) δ (ppm) 8.56 (s, 4H), 8.55 (s, 4H), 7.94 (s, 4H), 7.24 (s, 4H), 7.14 (s, 4H), 4.05–4.00 (m, 8H), 3.18–3.14 (m, 2H), 2.74 (sept, J = 6.8 Hz, 4H), 2.01 (t, J = 6.0 Hz, 4H) and 1.16 (d, J = 6.8 Hz, 24H); ^{13}C NMR (151 MHz, DMSO-d_6) δ (ppm) 167.30, 145.81, 143.99, 136.18, 133.13, 131.89, 131.47, 129.99, 128.37, 126.76, 125.69, 125.42, 123.63, 62.64, 50.82, 28.70, and 23.67; HR-MALDI TOF-MS (m/z) found 1033.42, calcd for $C_{66}H_{62}N_2O_8Na$: 1033.44 $[M+Na]^+$. UV/visible absorption (CH_2Cl_2): λ_{max} (ε /M^{-1} cm^{-1}) = 331 nm (9.37×10^4); fluorescence (CH_2Cl_2, λ_{ex} = 340 nm): λ_{max} = 524, 565, and 610 nm, Φ_{FL} = 0.32.

Photophysical properties of parent FLAP molecules

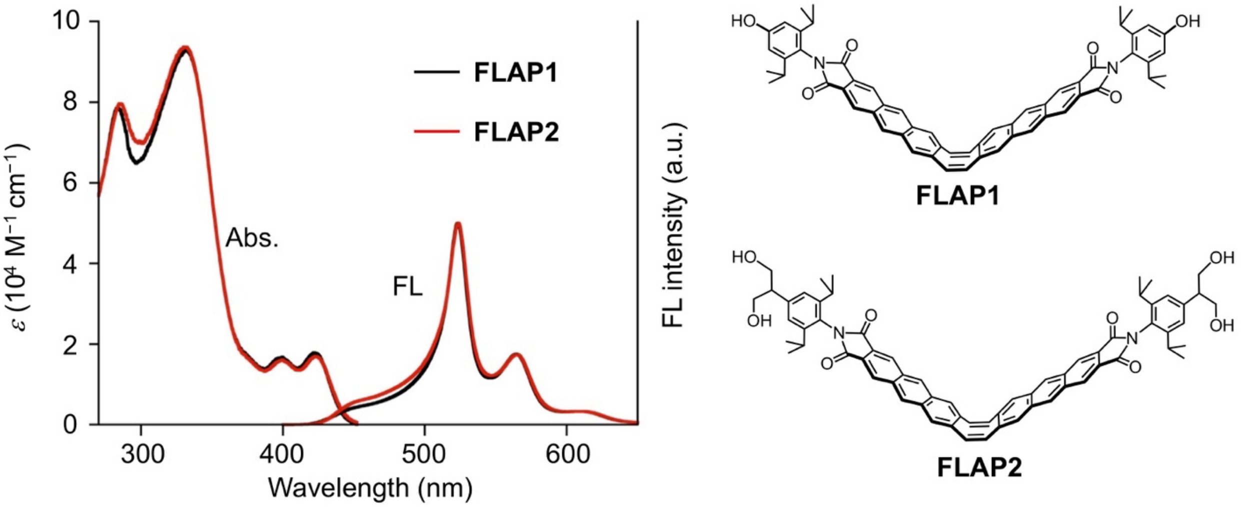

Supplementary Fig. 1. UV/visible absorption (Abs) and FL spectra of **FLAP1** and **FLAP2** in CH_2Cl_2.

Supplementary Table 1. Photophysical constants of **FLAP1** and **FLAP2** in CH_2Cl_2.
λ_{ex} = 365 nm.

	Φ_{FL}	τ_{FL} (ns) [a]	k_r (s^{-1}) [b]	k_{nr} (s^{-1}) [c]
**FLAP1**	0.26	11.0	2.4×10^7	6.7×10^7
**FLAP2**	0.32	10.6	3.0×10^7	6.4×10^7

[a] FL lifetime monitored at 525 nm. [b] Radiative decay constant. [c] Nonradiative decay constant.
k_r and k_{nr} were estimated from the equations below.

$\Phi_{FL} = k_r / (k_r + k_{nr})$
$\tau_{FL} = 1/ (k_r + k_{nr})$

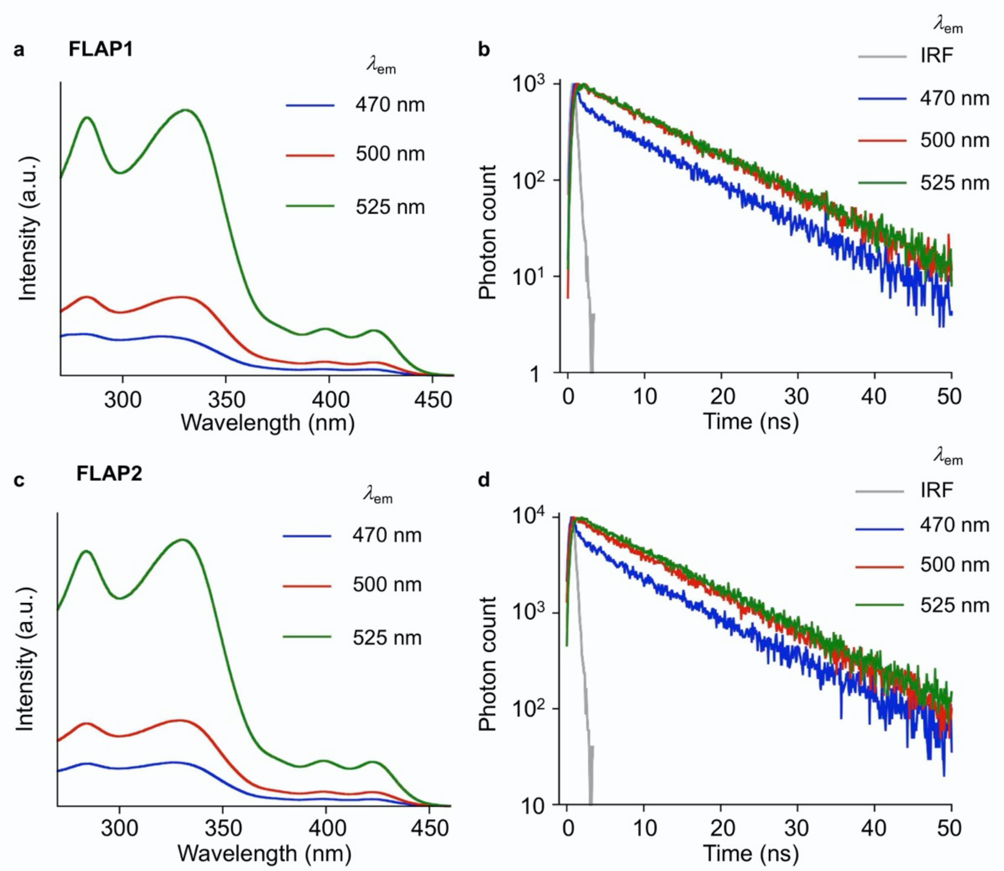

Supplementary Fig. 2. (a) Excitation spectra and (b) FL decay profiles of **FLAP1** in CH_2Cl_2, monitored at different emission wavelengths, and (c, d) those of **FLAP2**. Concentration: *ca.* 10^{-6} M. IRF: The instrument response function.

Supplementary Table 2. Photophysical constants of **FLAP1** and **FLAP2** in CH_2Cl_2. λ_{ex} = 365 nm.

	λ_{em} (nm)	χ^2	τ_{FL} (ns)[a]	τ_1 (ns)	τ_2 (ns)	A_1	A_2
**FLAP1**	470	1.06	9.0	0.2	9.9	320	66
	500	1.16	10.6	0.3	10.8	88	110
	525	1.03	11.0				
**FLAP2**	470	1.25	8.9	0.2	9.8	270	66
	500	1.07	10.4	0.3	10.7	94	104
	525	1.05	10.6				

[a] Mean FL lifetime calculated from the equation below.

$$\tau_{FL} = \frac{{\tau_1}^2 A_1 + {\tau_2}^2 A_2}{\tau_1 A_1 + \tau_2 A_2}$$

where A_1 and A_2 was determined by analysis of the fitting equation below.

$$G(t) = A_1 \exp\left(-\frac{t}{\tau_1}\right) + A_2 \exp\left(-\frac{t}{\tau_2}\right)$$

where $G(t)$ was photon count as a function of time.

Computational studies of FLAP molecules

Density functional theory (DFT) and time-dependent (TD) DFT calculations of the isolated molecules were performed using the Gaussian 16 program.[5] The density functional of PBE0 was selected due to the good agreement with the absorption spectra of the FLAP series[6]. The basis set of 6-31G(d) or 6-31+G(d) was used for the calculations (see the legend of each figure and table). All the optimized structures gave no imaginary frequency, bearing C_1 symmetry. In the Supplementary Figs. 4–12, compositions (%) and oscillator strength f were calculated based on the respective optimized structure. H and L stand for HOMO and LUMO, respectively. Here and elsewhere, compositions were calculated by taking the square of the dominant configuration and multiplying the result by two, in order to account for excitations involving α and β spin orbitals. In the optimization calculation in S_1, two energy minima at bent and planar geometries were found (Supplementary Figs. 5 and 6). Here the oscillator strength of the S_1 bent geometry was calculated by the PBE0 functional to be zero (Supplementary Table 4), although a non-zero oscillator strength was obtained by a different functional, CAM-B3LYP (Supplementary Fig. 11 and Supplementary Table 10). This indicates that the calculation results of the S_1 electronic state are sensitive to the applied calculation level.

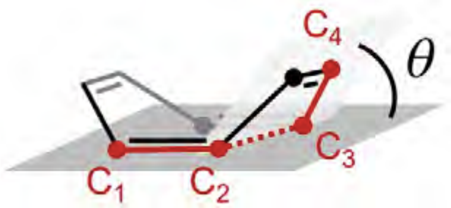

Supplementary Fig. 3. Definition of the COT bending angle θ used in the computational studies. The angle θ was defined as a dihedral angle between the two planes, the C_1–C_2–C_3 plane and the C_2–C_3–C_4 plane.

Calculations performed at the PBE0/6-31+G(d) level of theory

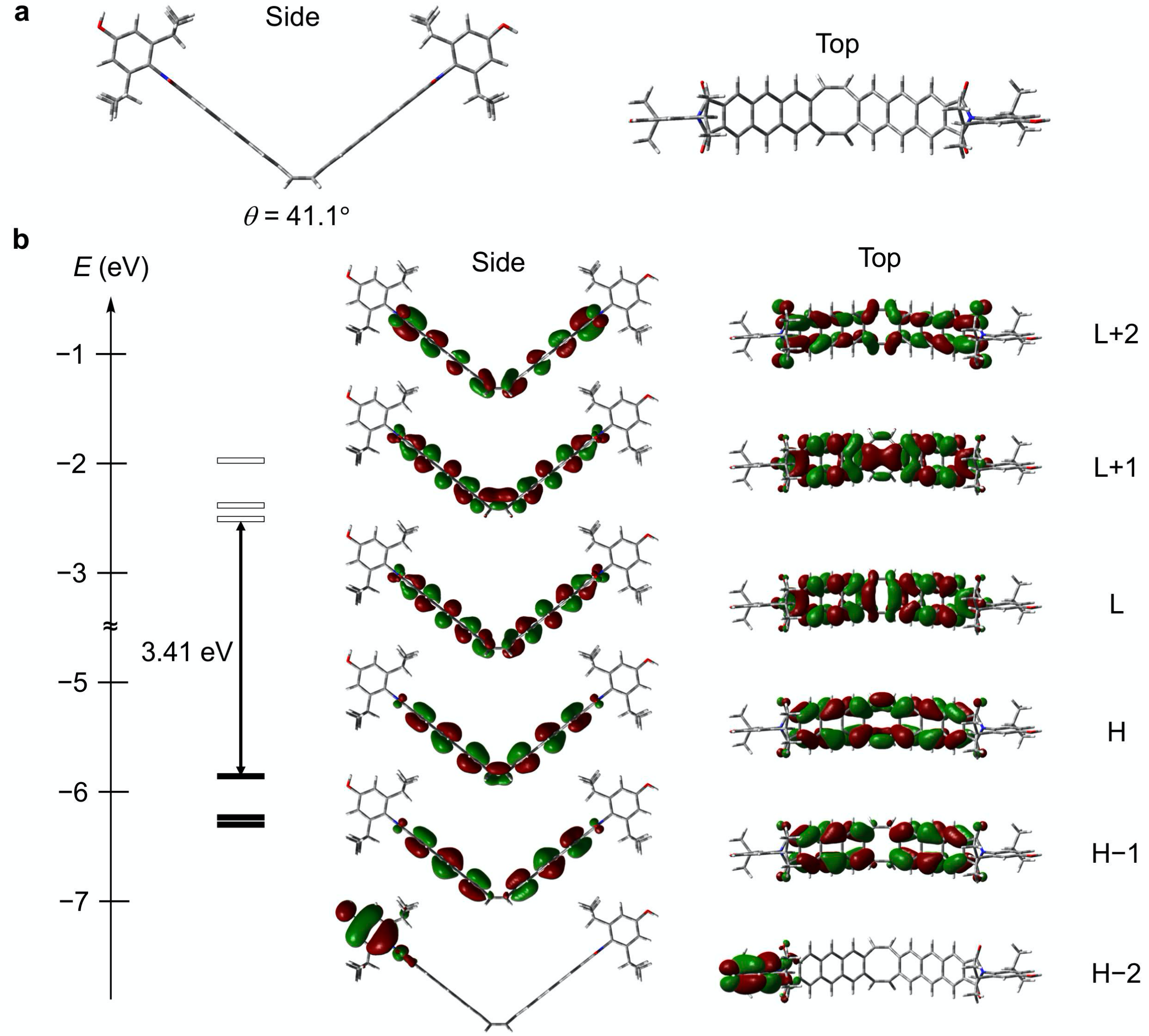

Supplementary Fig. 4. (a) S_0 optimized geometry of **FLAP1** at the PBE0/6-31+G(d) level and (b) the representative molecular orbitals and their energy levels.

Supplementary Table 3. Excitation energies, configurations, and oscillator strengths of the $S_0 \rightarrow S_n$ ($n \leq 3$) transitions for the S_0 optimized **FLAP1** at the TD PBE0/6-31+G(d) level.

Transition	Excitation energy	Configuration(s)	Oscillator strength
$S_0 \rightarrow S_1$	2.89 eV (429 nm)	H → L (96%)	0.0000
$S_0 \rightarrow S_2$	2.99 eV (415 nm)	H−1 → L (7%)<br>H → L+1 (93%)	0.0385
$S_0 \rightarrow S_3$	3.19 eV (389 nm)	H−7 → L+1 (4%)<br>H−6 → L (16%)<br>H−1 → L+3 (8%)<br>H → L+2 (68%)	0.2660

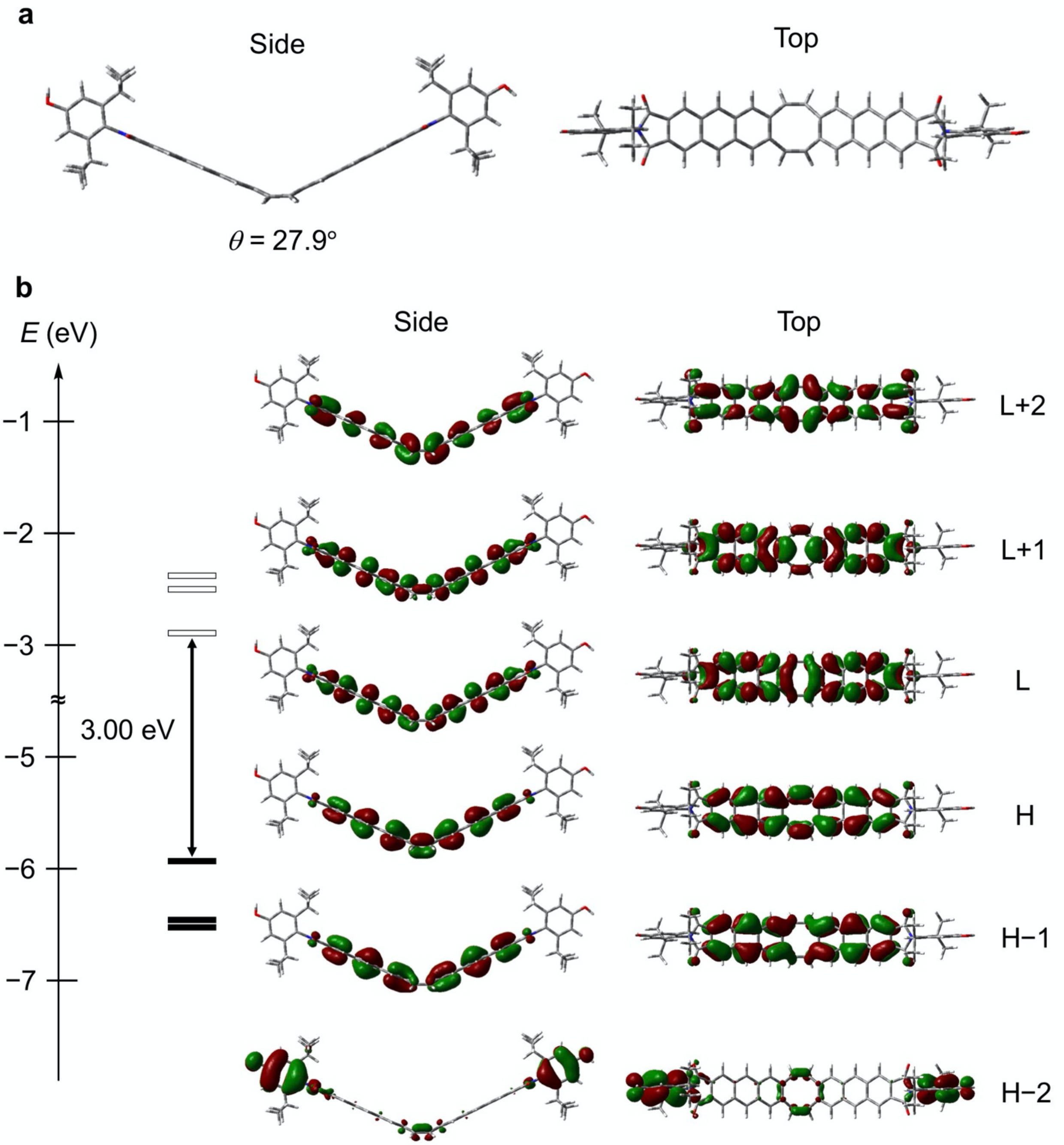

Supplementary Fig. 5. (a) S_1 optimized geometry of **FLAP1** (bent) at the TD PBE0/6-31G+(d) level and (b) the representative molecular orbitals and their energy levels.

Supplementary Table 4. Excitation energy, configuration, and oscillator strength of the $S_0 \rightarrow S_1$ transition for the S_1 optimized **FLAP1** (bent) at the TD PBE0/6-31+G(d) level.

Transition	Excitation energy	Configuration	Oscillator strength
$S_0 \rightarrow S_1$	2.52 eV (492 nm)	H → L (98%)	0.0000

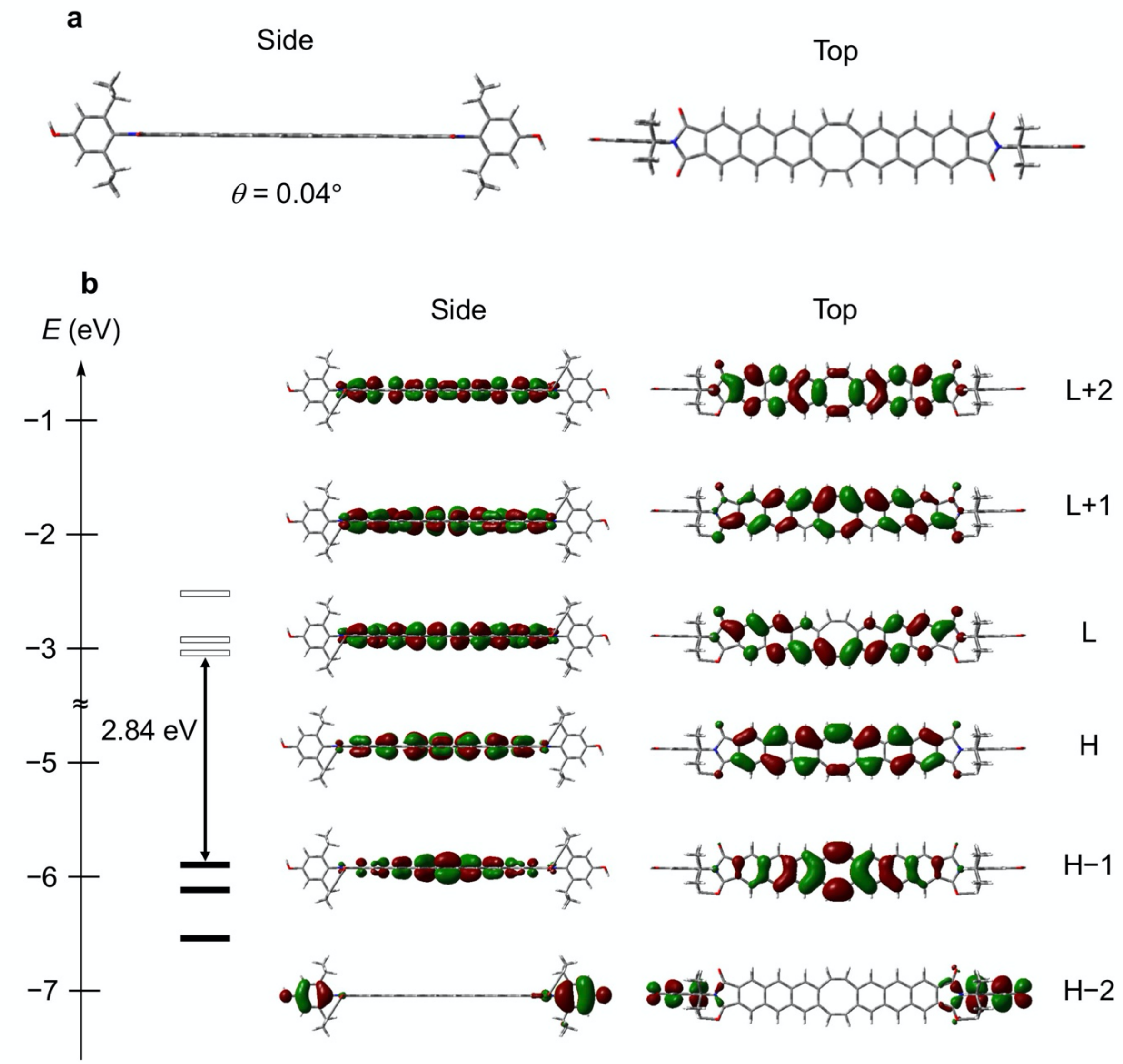

Supplementary Fig. 6. (a) S_1 optimized geometry of **FLAP1** (planar) at the TD PBE0/6-31G+(d) level and (b) the representative molecular orbitals and their energy levels.

Supplementary Table 5. Excitation energy, configurations, and oscillator strength of the $S_0 \rightarrow S_1$ transition for the S_1 optimized **FLAP1** (planar) at the TD PBE0/6-31+G(d) level.

Transition	Excitation energy	Configurations	Oscillator strength
$S_0 \rightarrow S_1$	2.21 eV (561 nm)	H−1 → L (35%)<br>H → L (44%)<br>H→ L+1 (19%)	0.0731

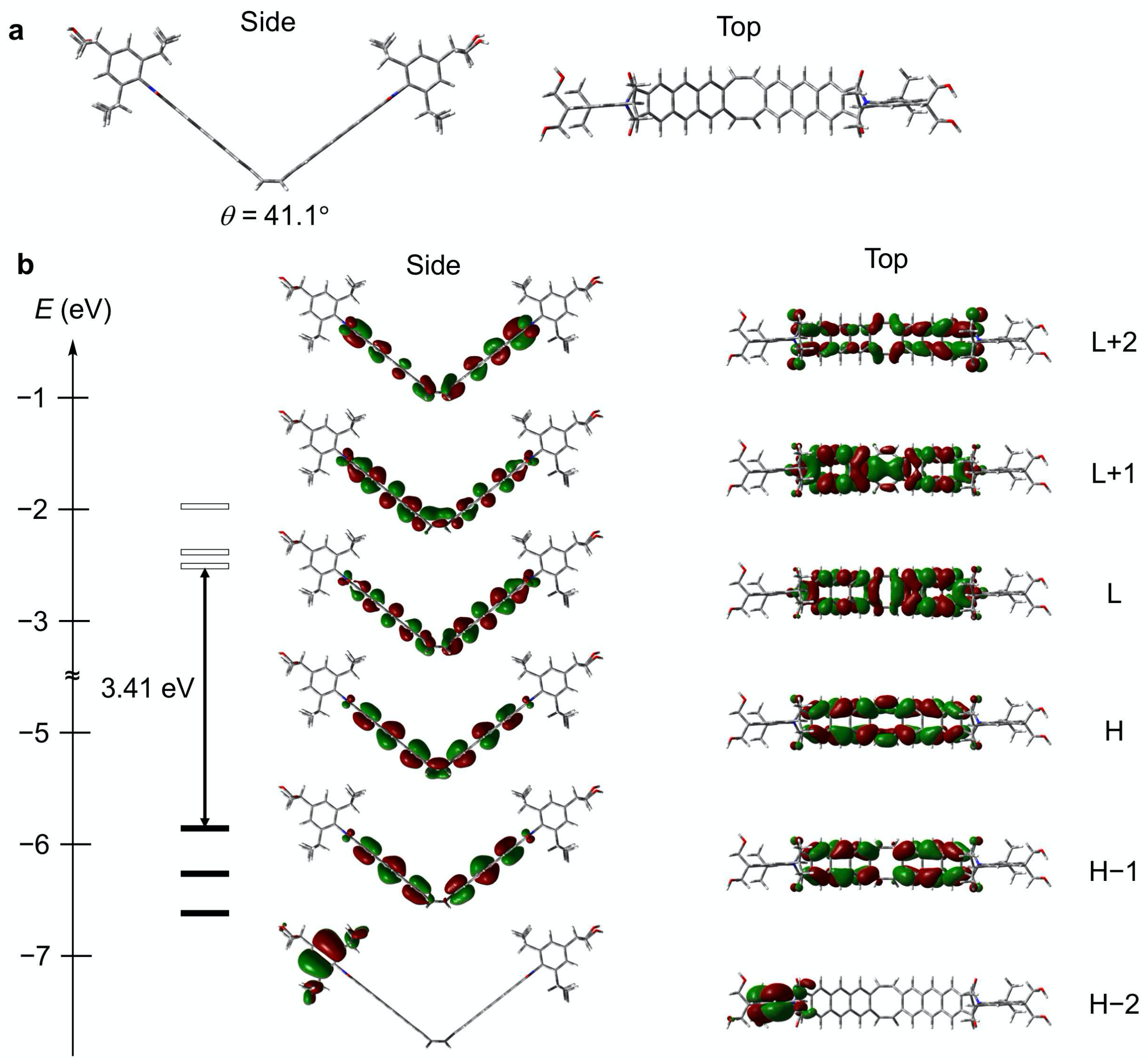

Supplementary Fig. 7. (a) S_0 optimized geometry of **FLAP2** at the PBE0/6-31+G(d) level and (b) the representative molecular orbitals and their energy levels.

Supplementary Table 6. Excitation energies, configurations, and oscillator strengths of the $S_0 \rightarrow S_n$ ($n \leq 3$) transitions for the S_0 optimized **FLAP2** at the TD PBE0/6-31+G(d) level.

Transition	Excitation energy	Configuration(s)	Oscillator strength
$S_0 \rightarrow S_1$	2.89 eV (429 nm)	H → L (96%)	0.0000
$S_0 \rightarrow S_2$	2.99 eV (415 nm)	H−1 → L (7%)<br>H → L+1 (92%)	0.0387
$S_0 \rightarrow S_3$	3.19 eV (389 nm)	H−7 → L+1 (4%)<br>H−6 → L (13%)<br>H−1 → L+3 (8%)<br>H → L+2 (69%)	0.2882

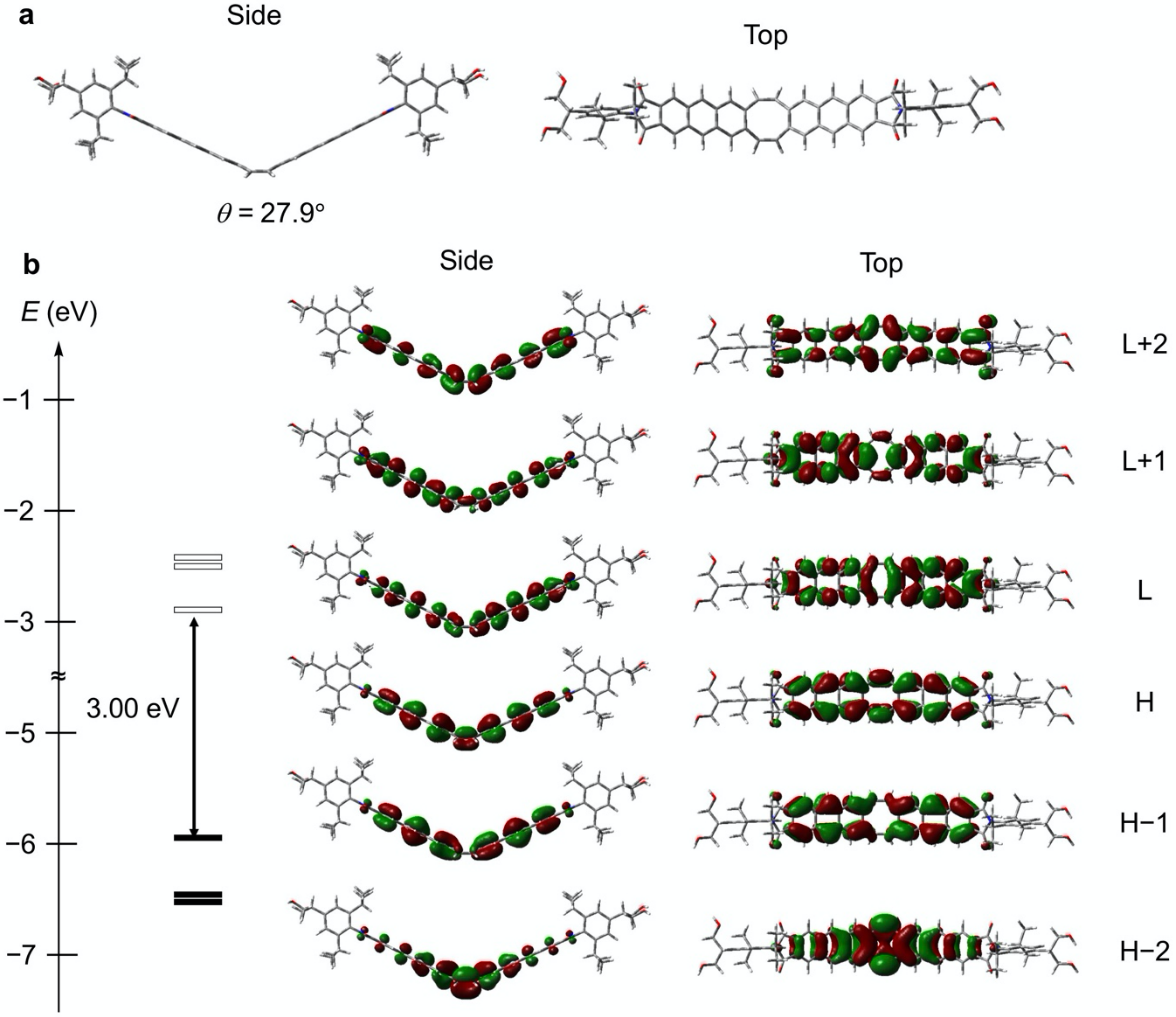

Supplementary Fig. 8. (a) S_1 optimized geometry of **FLAP2** (bent) at the TD PBE0/6-31G+(d) level and (b) the representative molecular orbitals and their energy levels.

Supplementary Table 7. Excitation energy, configuration, and oscillator strength of the $S_0 \rightarrow S_1$ transition for the S_1 optimized **FLAP2** (bent) at the TD PBE0/6-31+G(d) level.

Transition	Excitation energy	Configuration	Oscillator strength
$S_0 \rightarrow S_1$	2.52 eV (492 nm)	H → L (98%)	0.0005

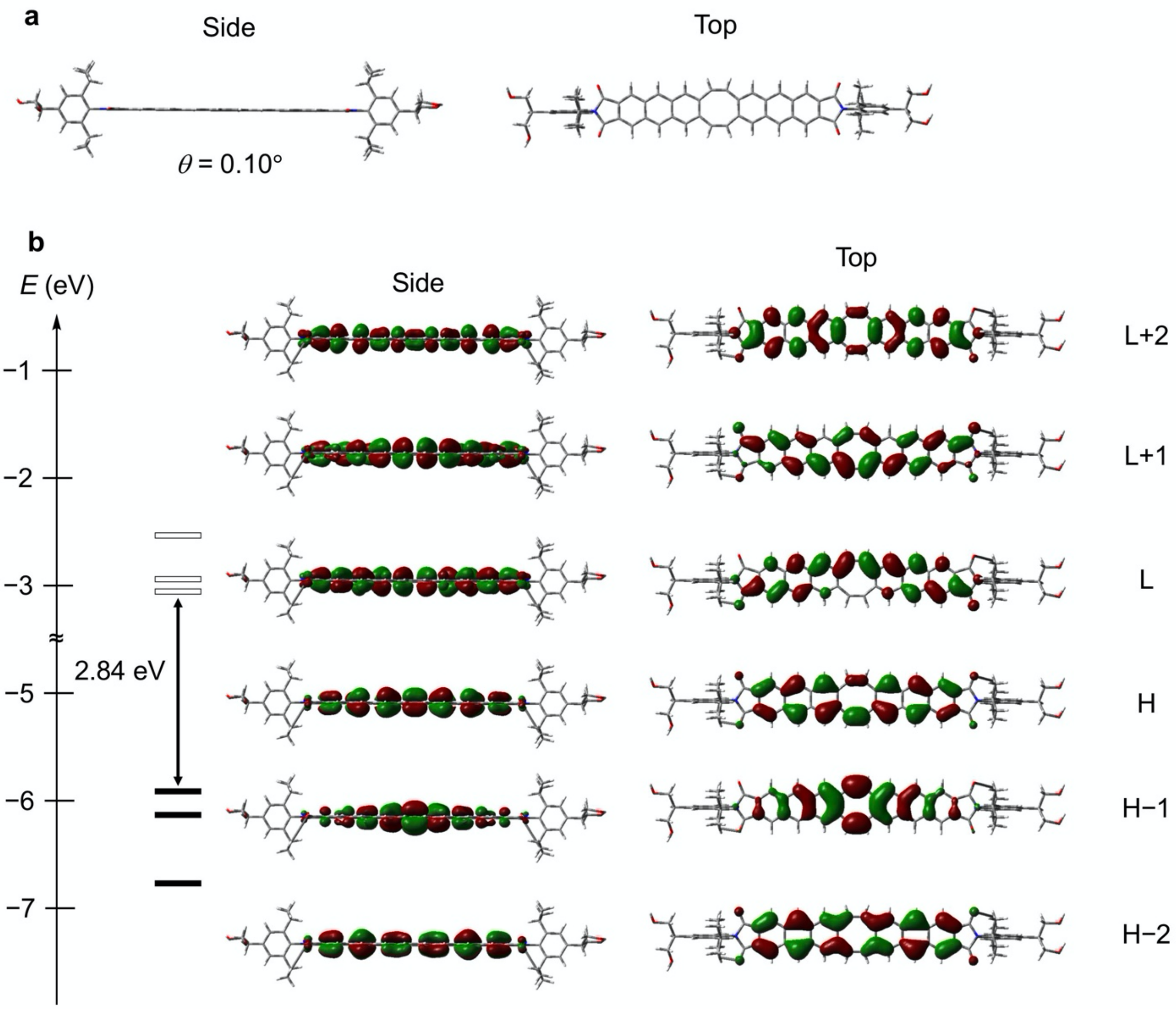

Supplementary Fig. 9. (a) S_1 optimized planar geometry of **FLAP2** (planar) at the TD PBE0/6-31G+(d) level and (b) representative molecular orbitals and their energy levels.

Supplementary Table 8. Excitation energy, configurations, and oscillator strength of the $S_0 \rightarrow S_1$ transition for planar S_1 optimized **FLAP2** at the TD PBE0/6-31+G(d) level.

Transition	Excitation energy	Configurations	Oscillator strength
$S_0 \rightarrow S_1$	2.21 eV (561 nm)	H−1 → L (35%)<br>H → L (44%)<br>H→ L+1 (19%)	0.0749

Calculations performed at the CAM-B3LYP/6-31+G(d) level of theory

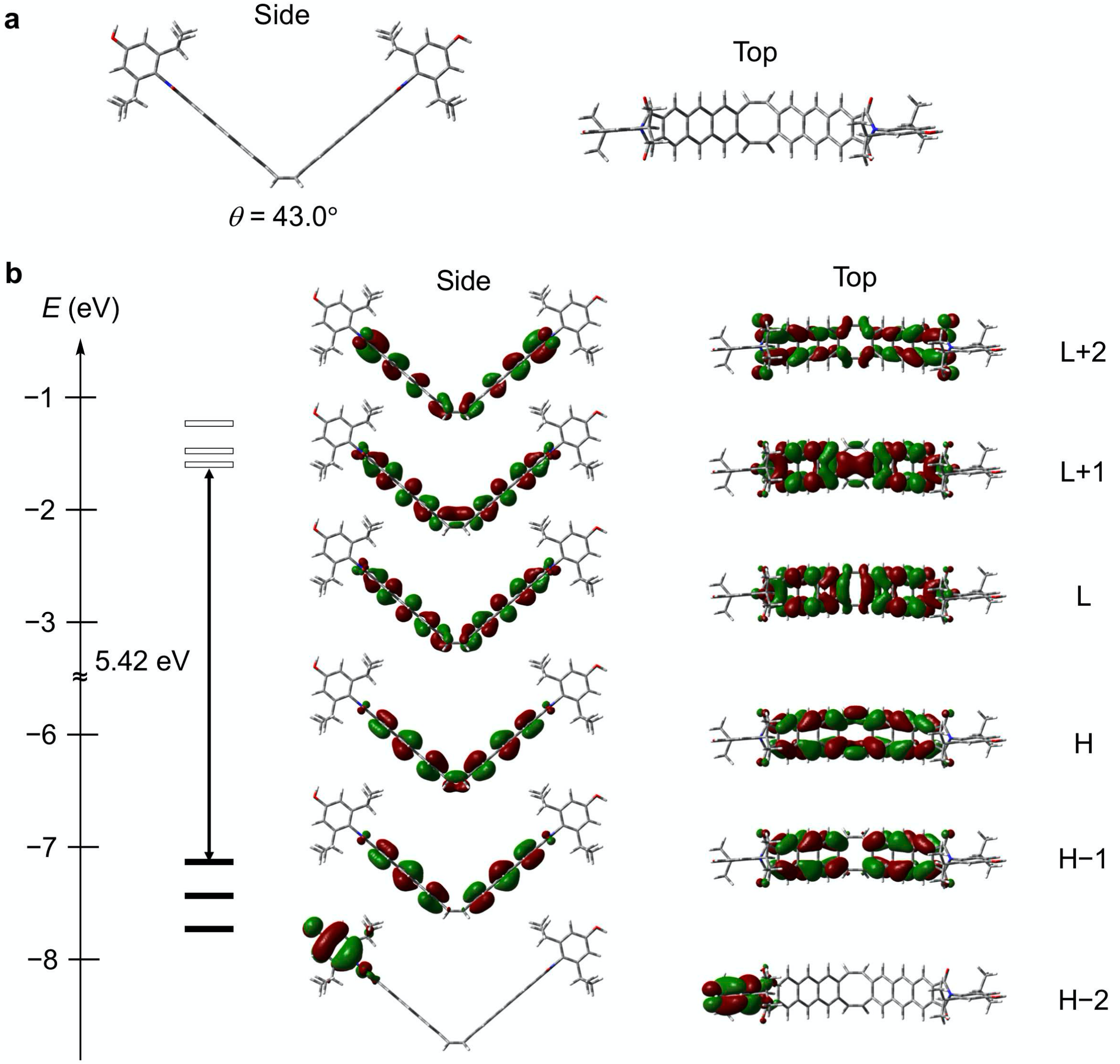

Supplementary Fig. 10. (a) S_0 optimized geometry of **FLAP1** at the CAM-B3LYP/6-31+G(d) level and (b) the representative molecular orbitals and their energy levels.

Supplementary Table 9. Excitation energies, configurations and oscillator strengths of the $S_0 \rightarrow S_n$ ($n \leq 3$) transitions for the S_0 optimized **FLAP1** at the TD CAM-B3LYP/6-31+G(d) level.

Transition	Excitation energy	Configurations	Oscillator strength
$S_0 \rightarrow S_1$	3.38 eV (367 nm)	H−1 → L+1 (33%)<br>H → L (63%)	0.0000
$S_0 \rightarrow S_2$	3.40 eV (365 nm)	H−1 → L (40%)<br>H → L+1 (57%)	0.0847
$S_0 \rightarrow S_3$	3.65 eV (340 nm)	H−7 → L+1 (11%)<br>H−5 → L (15%)<br>H−1 → L+3 (22%)<br>H→ L+2 (43%)	0.2185

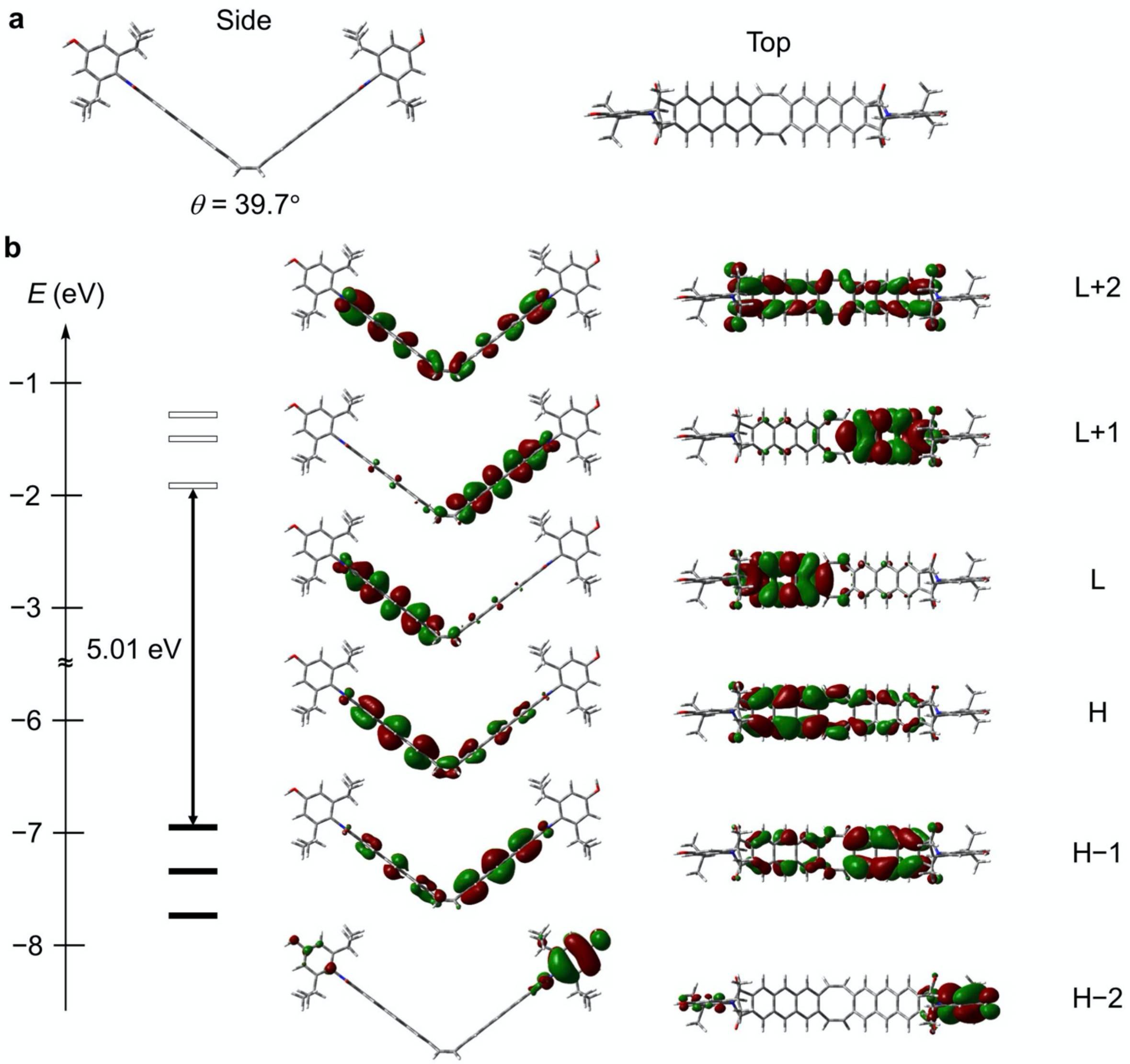

Supplementary Fig. 11. (a) S_1 optimized geometry of **FLAP1** at the TD CAM-B3LYP/6-31G+(d) level and (b) the representative molecular orbitals and their energy levels.

Supplementary Table 10. Excitation energy, configurations, and oscillator strength of the $S_0 \rightarrow S_1$ transition for the S_1 optimized **FLAP1** (bent) at the TD CAM-B3LYP/6-31+G(d) level.

Transition	Excitation energy	Configurations	Oscillator strength
$S_0 \rightarrow S_1$	2.90 eV (428 nm)	H−1 → L (8%)<br>H → L (88%)	0.0423

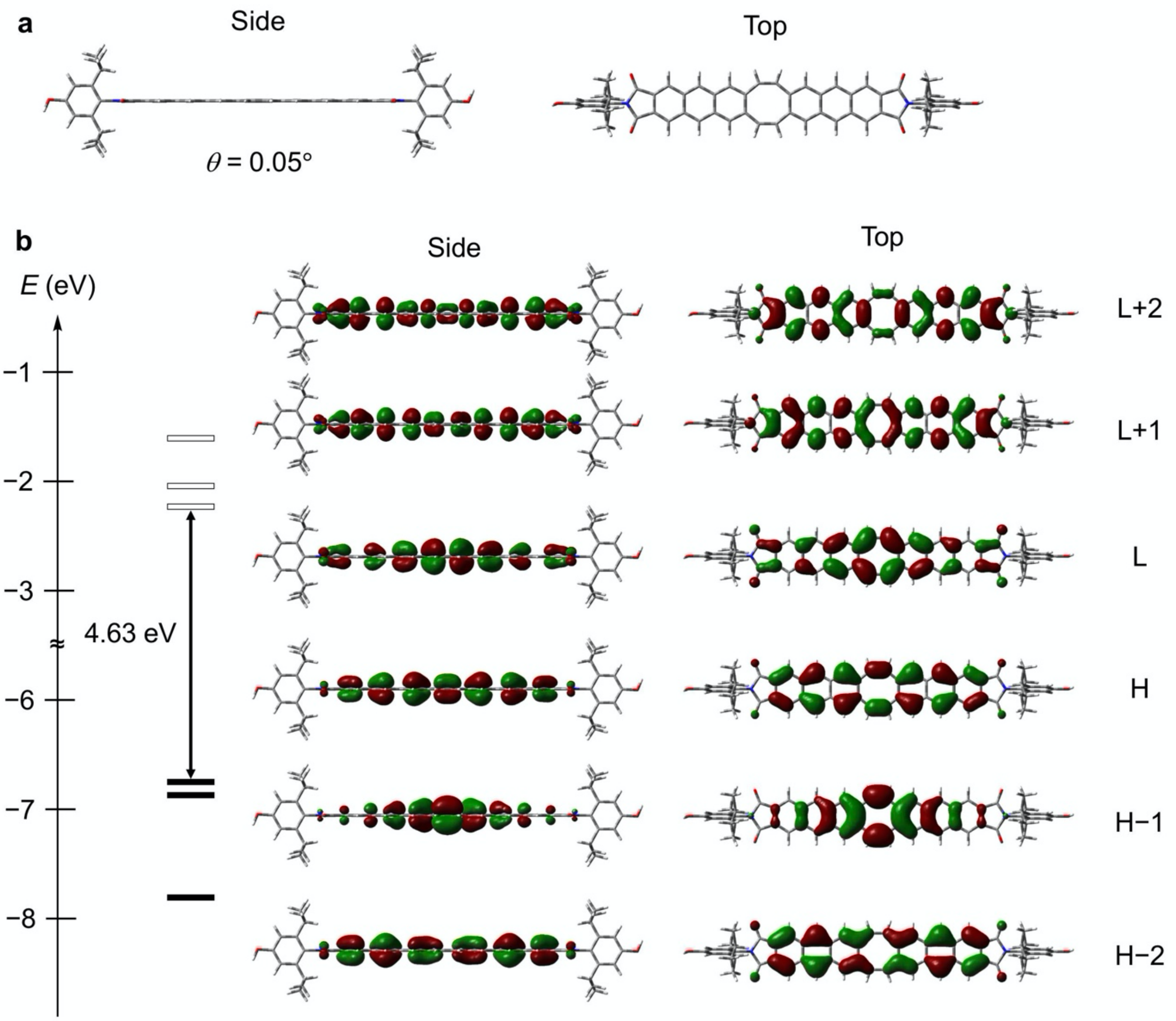

Supplementary Fig. 12. (a) S_1 optimized geometry of **FLAP1** (planar) at the TD CAM-B3LYP/6-31G+(d) level and (b) the representative molecular orbitals and their energy levels.

Supplementary Table 11. Excitation energy, configuration, and oscillator strength of the $S_0 \rightarrow S_1$ transition for the S_1 optimized **FLAP1** (planar) at the TD CAM-B3LYP/6-31+G(d) level.

Transition	Excitation energy	Configuration	Oscillator strength
$S_0 \rightarrow S_1$	2.22 eV (558 nm)	H → L (94%)	0.0000

Computational studies of the polymer-chain substructure in the vicinity of FLAP

The following substructure of a FLAP-doped polyurethane chain, **FLAP1′**, was calculated in Fig. 3 of the main text. The distance between the C^A and C^B atoms was fixed in each optimization by using the *opt=modredundant* keyword of Gaussian16.

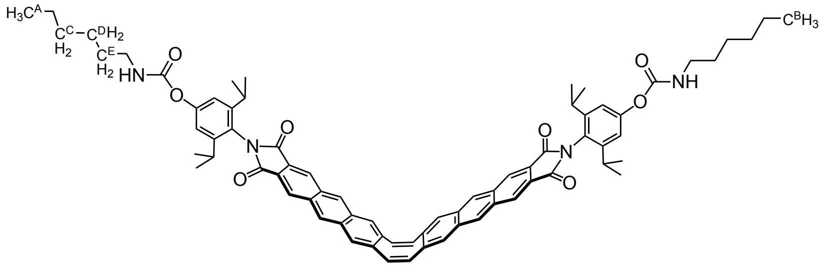

FLAP1′

Supplementary Fig. 13. Substructure of a FLAP-doped polyurethane chain, **FLAP1′**, in Fig. 3.

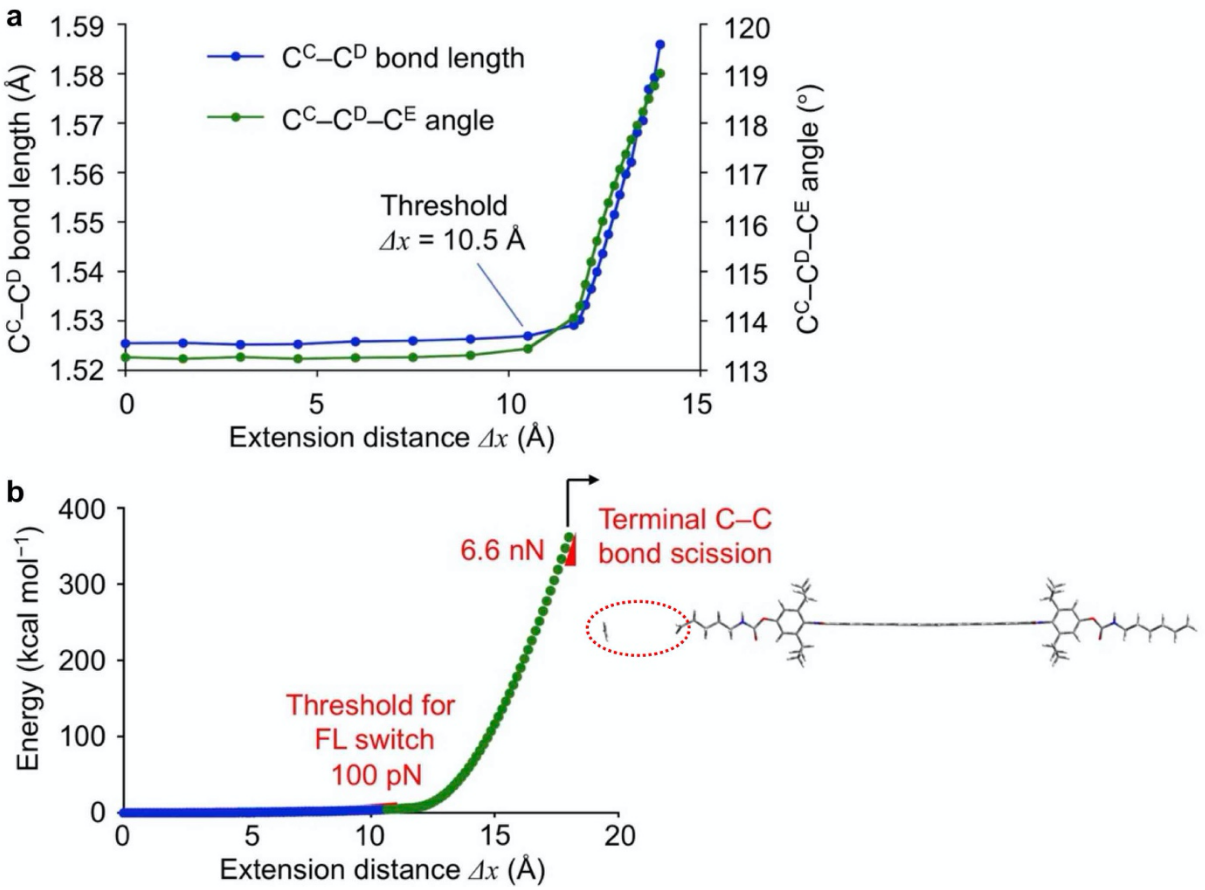

Supplementary Fig. 14. (a) DFT calculated changes in the C^C–C^D bond length and the C^C–C^D–C^E bond angle of **FLAP1′**. The bond length and angle are not remarkably increased at the threshold for the FL switch, indicating the FL response of the FLAP force probe before mechanical damage. (b) Calculated energy diagram of **FLAP1′** until the formal C–C bond scission at the PBE0/6-31G(d) level.

Note: Experimental evidence for such bond elongation has not been demonstrated in the literature.[7] One of the reviewers of this manuscript gave a comment that the C–C bonds are the stiffest elements of the molecule and they will be least strained by applied force; the C–C bond doesn't need to elongate to experience accelerated dissociation, since (a) C–C bond distance and bond dissociation energy don't generally correlate (except in introductory chemistry textbooks), nor should they based on any known law of physics and (b) acceleration of C–C bond homolysis in strained molecules proceeds not by elongation (or "weakening") of the bond but by stabilization of the TS that is longer than the reactant along the pulling axis.

Calculated electronic transitions for the absorption

To interpret the absorption spectral change of the polyurethane film upon mechanical testing, TD-DFT calculations of **FLAP1′** were performed for the unstretched (Δx = 0 Å) and fully stretched (Δx = 13.0 Å) geometries. Details of the calculation results in Fig. 3E are shown below.

Supplementary Table 12. Excitation energies (> 360 nm), configurations, and oscillator strengths of the $S_0 \rightarrow S_n$ transitions for the unstretched (Δx = 0 Å) geometry at the TD PBE0/6-31+G(d) level.

Transition	Excitation energy	Configuration(s)	Oscillator strength
$S_0 \rightarrow S_1$	2.87 eV (431 nm)	H → L (97%)	0.0000
$S_0 \rightarrow S_2$	2.99 eV (415 nm)	H−1 → L (7%)<br>H → L+1 (92%)	0.0377
$S_0 \rightarrow S_3$	3.16 eV (391 nm)	H−6 → L (16%)<br>H−2 → L (2%)<br>H−1 → L+3 (7%)<br>H→ L+2 (69%)	0.2858
$S_0 \rightarrow S_4$	3.20 eV (389 nm)	H−1 → L (91%)<br>H → L+1 (7%)	0.0149
$S_0 \rightarrow S_5$	3.30 eV (376 nm)	H−6 → L+1 (22%)<br>H−2 → L+1 (4%)<br>H−1 → L+2 (29%)<br>H → L+3 (33%)	0.0299
$S_0 \rightarrow S_6$	3.31 eV (375 nm)	H−1 → L+1 (96%)	0.0000

Supplementary Table 13. Excitation energies (> 360 nm), configurations, and oscillator strengths of the $S_0 \rightarrow S_n$ transitions for the fully stretched (Δx = 13.0 Å) geometry at the TD PBE0/6-31+G(d) level.

Transition	Excitation energy	Configurations	Oscillator strength
$S_0 \rightarrow S_1$	2.55 eV (486 nm)	H → L (98%)	0.0000
$S_0 \rightarrow S_2$	2.58 eV (481 nm)	H−1 → L (38%)<br>H → L+1 (59%)	0.1196
$S_0 \rightarrow S_3$	2.89 eV (429 nm)	H−1 → L+1 (95%)<br>H−1→ L+4 (3%)	0.0000
$S_0 \rightarrow S_4$	2.92 eV (425 nm)	H → L+2 (96%)	0.0228
$S_0 \rightarrow S_5$	3.09 eV (401 nm)	H−2 → L+1 (37%)<br>H−1 → L+2 (49%)<br>H→ L+3 (9%)	0.0000
$S_0 \rightarrow S_6$	3.14 eV (395 nm)	H−1 → L (59%)<br>H → L+1 (38%)	5.4348
$S_0 \rightarrow S_7$	3.19 eV (389 nm)	H−2 → L (95%)<br>H → L+2 (3%)	0.0185
$S_0 \rightarrow S_8$	3.39 eV (366 nm)	H−4 → L+2 (10%)<br>H−3→ L (87%)	0.0000
$S_0 \rightarrow S_9$	3.39 eV (366 nm)	H−4 → L (87%)<br>H−3→ L+2 (10%)	0.0236

Synthesis and characterization of the linear polycarbonates (PCs)

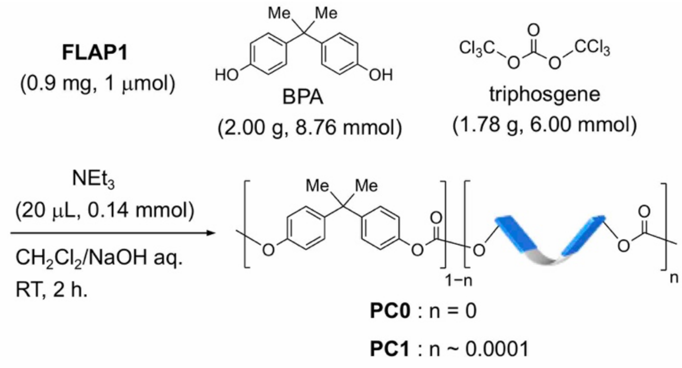

Supplementary Fig. 15. Synthesis of the linear polycarbonates (PCs). The amount of **FLAP1** is given for the **PC1** synthesis, while **PC0** does not contain **FLAP1**.

Bisphenol A (BPA) was recrystallized in advance from AcOH, and washed with H_2O. To the mixture of bisphenol A (BPA) (2.00 g, 8.76 mmol) and NaOH (2.73 g, 68.2 mmol) in H_2O (20 mL), **FLAP1** (0.9 mg, 1 μmol) and triphosgene (2.60 g, 8.76 mmol) in CH_2Cl_2 (20 mL) were added. After addition of triethylamine (TEA) (20 μL, 0.14 mmol), the reaction mixture was stirred vigorously at 25 °C for 2 h. Then, the aqueous layer was removed by decantation and the remaining organic layer was poured into MeOH (100 mL). The precipitation was washed with MeOH (100 mL) followed by H_2O (100 mL), and dried under vacuum to give a lump of **PC1** (≈2.3 g). To obtain **PC0**, the same procedure was performed without **FLAP1**. The resulting linear PCs were soluble in common organic solvents, and therefore shape of the PC samples can be easily designed. By punching a PC sheet, a dumbbell-shaped specimen, JIS K 6251 (No. 3) meeting the requirements of ISO 37, was obtained.

PC0: ^{1}H NMR (600 MHz, $CDCl_3$) δ (ppm) 7.25–7.23 (m, 4H), 7.17–7.15 (m, 4H), and 1.67 (s, 6H).

PC1: ^{1}H NMR (600 MHz, $CDCl_3$) δ (ppm) 7.25–7.24 (m, 4H), 7.17–7.15 (m, 4H), and 1.68 (s, 6H). Note that peaks arising from the FLAP dopant were too small to detect.

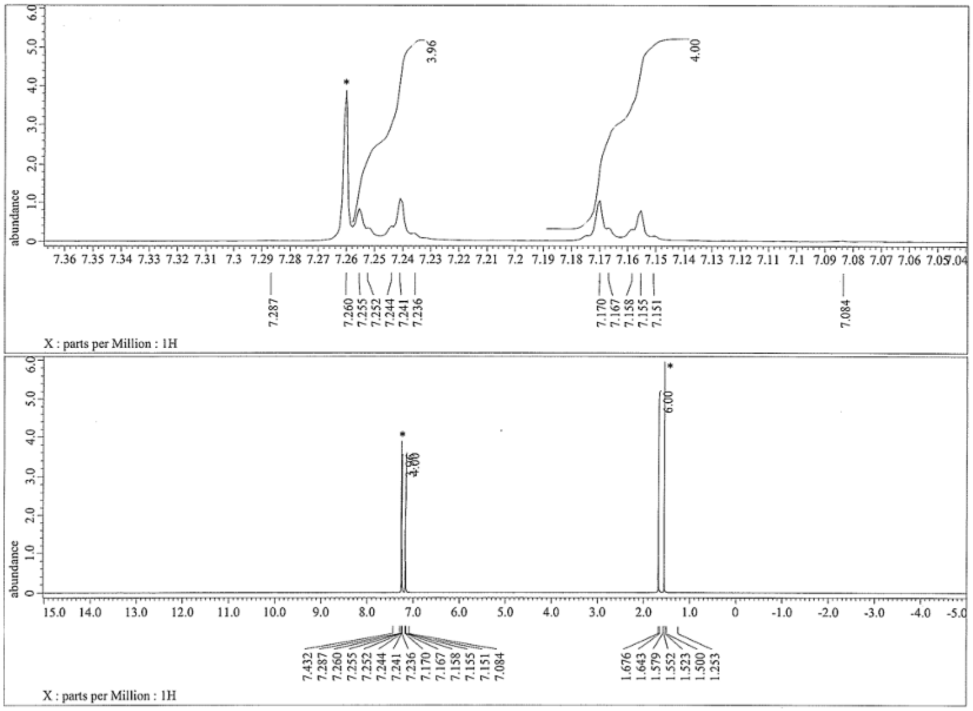

Supplementary Fig. 16. ^{1}H NMR spectrum of **PC0** in $CDCl_3$ at 25 °C. Peaks marked with * indicate residual solvents.

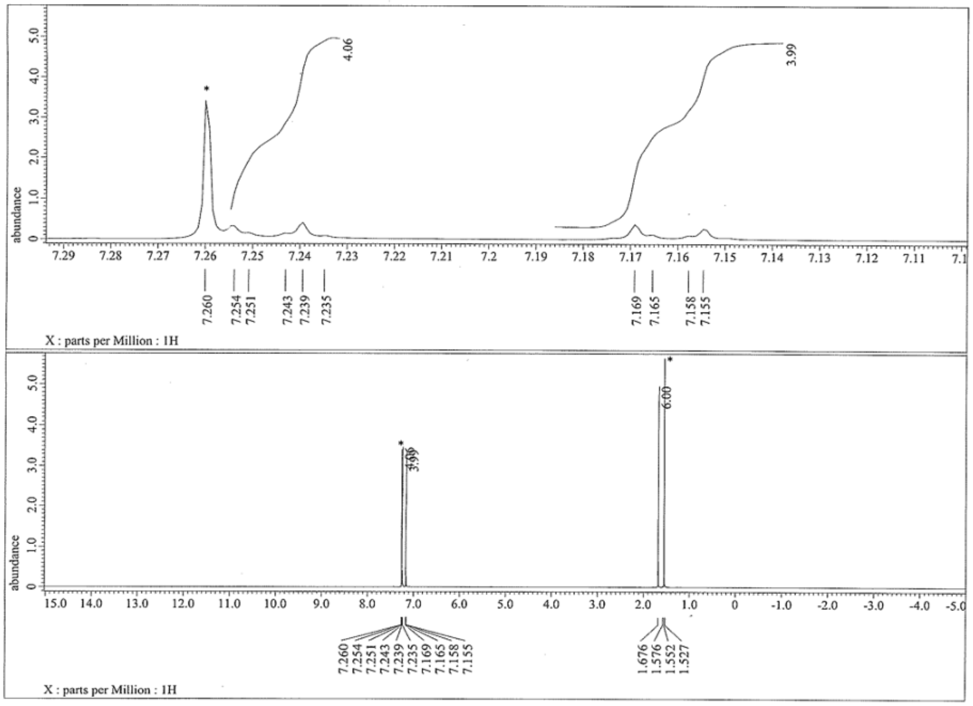

Supplementary Fig. 17. ^{1}H NMR spectrum of **PC1** in $CDCl_3$ at 25 °C. Peaks marked with * indicate residual solvents.

DSC analysis of the linear polycarbonates (PCs)

Differential scanning calorimetry (DSC) measurement of the linear polycarbonates (PCs) was conducted under N_2 atmosphere at a flow speed of 30 mL min^{-1}. Programmed heating and cooling cycles are shown in Fig S18. Fig S19 shows DSC profiles of the 2nd cooling (**E→F**) and heating (**G→H**) cycles. Each sample was placed into an aluminum pan and covered with an aluminum cover, which was well pressed with a designated pressing tool. As a refence sample, a vacant pan was also pressed with the cover.

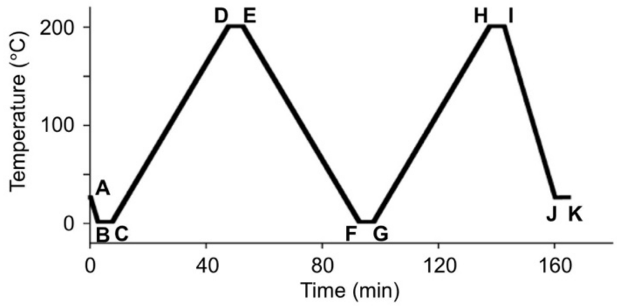

A→B: Cooling from 25 to 0 °C at a rate of 5 °C min^{-1}.
B→C: Hold at 0 °C for 5 min.
C→D: Heating from 0 to 200 °C at a rate of 5 °C min^{-1}.
D→E: Hold at 200 °C for 5 min.
E→F: Cooling from 200 to 0 °C at a rate of 5 °C min^{-1}
F→G: Hold at 0 °C for 5 min.
G→H: Heating from 0 to 200 °C at a rate of 5 °C min^{-1}.
H→I: Hold at 200 °C for 5 min.
I→J: Cooling from 200 to 25 °C at a rate of 10 °C min^{-1}.
J→K: Hold at 25 °C for 5 min.

Supplementary Fig. 18. Programmed heating and cooling cycles in the DSC measurement of the PCs.

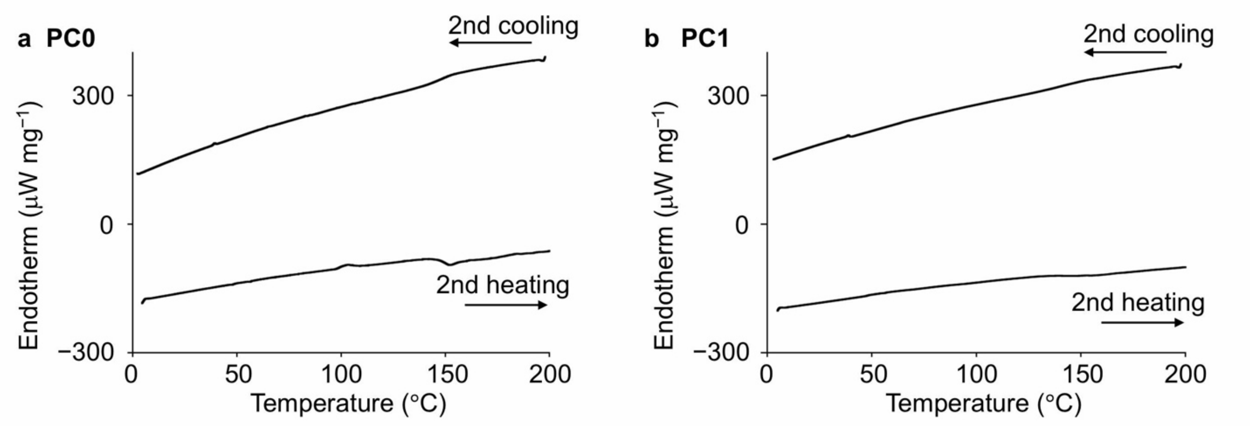

Supplementary Fig. 19. DSC traces of **PC0** and **PC1**. Glass transition temperatures (T_g) were estimated to be 151 °C for **PC0** and 153 °C for **PC1**.

Mechanical properties of the linear polycarbonates (PCs)

Uniaxial tensile tests were carried out on dumbbell-shaped specimens. True strain (ε_{true}) is calculated by $\varepsilon_{true} = \ln(L/L_0)$, where L means crosshead displacement and the initial length L_0 = 30 mm. True stress (σ_{true}) is calculated by $\sigma_{true} = F/A$, where F is the recorded force and A is the cross-sectional area of the deformed specimen, approximated by $A = A_0(L_0/L)$. Toughness was calculated as the integrated area under the true stress–strain curve. Young's modulus was defined as the slope of stress–strain curves at 0–1% true strain. Stretching rate was fixed at 5 mm min^{-1}, corresponding to the strain rate of 2.8 ×10^{-3} s^{-1}.

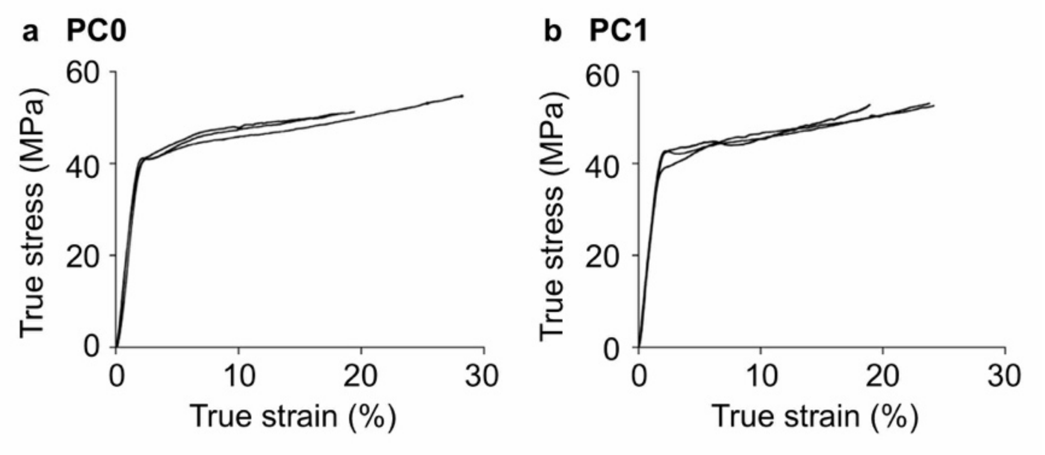

Supplementary Fig. 20. Stress-strain curves of **PC0** and **PC1**.

Supplementary Table 14. Mechanical properties for the PCs.

	True rupture strain (%)	True rupture stress (MPa)	Toughness (MJ m^{-3})	Young's modulus (GPa)
**PC0**	22.0 ± 5.4	52.3 ± 2.1	10.3 ± 2.3	2.20 ± 0.28
**PC1**	22.3 ± 2.9	52.9 ± 0.3	10.0 ± 1.4	2.49 ± 0.10

Photophysical properties of the unstretched polycarbonate (PC)

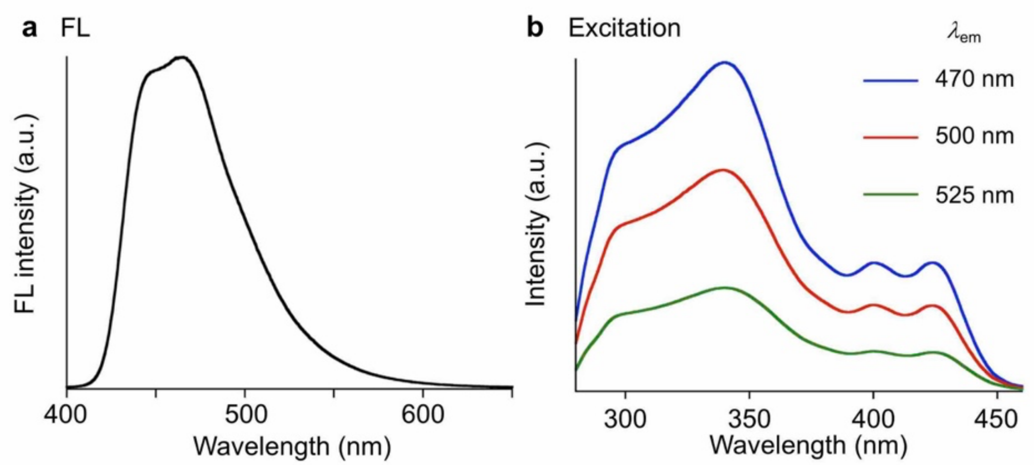

Supplementary Fig. 21. (a) FL spectrum (λ_{ex} = 365 nm) and (b) excitation spectra of the **PC1** film. No excimer emission was observed.

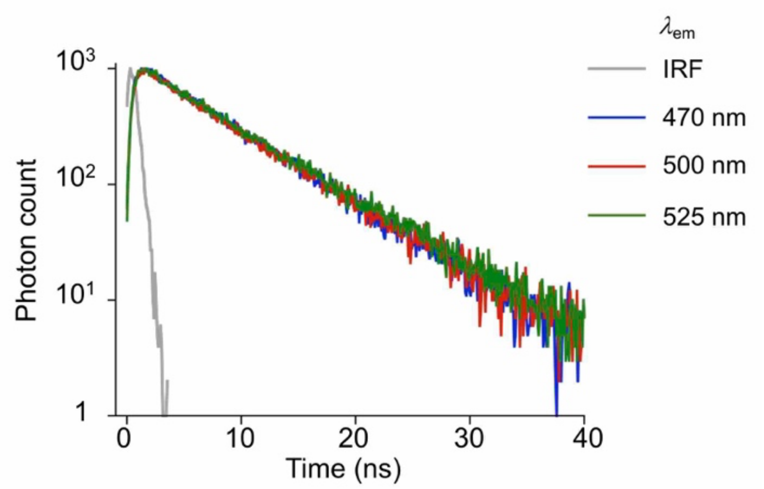

Supplementary Fig. 22. FL decay profiles of **PC1** (λ_{ex} = 365 nm), in which the FL lifetime is almost constant (τ_{FL} = 6.8–7.0 ns) regardless of the monitored FL wavelengths.

Supplementary Table 15. Photophysical constants of **PC1**. λ_{ex} = 365 nm.

Φ_{FL}	τ_{FL} (ns)[a]	k_r (s^{-1})	k_{nr} (s^{-1})
0.30	6.8	4.4×10^7	1.0×10^8

[a] λ_{em} = 470 nm.

Synthesis of the crosslinked polyurethanes (PUs)

Poly(tetrahydrofuran) (PTHF, $M_n \sim 650$, Aldrich) was dried under vacuum (~ 2 torr) for 2 h at 70 °C. After cooling to 25 °C, dimethylformamide (DMF, Super dehydrated grade, Wako Chemicals), a trace of FLAP dopant (**FLAP1** or **FLAP2**), hexamethylene diisocyanate (HDI, TCI chemicals) and triethanolamine (TEA, Wako Chemicals) were added, and the reaction mixture was stirred at 25 °C for 10 min. Then, dibutyltin dilaurate (DBTDL, Aldrich) (30 μL, 50 μmol) in THF (0.5 mL) was added to the reaction and stirred at 25 °C for 2 min. The reaction mixture was poured into a custom-made mold of PTFE (polytetrafluoroethylene), and polymerized under N_2 atmosphere at 25 °C for 48 h. Then, dumbbell-shaped polyurethane (PU) specimens were washed with H_2O and dried under vacuum for 8 h at 70 °C to obtain transparent PU films, **PU1** or **PU2**, with thickness of 1.5 mm and width of 6 mm. **PU0** was obtained by the same procedure without the FLAP dopant.

The average distance between the FLAP molecules doped in PU was estimated to be 20 nm as follows; the weight ratio of FLAP to the polymer was 0.2 mg g^{-1} equal to 0.2 μmol m^{-3} (polymer density: 1.0 g cm^{-3}). Therefore, the average volume occupied by a single FLAP molecule was calculated to be 8.0 $\times 10^{-24}$ m^3. Taking the cube root, the average distance between the FLAP molecules was determined to be 2.0 $\times 10^{-8}$ m^3 (20 nm).

Supplementary Table 16. Components and solvent in the synthesis of **PU0**.

	**PU0** (8.7%TEA)	**PU0** (10%TEA)	**PU0** (13%TEA)
PTHF	5.22 g (8.03 mmol)	4.80 g (7.38 mmol)	4.99 g (7.68 mmol)
HDI	1.73 mL (10.8 mmol)	1.65 mL (10.3 mmol)	1.94 mL (12.1 mmol)
TEA	268 mg (1.80 mmol)	292 mg (1.96 mmol)	439 mg (2.94 mmol)
DMF	14.9 mL	13.9 mL	14.5 mL

Supplementary Table 17. Components and solvent in the synthesis of **PU1**.

	**PU1** (8.7%TEA)	**PU1** (10%TEA)	**PU1** (13%TEA)
**FLAP1**	1.5 mg (1.7 μmol)	1.3 mg (1.5 μmol)	1.6 mg (1.8 μmol)
PTHF	5.14 g (7.91 mmol)	4.67 g (7.18 mmol)	5.03 g (7.74 mmol)
HDI	1.69 mL (10.5 mmol)	1.61 mL (10.0 mmol)	1.96 mL (12.2 mmol)
TEA	262 mg (1.75 mmol)	285 mg (1.91 mmol)	443 mg (2.97 mmol)
DMF	14.7 mL	13.5 mL	14.5 mL
FLAP/polymer[a]	0.21 mg g^{-1}	0.20 mg g^{-1}	0.21 mg g^{-1}

[a] Weight ratio of FLAP to the polymer.

Supplementary Table 18. Components and solvent in the synthesis of **PU2**.

	**PU2** (8.7%TEA)	**PU2** (10%TEA)	**PU2** (13%TEA)
**FLAP2**	1.5 mg (1.5 μmol)	1.5 mg (1.5 μmol)	1.5 mg (1.5 μmol)
PTHF	5.21 g (8.01 mmol)	5.49 g (8.45 mmol)	4.92 g (7.57 mmol)
HDI	1.71 mL (10.6 mmol)	1.89 mL (11.8 mmol)	1.91 mL (11.9 mmol)
TEA	266 mg (1.78 mmol)	335 mg (2.24 mmol)	430 mg (2.88 mmol)
DMF	14.9 mL	15.9 mL	14.2 mL
FLAP/polymer[a]	0.21 mg g^{-1}	0.19 mg g^{-1}	0.20 mg g^{-1}

[a] Weight ratio of FLAP to the polymer.

DSC analysis of the crosslinked polyurethanes (PUs)

Differential scanning calorimetry (DSC) measurement of the crosslinked polyurethane (PU) specimen was conducted under N_2 atmosphere at a flow speed of 30 mL min^{-1}. Programmed heating and cooling cycles are shown in Fig S23. Fig S25 shows DSC profiles of the 2nd cooling (**E→F**) and heating (**G→H**) cycles. Each PU sample was placed into an aluminum pan, covered with an aluminum cover, and then pressed with a designated tool. As a refence sample, a vacant pan was also pressed with the cover.

Spontaneous crystallization behavior was more pronounced in the PU samples with a smaller crosslinking density, as suggested in the values of $\Delta H_{r\to c}$ and $\Delta H_{c\to r}$. Cross-Nicol images in Fig. S24 also suggest that the semicrystalline sample is transformed into the rubbery one upon heating.

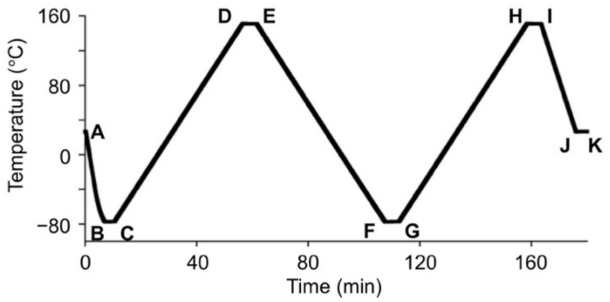

A→B: Cooling from 25 to −80 ° C at a rate of 20 ° C min^{-1}.
B→C: Hold at −80 ° C for 5 min.
C→D: Heating from −80 to 150 ° C at a rate of 5 ° C min^{-1}.
D→E: Hold at 150 °C for 5 min.
E→F: Cooling from 150 to −80 ° C at a rate of 5 ° C min^{-1}
F→G: Hold at −80 ° C for 5 min.
G→H: Heating from −80 to 150 ° C at a rate of 5 ° C min^{-1}.
H→I: Hold at 150 °C for 5 min.
I→J: Cooling from 150 to 25 °C at a rate of 10 °C min^{-1}.
J→K: Hold at 25 °C for 5 min.

Supplementary Fig. 23. Programmed heating and cooling cycles in the DSC measurement of the PUs.

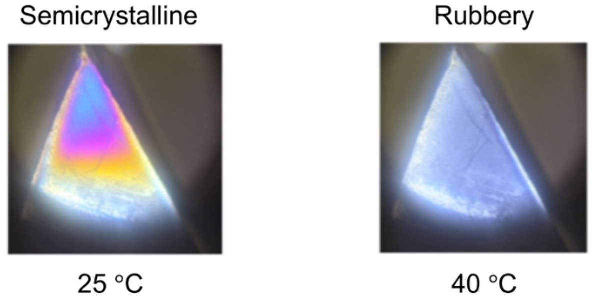

Supplementary Fig. 24. Cross-Nicol images of **PU0** (8.7%TEA) at 20 and 40 °C.

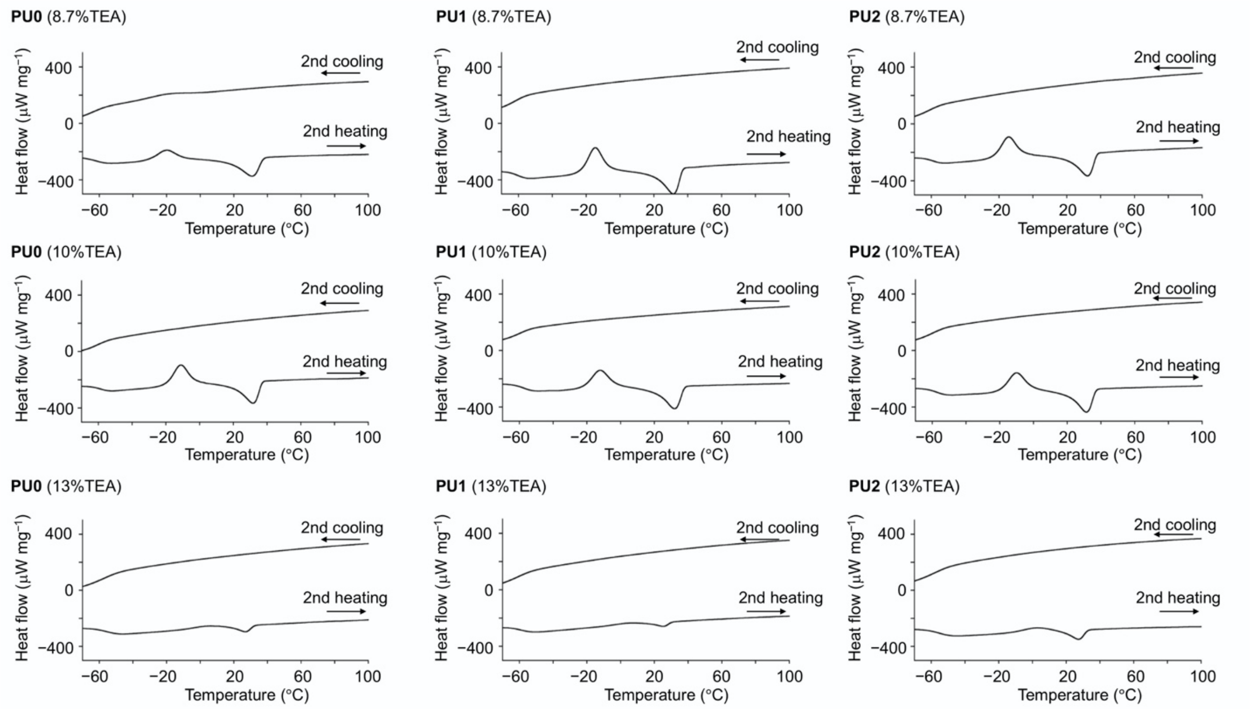

Supplementary Fig. 25. DSC traces of the crosslinked PUs.

Supplementary Table 19. DSC parameters of the PU films with each crosslinking density (%TEA).

	weight (mg)	T_g [a] (°C)	$T_{r→c}$ [b] (°C)	$ΔH_{r→c}$ [c] (J g^{-1})	$T_{c→r}$ [d] (°C)	$ΔH_{c→r}$ [e] (J g^{-1})
**PU0** (8.7%TEA)	7.50	−60	−19	−16	31	21
**PU1** (8.7%TEA)	6.42	−60	−14	−24	31	24
**PU2** (8.7%TEA)	6.25	−58	−14	−24	32	22
**PU0** (10%TEA)	6.84	−58	−11	−23	32	20
**PU1** (10%TEA)	9.99	−56	−12	−24	32	22
**PU2** (10%TEA)	7.33	−57	−10	−26	31	19
**PU0** (13%TEA)	6.67	−56	5	−3.7	27	5.7
**PU1** (13%TEA)	6.01	−59	8	−2.8	25	4.0
**PU2** (13%TEA)	5.88	−56	3	−3.9	27	6.5

[a] Glass transition temperature determined by the 2nd heating profile.
[b] Rubbery-to-semicrystalline transition temperature determined by the 2nd heating profile.
[c] Enthalpy change in the rubbery-to-semicrystalline transition determined by the DSC signal area.
[d] Semicrystalline-to-rubbery transition temperature determined by the 2nd heating profile.
[e] Enthalpy change in the semicrystalline-to-rubbery transition determined by the DSC signal area.

Rheological analysis of the crosslinked polyurethanes (PUs)

Dynamic viscoelasticity was measured for the rubbery PUs. After heating to 100 °C, the storage modulus G' and loss modulus G'' were measured up to 0 °C at a cooling rate of 5 °C min^{-1} (frequency: 1 Hz, a 10-mm parallel plate). As the temperature dropped, both G' and G'' increased gradually. The increments were small, and the G' value stayed in the range of 10^5–10^6 Pa, indicating much softer properties of the crosslinked PUs than the linear PCs prepared above.

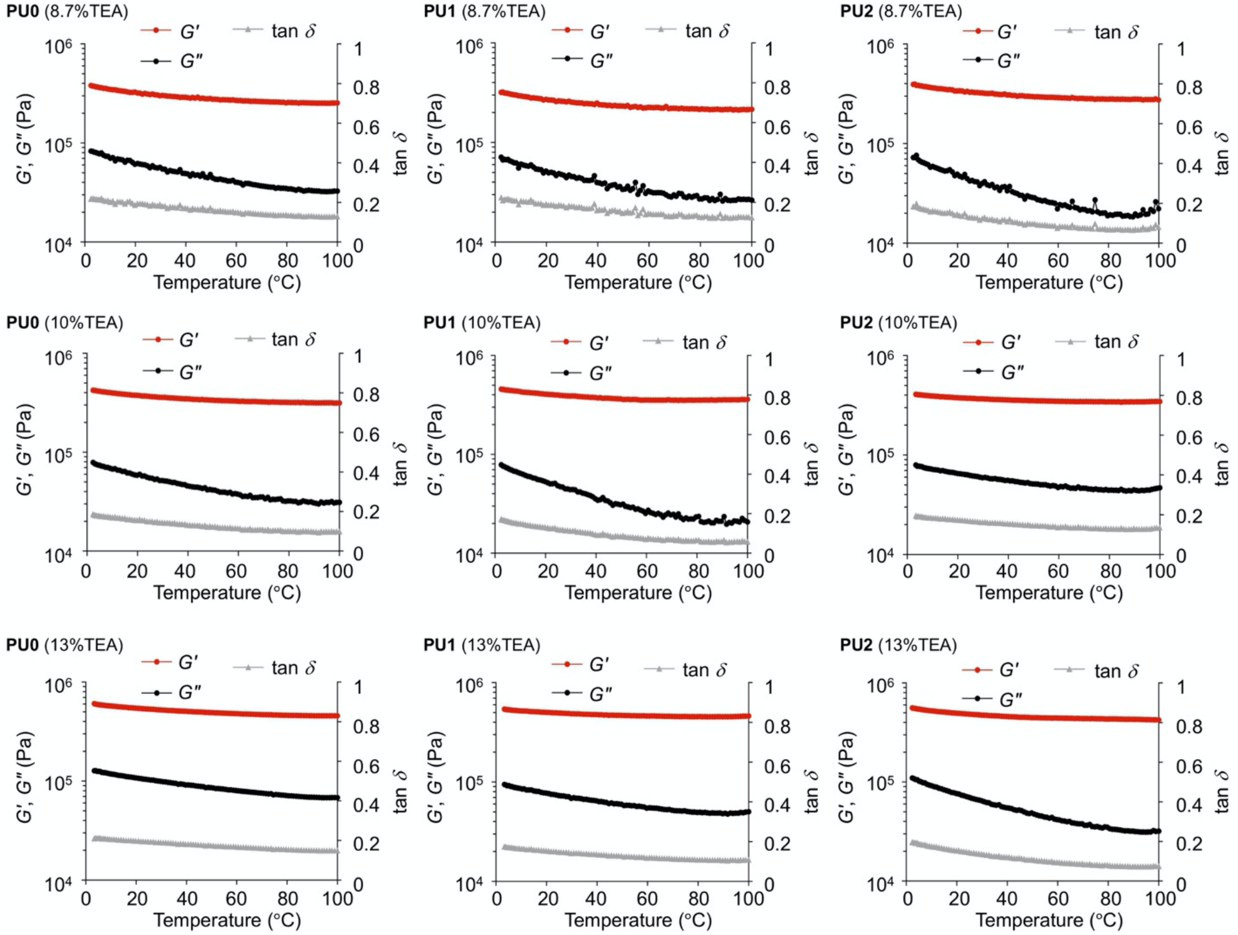

Supplementary Fig. 26. Dynamic viscoelasticity of the crosslinked PUs.

Supplementary Table 20. Dynamic viscoelasticity of the PUs at 25 °C.

	G' (Pa) [a]	G'' (Pa) [b]	tan δ [c]
**PU0** (8.7%TEA)	3.1×10^5	5.9×10^4	0.19
**PU1** (8.7%TEA)	2.6×10^5	4.8×10^4	0.18
**PU2** (8.7%TEA)	3.3×10^5	4.2×10^4	0.13
**PU0** (10%TEA)	3.7×10^5	5.5×10^4	0.15
**PU1** (10%TEA)	3.9×10^5	4.8×10^4	0.12
**PU2** (10%TEA)	3.7×10^5	6.2×10^4	0.17
**PU0** (13%TEA)	5.3×10^5	1.0×10^5	0.19
**PU1** (13%TEA)	4.9×10^5	7.3×10^4	0.15
**PU2** (13%TEA)	4.8×10^5	7.0×10^4	0.15

[a] Storage modulus at 25 °C. [b] Loss modulus at 25 °C. [c] Loss tangent obtained from G''/G' at 25 °C.

With higher crosslinking density (%TEA), the G' values increased significantly. The FLAP dopants in **PU1** and **PU2** did not affect the viscoelasticity of the bulk PU sample.

Mechanical properties of the rubbery polyurethanes (PUs)

Uniaxial tensile tests were carried out on dumbbell-shaped specimens. Rubbery samples were prepared by heating at 60 °C for 1 min with a blow dryer. Nominal stress, σ_N, is defined as f/S_0, where applied force f is divided by the initial cross-sectional area S_0 (typically 1.5-mm thickness × 6-mm width).

$$\sigma_N = \frac{f}{S_0}$$

Nominal strain, ε_N, is defined as $\Delta L/L_0$, where sample gauge length (ΔL) is divided by the initial length (L_0 = 10 mm).

$$\varepsilon_N = \frac{\Delta L}{L_0}$$

Toughness was calculated as the integrated area under the stress–strain curve.
Young's modulus is defined as the slope of stress–strain curves at 0–10% strain. Tensile velocity was fixed at 100 mm min^{-1}, corresponding to strain rate of 0.17 s^{-1}.

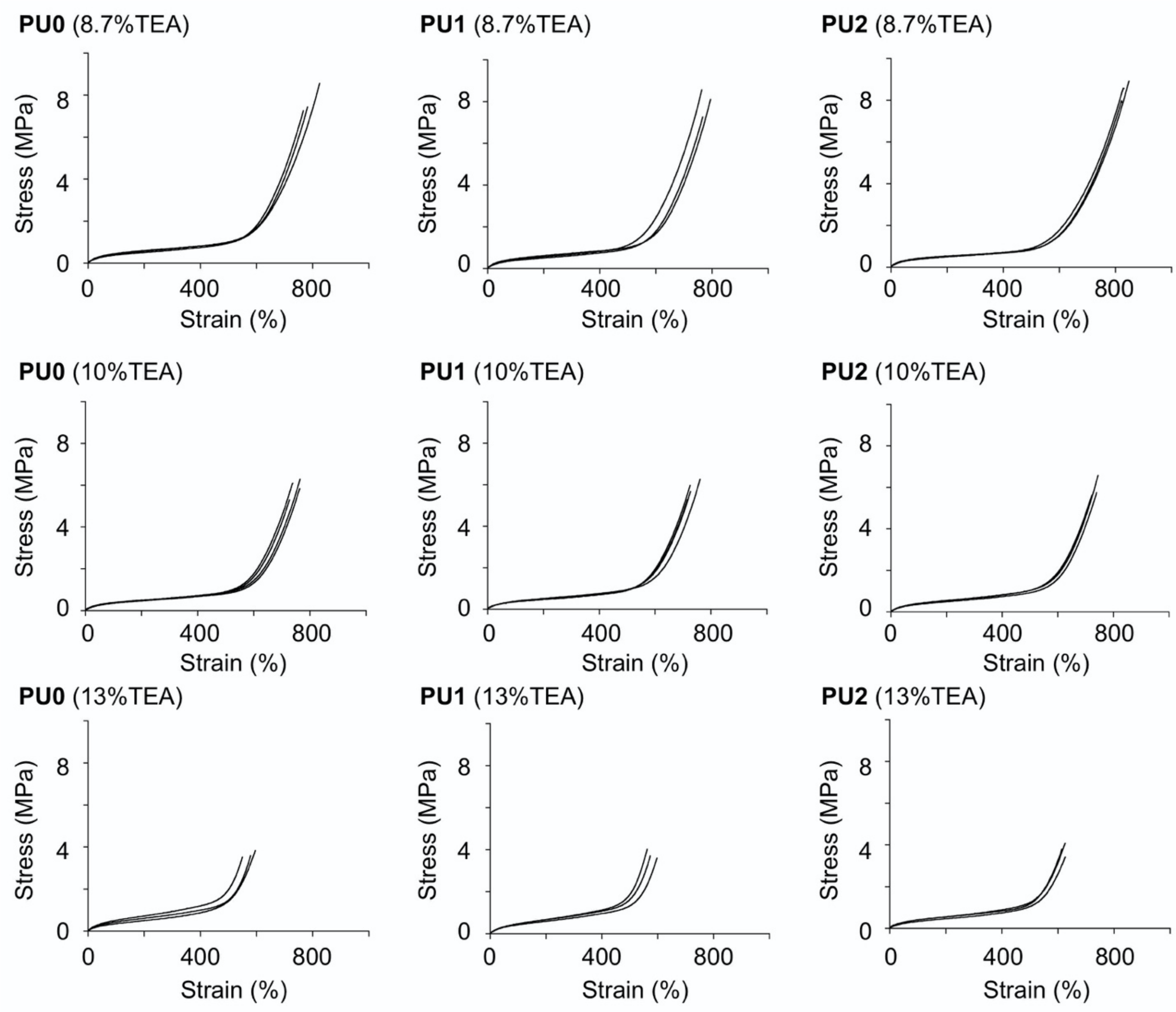

Supplementary Fig. 27. Stress-strain curves of the rubbery PUs.

Supplementary Table 21. Mechanical properties of the rubbery PUs in the uniaxial tensile testing.

	Rupture strain (%)	Rupture stress (MPa)	Toughness (MJ m^{-3})	Young's modulus (MPa)
**PU0** (8.7%TEA)	801 ± 44	7.2 ± 1.9	12.0 ± 3.3	1.2 ± 0.1
**PU1** (8.7%TEA)	791 ± 30	7.8 ± 0.7	12.3 ± 1.2	1.2 ± 0.2
**PU2** (8.7%TEA)	824 ± 8	8.2 ± 0.3	12.6 ± 0.5	1.1 ± 0.1
**PU0** (10%TEA)	749 ± 19	5.9 ± 0.4	8.7 ± 0.7	1.1 ± 0.1
**PU1** (10%TEA)	732 ± 20	5.8 ± 0.4	8.6 ± 0.7	1.0 ± 0.1
**PU2** (10%TEA)	733 ± 11	6.0 ± 0.4	9.2 ± 1.0	1.1 ± 0.1
**PU0** (13%TEA)	575 ± 24	3.6 ± 0.2	5.4 ± 0.2	1.1 ± 0.1
**PU1** (13%TEA)	577 ± 18	3.6 ± 0.4	5.6 ± 0.1	1.1 ± 0.1
**PU2** (13%TEA)	616 ± 12	4.0 ± 0.5	5.8 ± 0.6	1.1 ± 0.2

Values of average ± standard deviation of 3–4 specimen were shown.

Photophysical properties of the unstretched polyurethanes (PUs)

FL spectra in Supplementary Figs. 28–33 were recorded on a JASCO Spectrofluorometer FP-8500. The synthesized polyurethane (PU) films become semicrystalline when stored at room temperature (25 °C) for more than 2 days. Therefore, the rubbery samples were prepared by heating at 60 °C for 1 min with a blow dryer. While mechanical properties were largely dependent on the crystallinity (Supplementary Fig. 39), FL spectra, FL quantum yields, and FL lifetimes of the unstretched PU films showed small differences (Supplementary Table 22, Supplementary Figs. 28–33). No excimer formation was observed in these conditions.

Supplementary Table 22. Photophysical constants of semicrystalline and rubbery PU films with each crosslinking density (%TEA). λ_{ex} = 365 nm.

	Φ_{FL}	τ_{FL} (ns)[a]	k_r (s^{-1})	k_{nr} (s^{-1})
semicrystalline **PU1** (8.7%TEA)	0.30	6.8	4.4×10^7	1.0×10^8
rubbery **PU1** (8.7%TEA)	0.26	6.9	3.8×10^7	1.1×10^8
semicrystalline **PU1** (10%TEA)	0.30	6.7	4.4×10^7	1.0×10^8
rubbery **PU1** (10%TEA)	0.28	6.9	4.0×10^7	1.0×10^8
semicrystalline **PU1** (13%TEA)	0.33	6.9	4.8×10^7	1.0×10^8
rubbery **PU1** (13%TEA)	0.31	7.1	4.3×10^7	1.0×10^8
semicrystalline **PU2** (8.7%TEA)	0.35	6.8	5.1×10^7	1.0×10^8
rubbery **PU2** (8.7%TEA)	0.37	7.0	3.8×10^7	0.9×10^8
semicrystalline **PU2** (10%TEA)	0.35	6.7	5.2×10^7	1.0×10^8
rubbery **PU2** (10%TEA)	0.35	7.1	5.0×10^7	0.9×10^8
semicrystalline **PU2** (13%TEA)	0.33	7.0	4.6×10^7	1.0×10^8
rubbery **PU2** (13%TEA)	0.33	6.9	4.8×10^7	1.0×10^8

[a] λ_{em} = 470 nm.

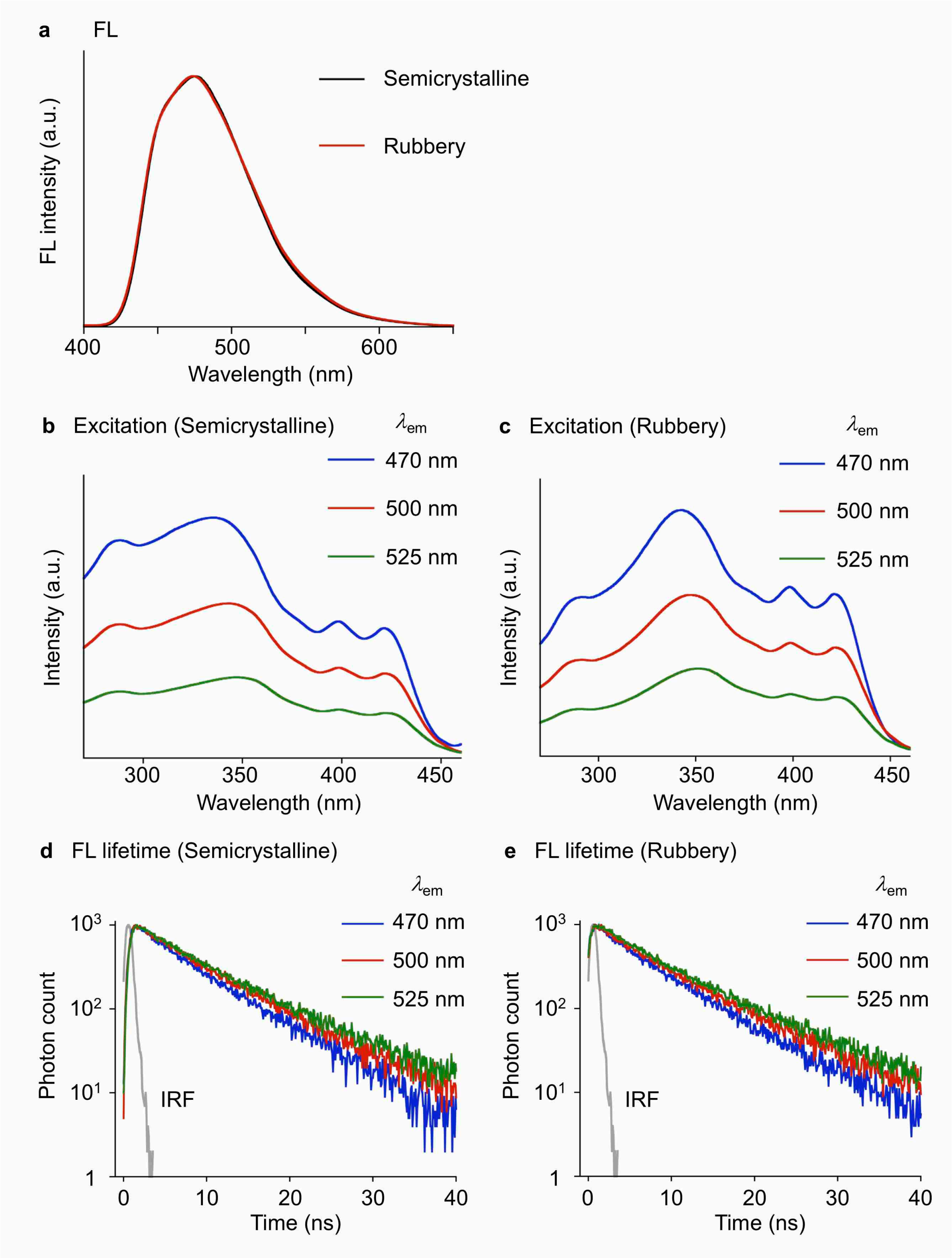

Supplementary Fig. 28. Photophysical properties of semicrystalline and rubbery **PU1** films (8.7%TEA). (a) FL spectra (λ_{ex} = 365 nm), (b, c) excitation spectra, and (d, e) FL decay profiles.

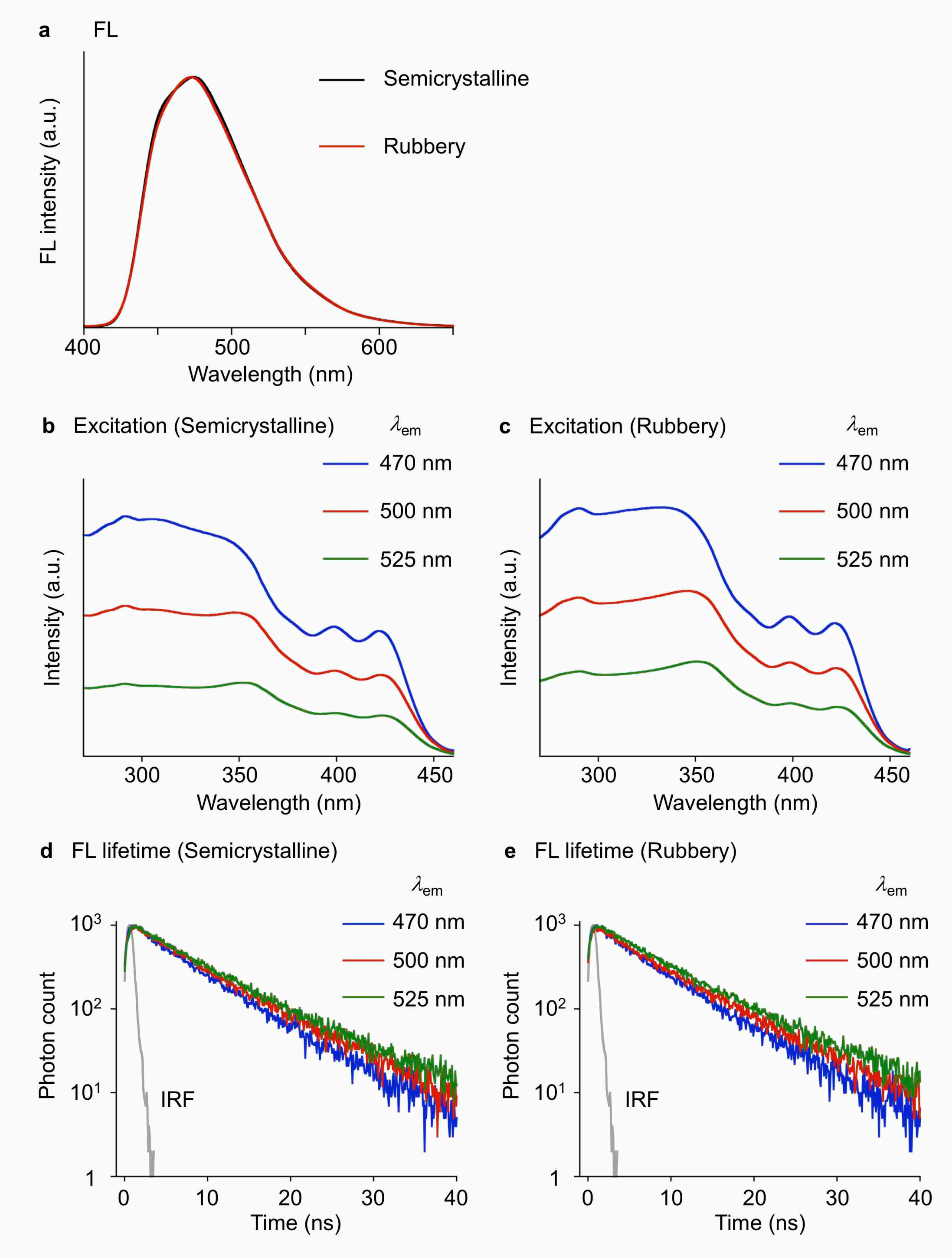

Supplementary Fig. 29. Photophysical properties of semicrystalline and rubbery **PU1** films (10%TEA). (a) FL spectra (λ_{ex} = 365 nm), (b, c) excitation spectra, and (d, e) FL decay profiles.

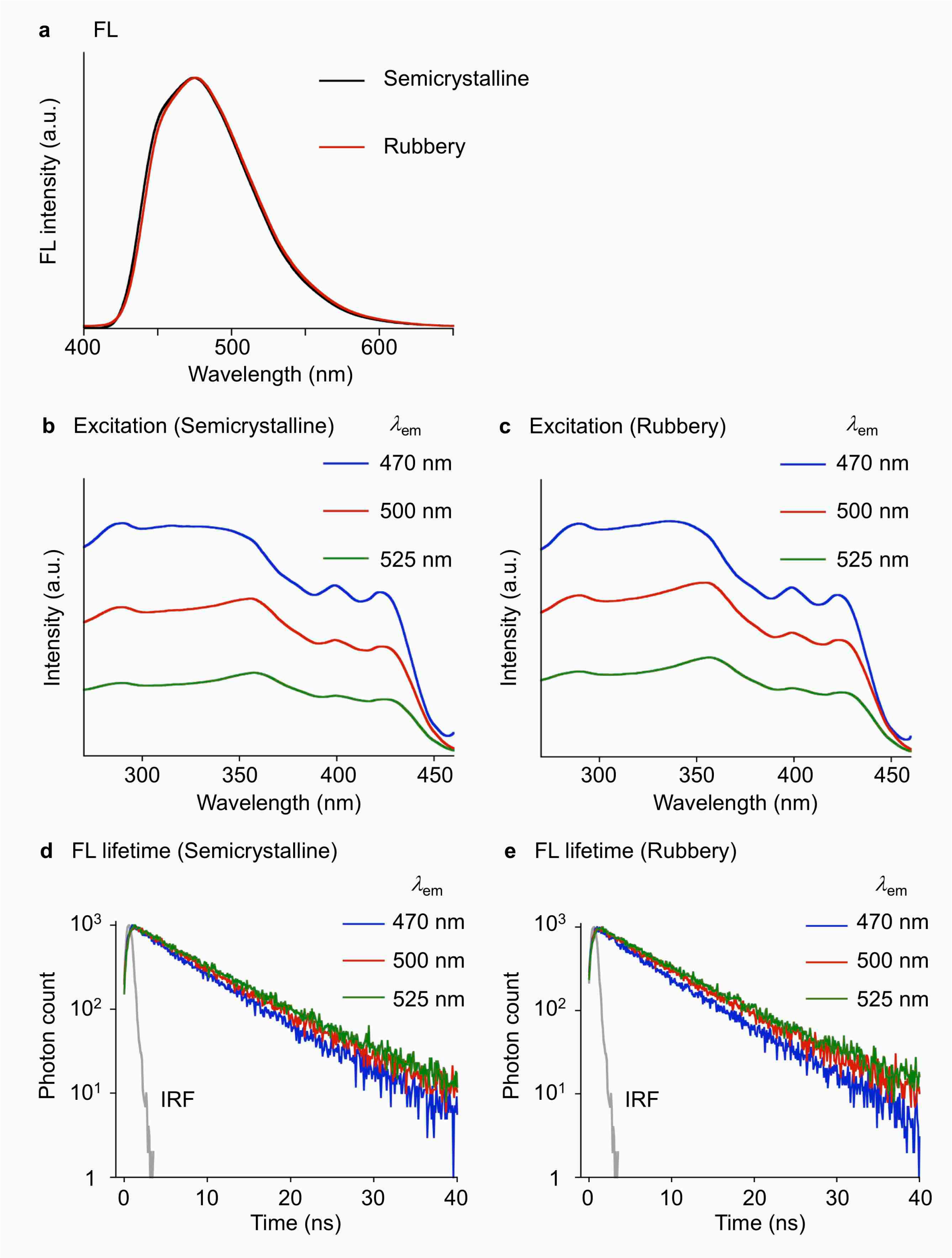

Supplementary Fig. 30. Photophysical properties of semicrystalline and rubbery **PU1** films (13%TEA). (a) FL spectra (λ_{ex} = 365 nm), (b, c) excitation spectra, and (d, e) FL decay profiles.

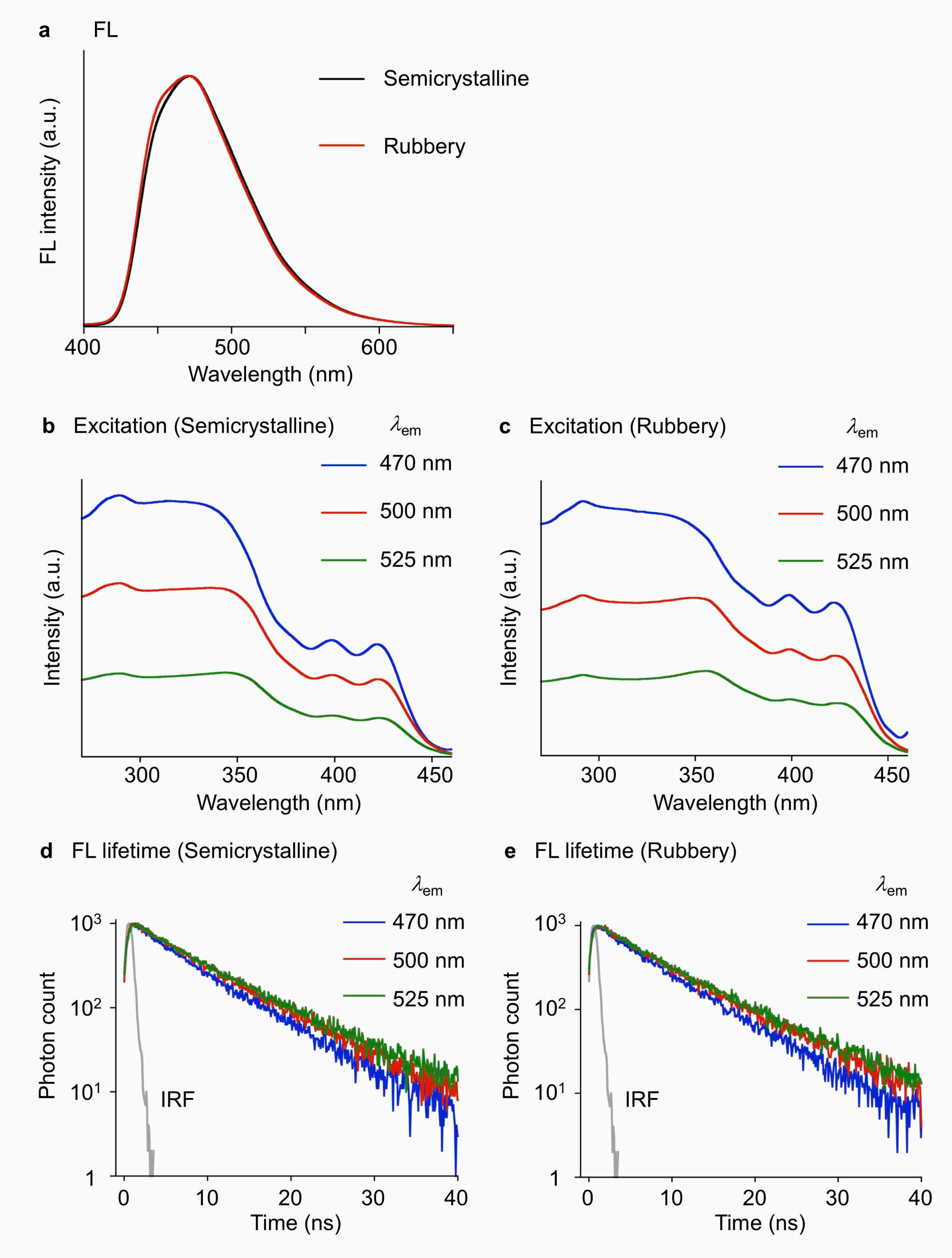

Supplementary Fig. 31. Photophysical properties of semicrystalline and rubbery **PU2** films (8.7%TEA). (a) FL spectra (λ_{ex} = 365 nm), (b, c) excitation spectra, and (d, e) FL decay profiles.

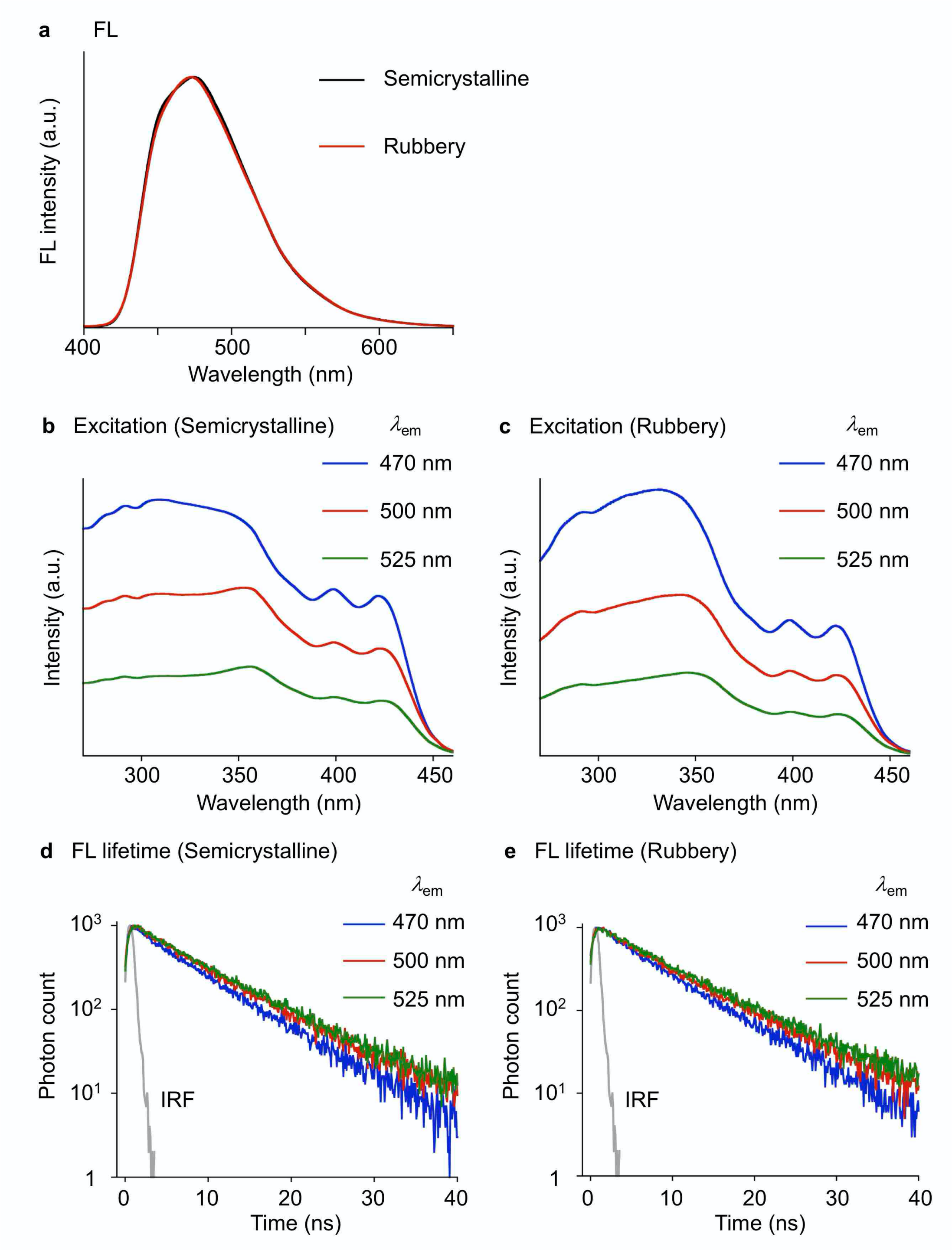

Supplementary Fig. 32. Photophysical properties of semicrystalline and rubbery **PU2** films (10%TEA). (a) FL spectra (λ_{ex} = 365 nm), (b, c) excitation spectra, and (d, e) FL decay profiles.

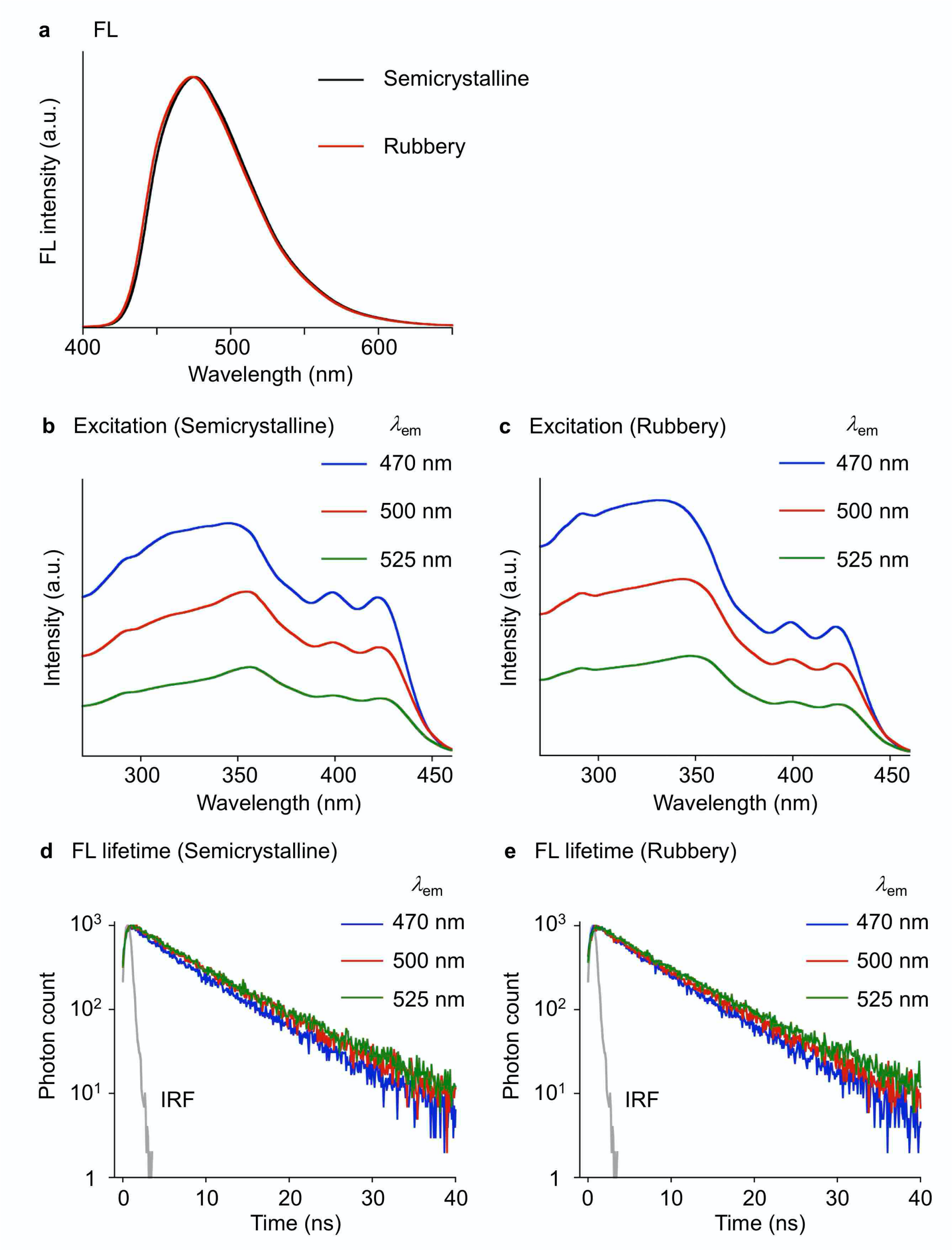

Supplementary Fig. 33. Photophysical properties of semicrystalline and rubbery **PU2** films (13%TEA). (a) FL spectra (λ_{ex} = 365 nm), (b, c) excitation spectra, and (d, e) FL decay profiles.

Real-time monitoring of the FL and absorption spectra of the stretched PUs

During the tensile testing, time-dependent FL and absorption spectra were monitored using a multi-channel photodetector (Otsuka Electronics, MCPD-6800) equipped with an optical fiber. The FL spectra were recorded with 0.8-s exposure time and 2 accumulations (at 1.6-s intervals), in which a 365-nm LED was used for excitation. Absorption spectra were recorded on the same photodetecting system with a 1.6-s exposure time and 2 accumulations (at 3.2-s intervals). Before the tensile testing, reference absorption spectrum $I_0(\lambda)$ was measured with a 100-W tungsten light source fixed at a constant distance from the optical fiber head. Then, a PU specimen was set (and stretched) so that the specimen crossed the optical path to acquire absorption spectrum $I(\lambda)$. Absorbance A at each wavelength was obtained from $I_0(\lambda)$ and $I(\lambda)$ as below.

$$A = -\log\left(\frac{I}{I_0}\right)$$

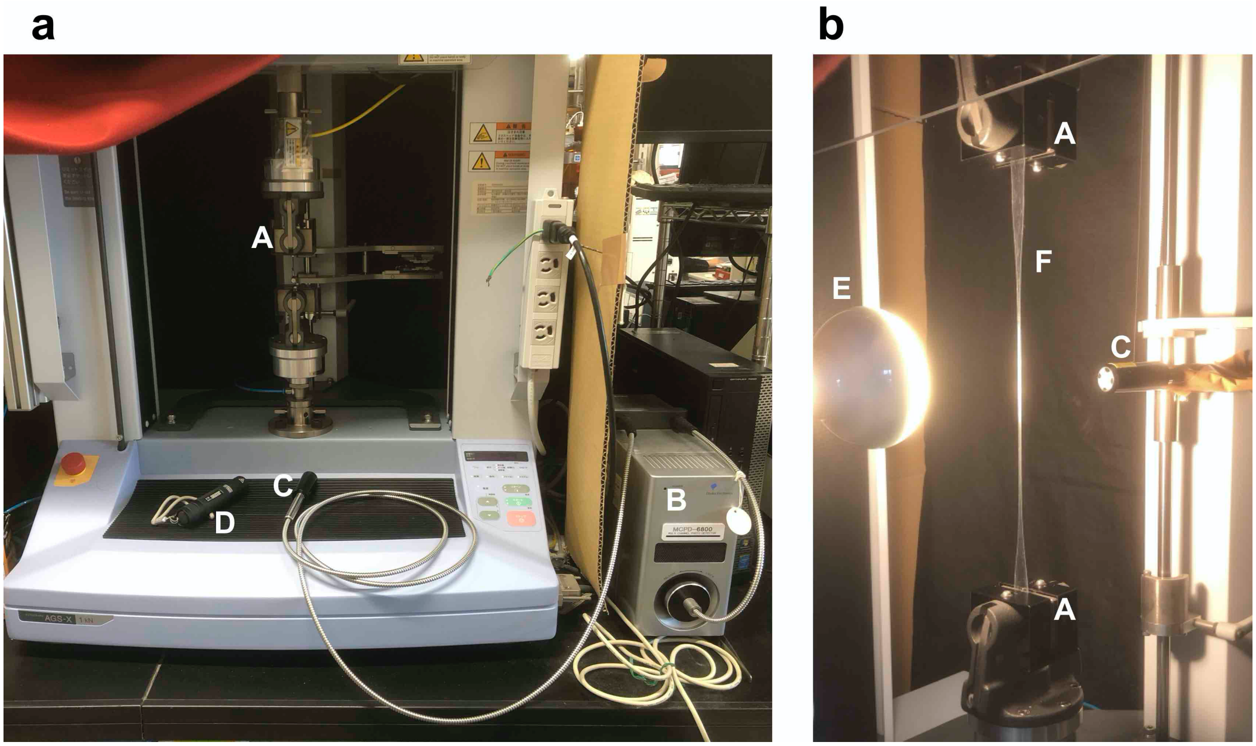

Supplementary Fig. 34. Experimental setup for the real-time collection of (a) FL spectra and (b) absorption spectra of the stretched PU films.

A: Upper and lower grips of a tensile testing machine to hold a specimen.
B: Multi-channel photodetector.
C: Optical fiber head connected to **B**.
D: 365-nm LED (NICHIA, JAXMAN U1).
E: 100-W tungsten light (METRO).
F: PU specimen.

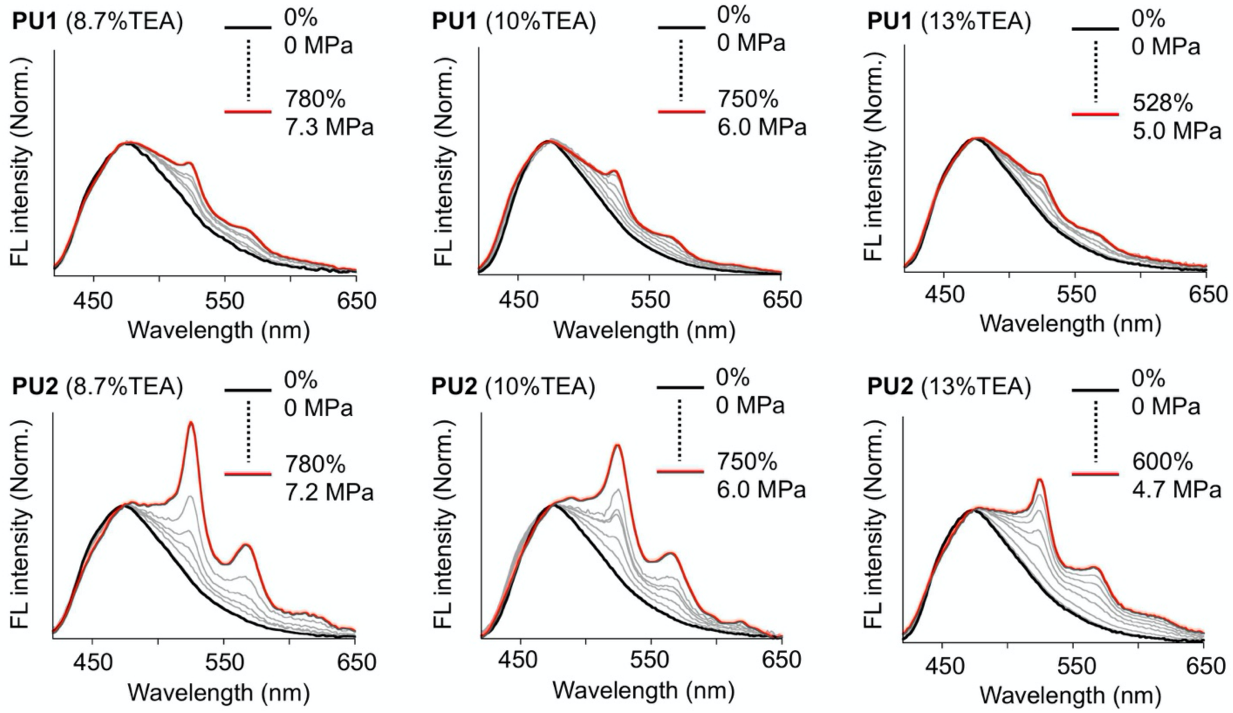

Supplementary Fig. 35. FL spectral changes of the rubbery PUs. Normalized at 474 nm. λ_{ex} = 365 nm.

Loading–unloading cycle testing

To confirm the rapid reversibility of the stress-induced FL response, the loading and unloading cycle testing of **PU2** (10%TEA) was performed with ratiometric FL analysis (Fig. 4F in the main text). Supplementary Fig. 36 shows mechanical profiles in the cycle test.

Start → **A**: Loading up to 300% strain (purple line).
A → **B**: Unloading up to stress of 0.1 MPa (blue line).
B → **C**: Loading up to 600% strain (green line).
C → **D**: Unloading up to stress of 0.1 MPa (orange line).
D → **E**: Loading up to rupture (red line).

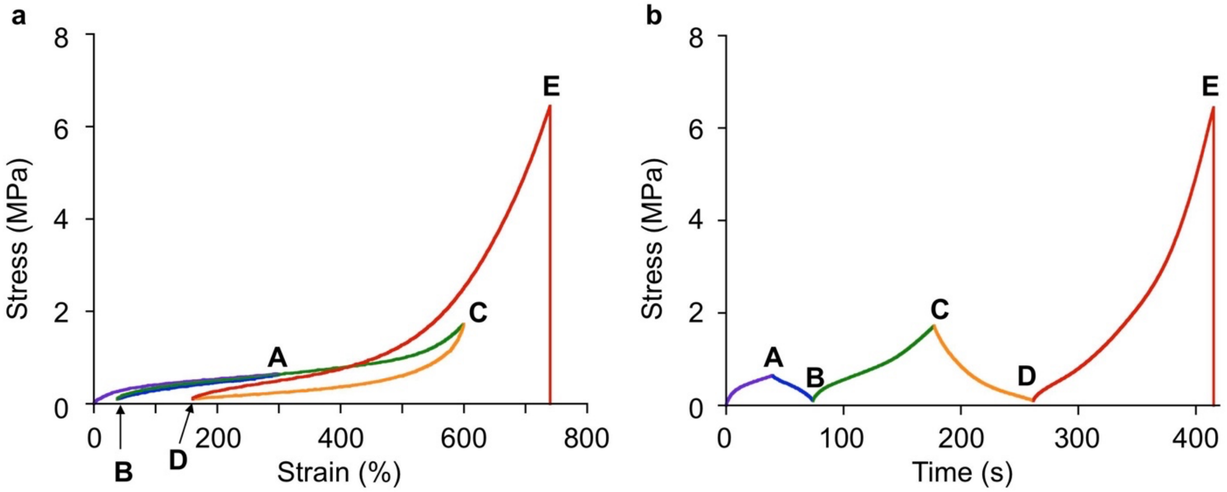

Supplementary Fig. 36. Stress profile as a function of (a) strain and (b) time for the cycle testing.

Estimation of the stressed FLAP probe (%)

Percentage of the stressed FLAP probe over the FL switching threshold was estimated from the ratiometric FL analysis of the stretched PU films (Fig. 5 in the main text).

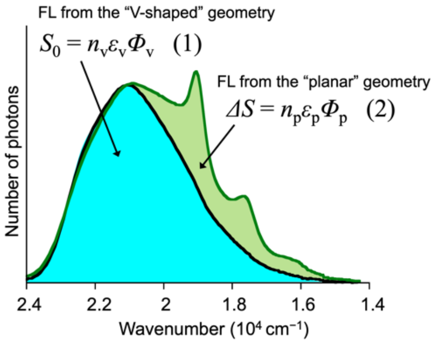

S_0: area of the FL spectrum for unstretched specimen (blue)
ΔS: increase in the area of the FL spectrum for stretched specimen (green)
n_v: number of the relaxed V-shaped molecules
n_p: number of the stressed planar molecules
ε_v: molar absorption coefficient at 365 nm for the relaxed V-shaped molecules
ε_p: molar absorption coefficient at 365 nm for the stressed planar molecules
Φ_v: FL quantum yield for the relaxed V-shaped molecules
Φ_p: FL quantum yield for the stressed planar molecules

Supplementary Fig. 37. Optical parameters for the estimation.

For the estimation, it was assumed that the FL emission was observed mainly from two types of independent species bearing the bent and planar geometries. Considering (1) and (2) in Supplementary Fig. 37, the proportion of the stressed planar FLAP molecules can be expressed as follows,

$$\frac{n_p}{n_p+n_v} = \frac{1}{1+\frac{n_v}{n_p}} = \frac{1}{1+\frac{\varepsilon_p \Phi_p}{\varepsilon_v \Phi_v} \bullet \frac{S_0}{\Delta S}} \qquad (3)$$

Here, molar absorption coefficients were roughly estimated as ε_p = 140000 M^{-1} cm^{-1} for the planar geometry and ε_v = 70000 M^{-1} cm^{-1} for the bent geometry, considering TD-DFT calculation results at the TD PBE0/6-31+G(d) level of theory. The FL quantum yields of the independent planar and bent species were assumed to be comparable ($\Phi_v \approx \Phi_p$), because Φ_v was determined to be about 0.3 from the FL analysis of unstretched PU polymers with the FLAP dopant (Supplementary Table 22), and Φ_p was referenced from the FL quantum yield (about 0.3) of the parent FLAP compounds in solution (Supplementary Table 1).

Control experiments: Physical doping of FLAP into the polyurethane (PU)

PU3 was synthesized in the same procedure as **PU1** (10%TEA), in which **FLAP1** was replaced by **FLAP3**[8]. **FLAP3** is not covalently connected to the polymer chain network of **PU3**, but simply dispersed inside **PU3**. Without extension, mechanical and photophysical properties of the **PU3** film are almost the same as those of **PU1** and **PU2**. On the other hand, no characteristic FL response was observed under the tensile testing, clearly indicating that the conformational planarization of the FLAP dopant cannot be induced without covalent connection between the FLAP molecule and the polymer chains.

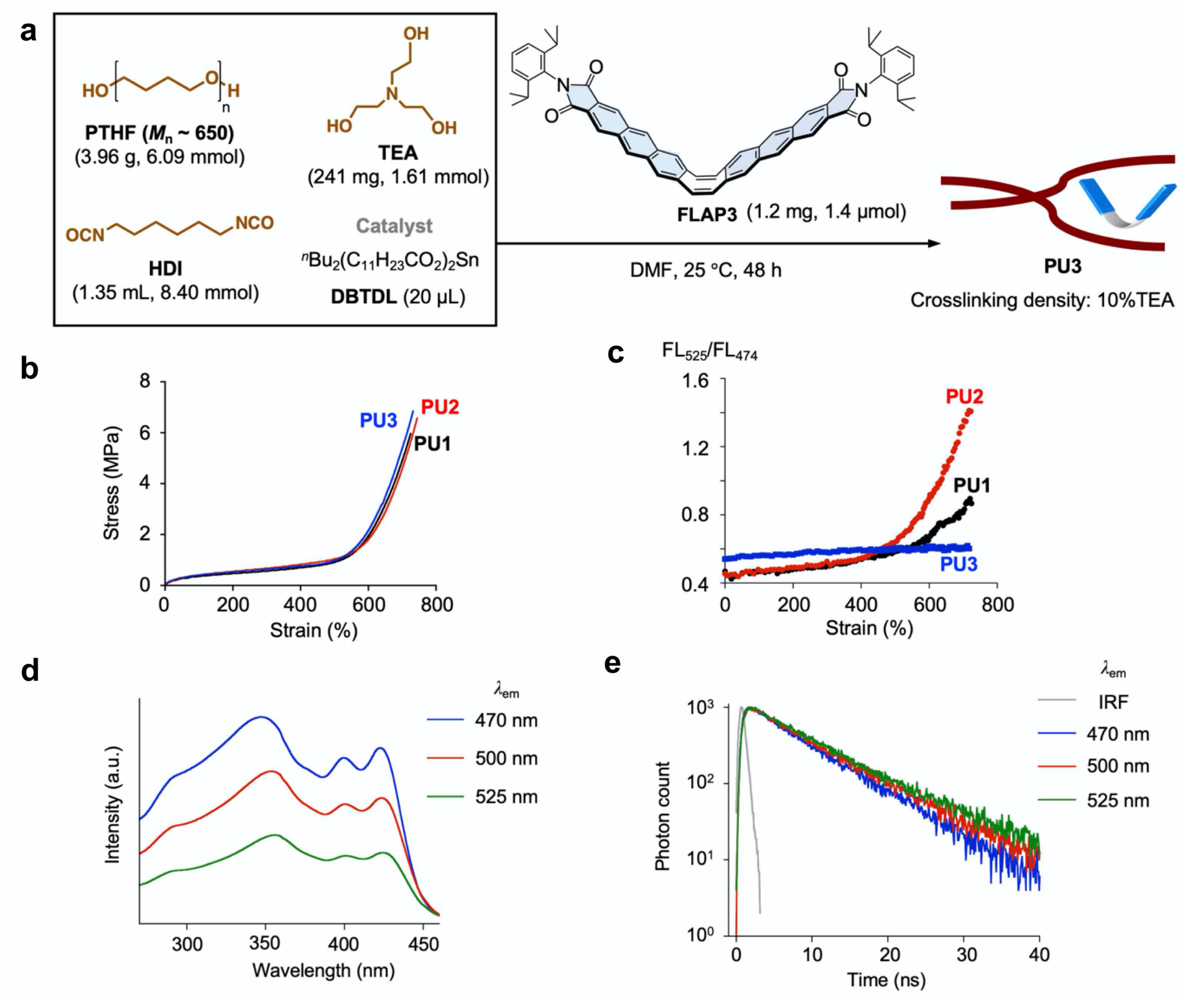

Supplementary Fig. 38. (a) Synthesis of **PU3** (10%TEA). (b) Stress-strain curves of **PU1**–**PU3** (10%TEA). Strain rate: 0.17 s^{-1}. (c) FL ratiometric analysis of the stretched **PU1**–**PU3** films. (d) Excitation spectra of the unstretched **PU3** film. (e) FL lifetime profiles of the unstretched **PU3** film.

Supplementary Table 23. Photophysical constants of the rubbery **PU3** films. λ_{ex} = 365 nm.

Φ_{FL}	τ_{FL} (ns)[a]	k_r (s^{-1})	k_{nr} (s^{-1})
0.30	7.1	4.2×10^7	1.0×10^8

[a]λ_{em} = 470 nm. These values were comparable those of **PU1** and **PU2** in Supplementary Table 22.

Semicrystalline polyurethanes (PUs)

Mechanical and photophysical properties of the semicrystalline PU samples, stored at room temperature (25 °C) for more than 2 days, were evaluated in the same protocols as the rubbery PU samples. Young's modulus is more than 10 times higher than that of the rubbery PU (Supplementary Table 24). Accordingly, yield point at the early stage of the strain became more pronounced for the semicrystalline samples (Supplementary Fig. 39), followed by typical necking behavior (Supplementary Fig. 40). Since the necking occurs in a specific position, acquired FL spectra depend on the UV-irradiated area. Rupture strain significantly decreased in the sample with the higher crosslinking density (Supplementary Table 24).

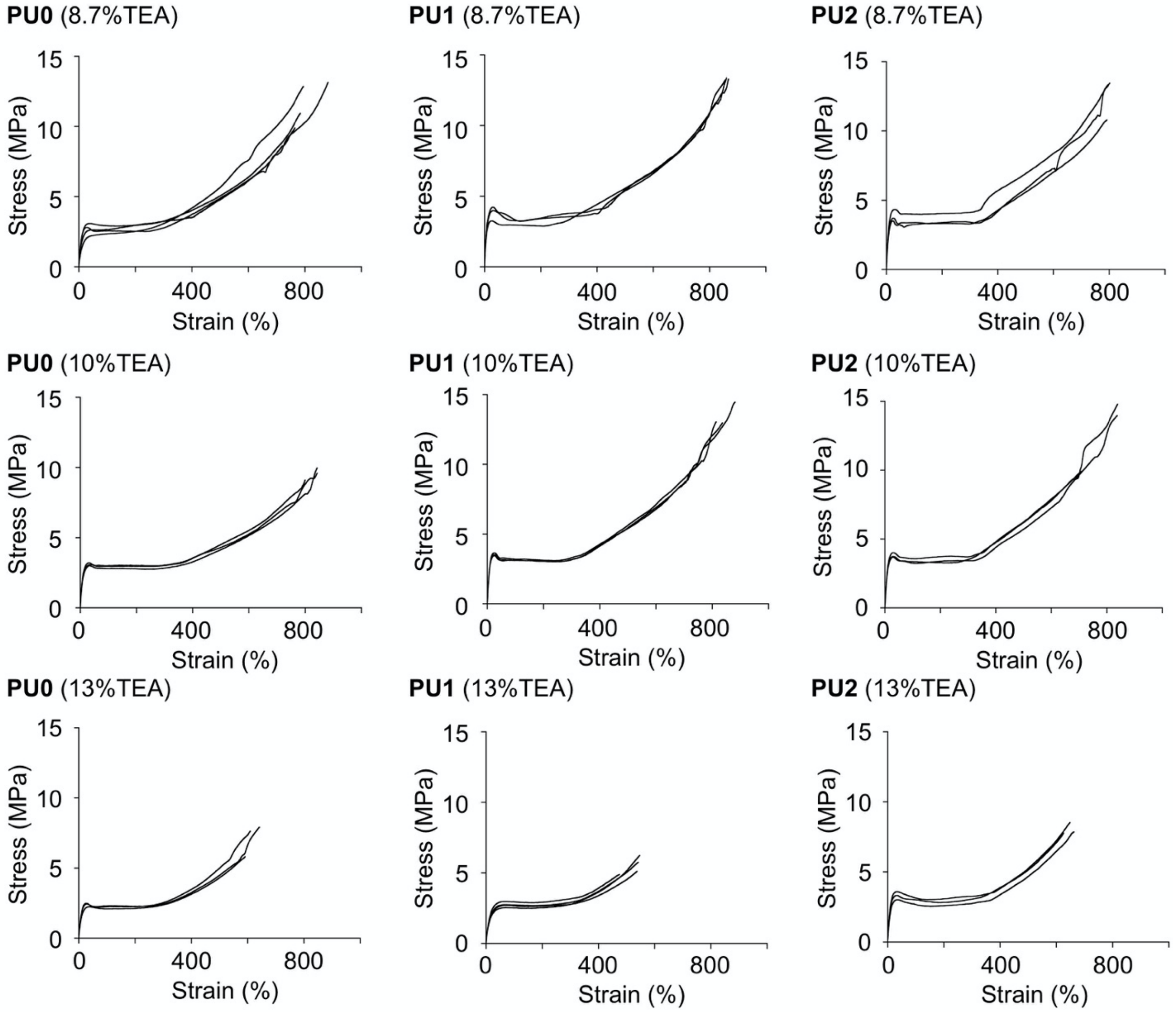

Supplementary Fig. 39. Stress-strain curves of the semicrystalline PUs.

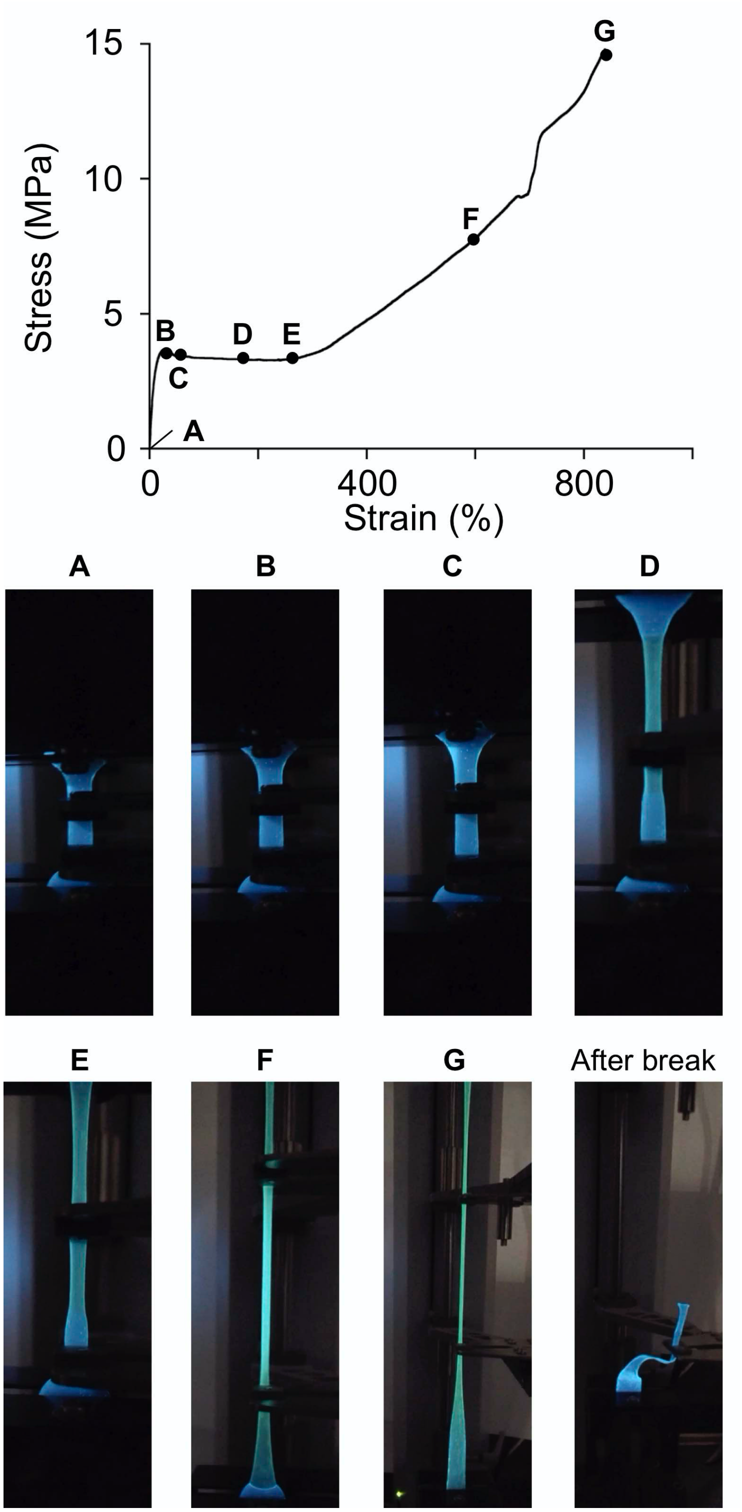

Supplementary Fig. 40. A typical example of the stress-strain curve and the corresponding photographs of the stretched fluorescent specimen, in which the semicrystalline **PU2** (10%TEA) was applied to the tensile testing.

Supplementary Table 24. Mechanical properties of the semicrystalline PUs in the uniaxial tensile testing.

	Rupture strain (%)	Rupture stress (MPa)	Toughness (MJ m^{-3})	Young's modulus (MPa)
**PU0** (8.7%TEA)	807 ± 52	11.7 ± 1.6	40.1 ± 5.3	18 ± 4
**PU1** (8.7%TEA)	862 ± 5	13.3 ± 0.1	48.9 ± 0.9	30 ± 2
**PU2** (8.7%TEA)	797 ± 5	12.5 ± 1.5	46.0 ± 5.0	30 ± 3
**PU0** (10%TEA)	829 ± 25	9.6 ± 0.4	36.5 ± 1.4	22 ± 1
**PU1** (10%TEA)	844 ± 34	13.5 ± 0.8	47.7 ± 4.0	28 ± 1
**PU2** (10%TEA)	797 ± 72	12.9 ± 2.6	46.6 ± 8.5	28 ± 2
**PU0** (13%TEA)	614 ± 26	7.1 ± 1.1	19.9 ± 2.4	17 ± 2
**PU1** (13%TEA)	525 ± 34	5.5 ± 0.6	16.7 ± 1.1	15 ± 1
**PU2** (13%TEA)	645 ± 18	8.1 ± 0.4	25.7 ± 1.8	24 ± 3

Average ± standard deviation of 3–4 specimens was shown.

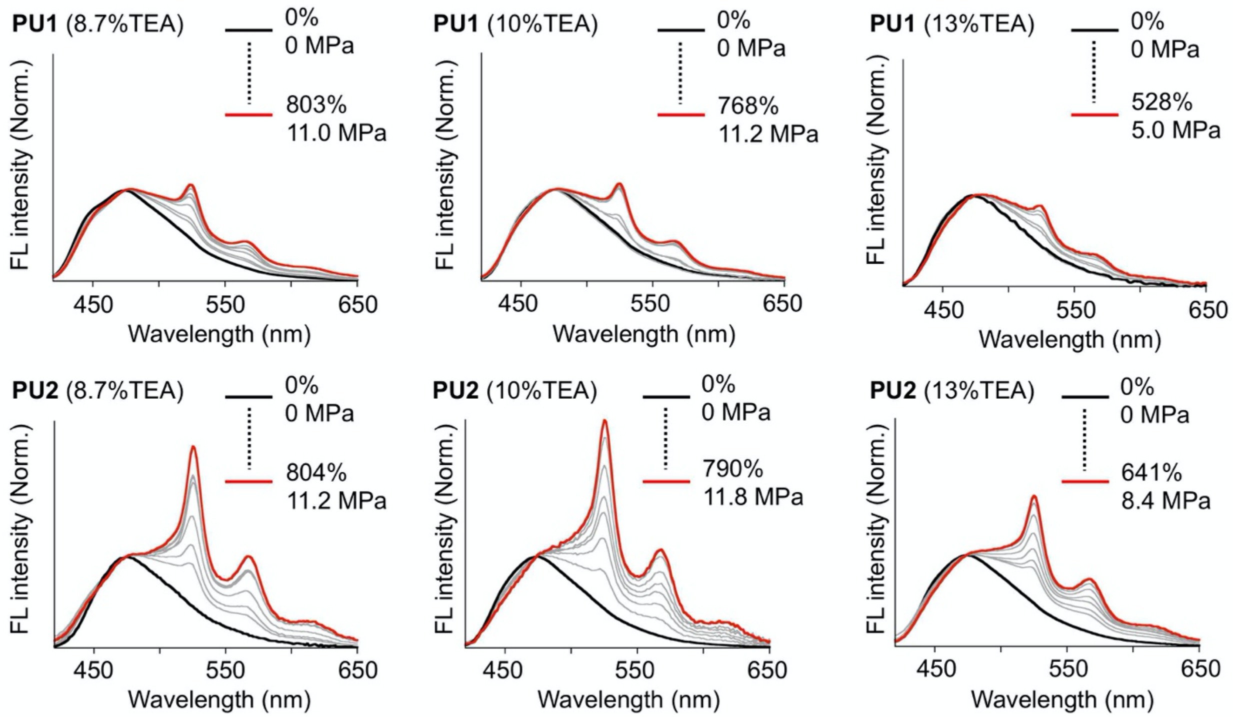

Supplementary Fig. 41. FL spectral changes of the semicrystalline PUs. Normalized at 474 nm. λ_{ex} = 365 nm.

NMR spectra of new compounds

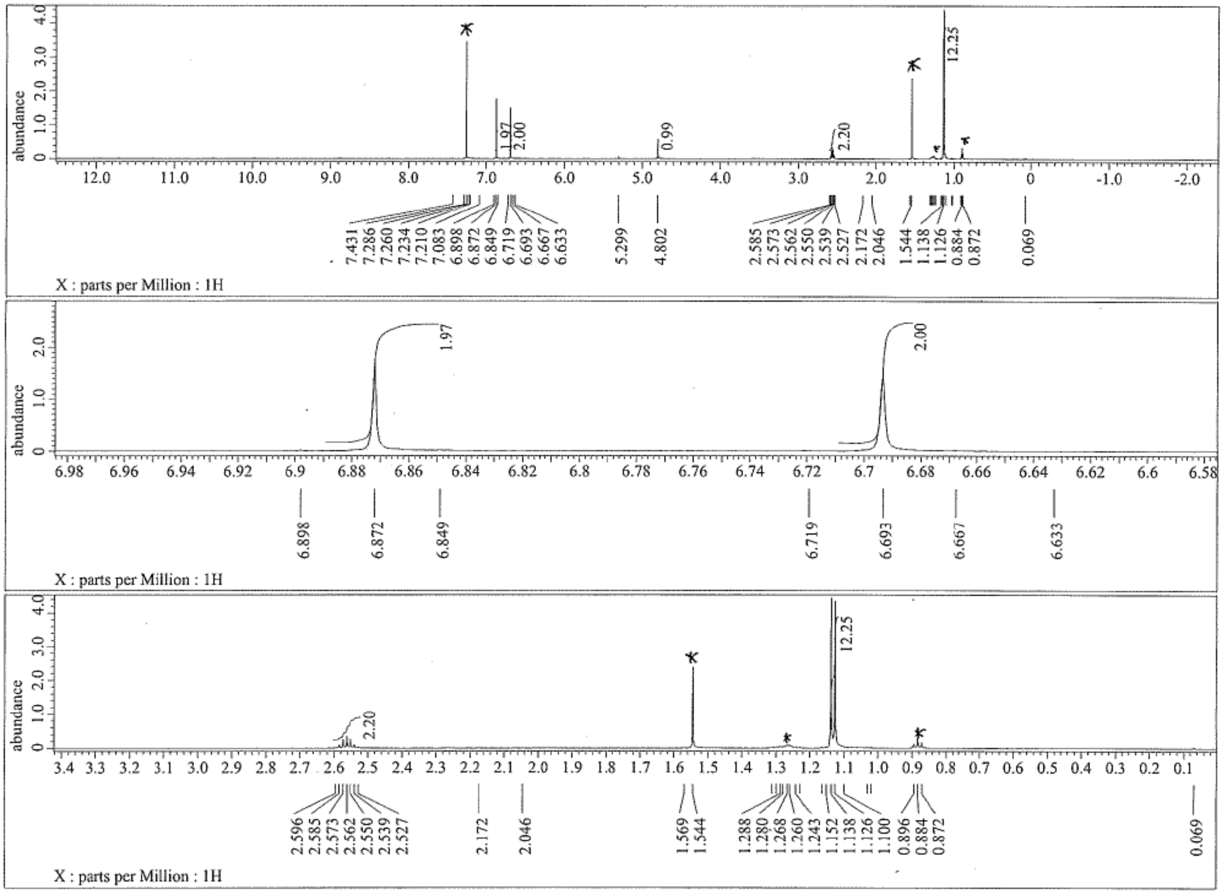

Supplementary Fig. 42. ^{1}H NMR spectra of **S1** in $CDCl_3$ at 25 °C. The peaks marked with * indicate residual solvents.

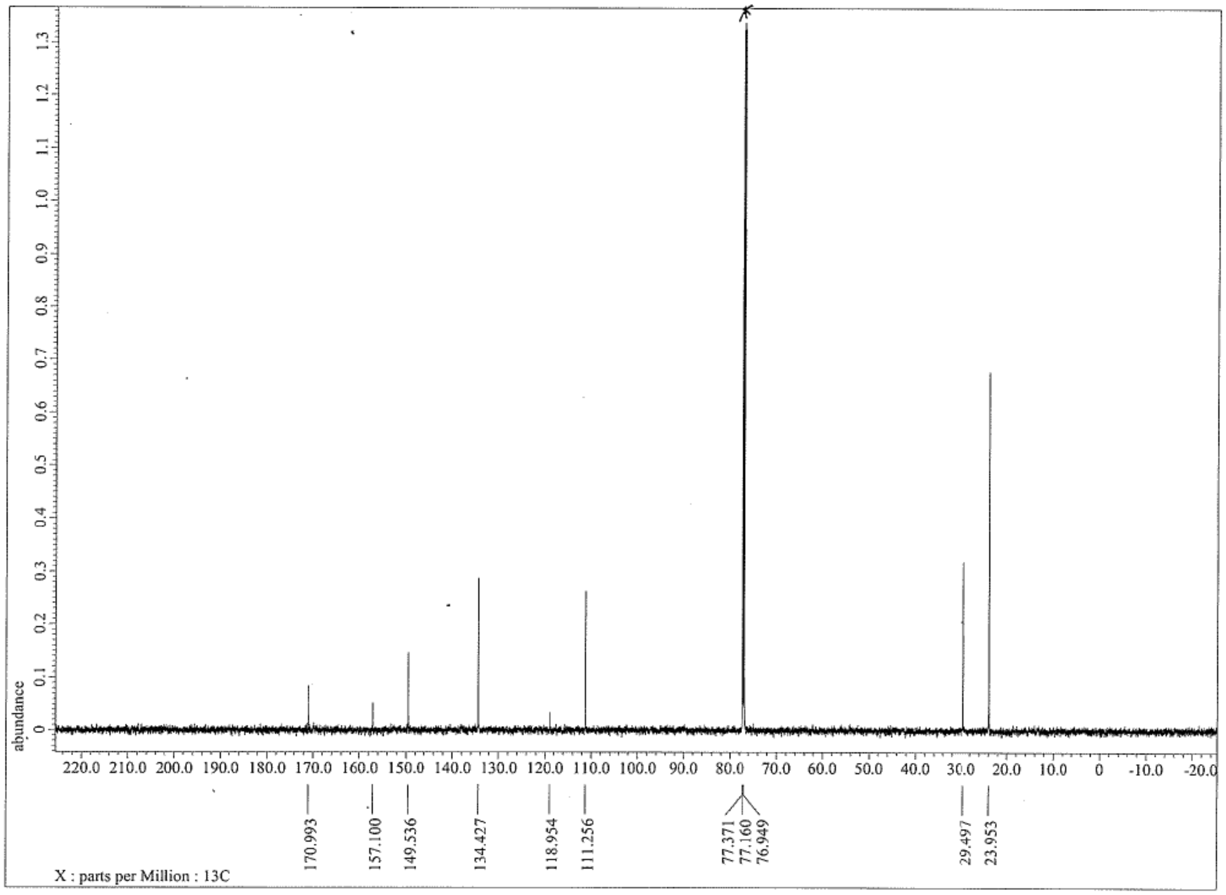

Supplementary Fig. 43. ^{13}C NMR spectrum of **S1** in $CDCl_3$ at 25 °C. The peak marked with * indicates residual solvents.

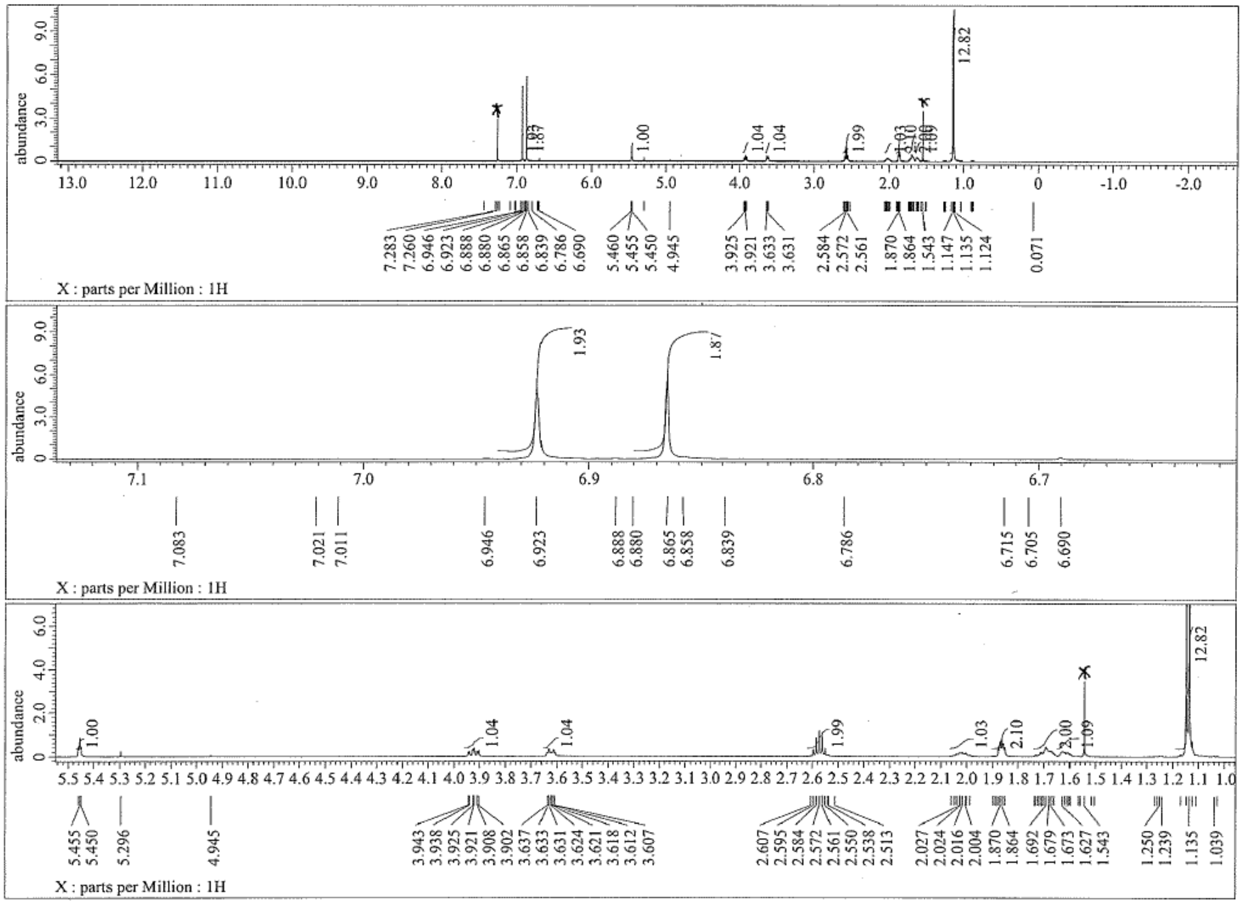

Supplementary Fig. 44. ^1H NMR spectra of **S2** in $CDCl_3$ at 25 °C. The peaks marked with * indicate residual solvents.

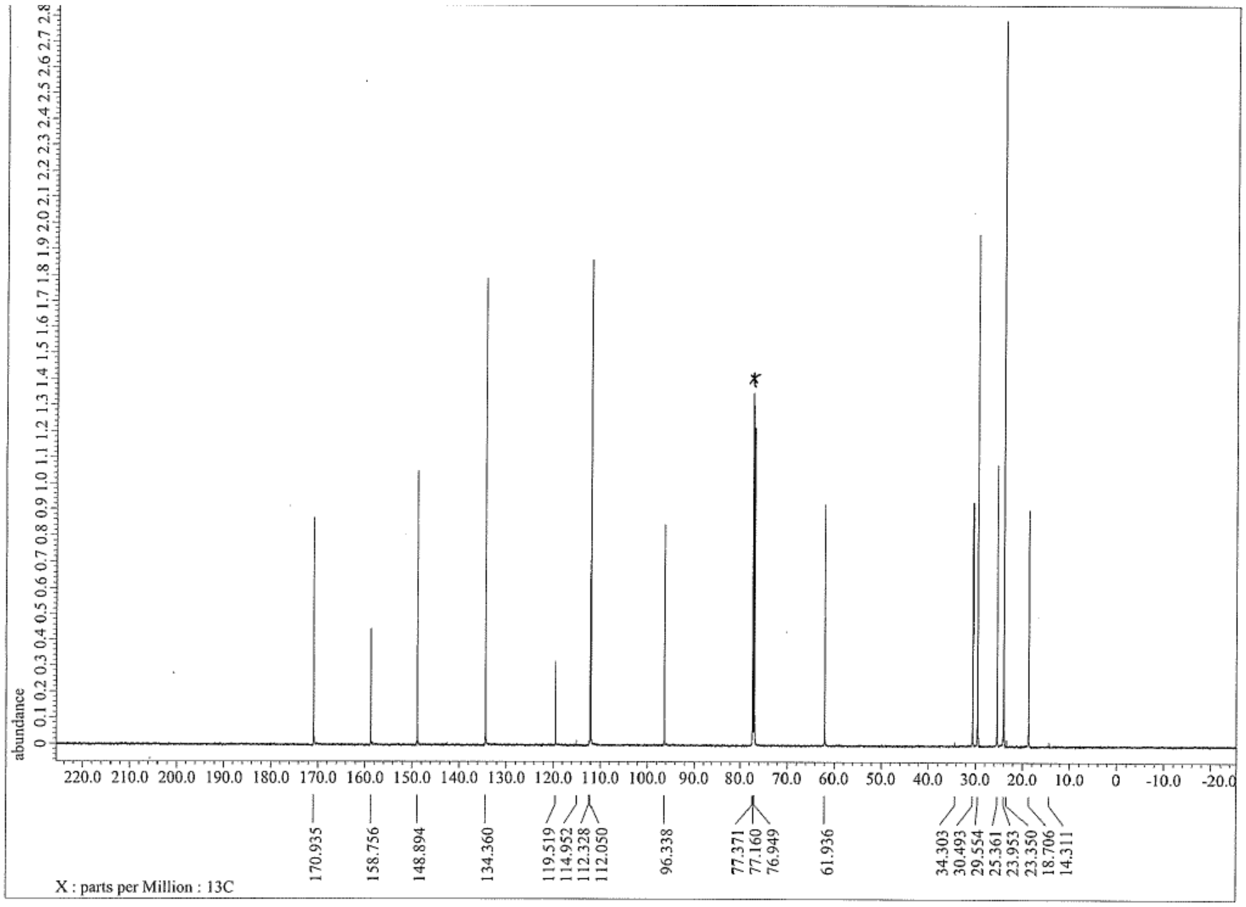

Supplementary Fig. 45. ^{13}C NMR spectrum of **S2** in $CDCl_3$ at 25 °C. The peak marked with * indicates residual solvents.

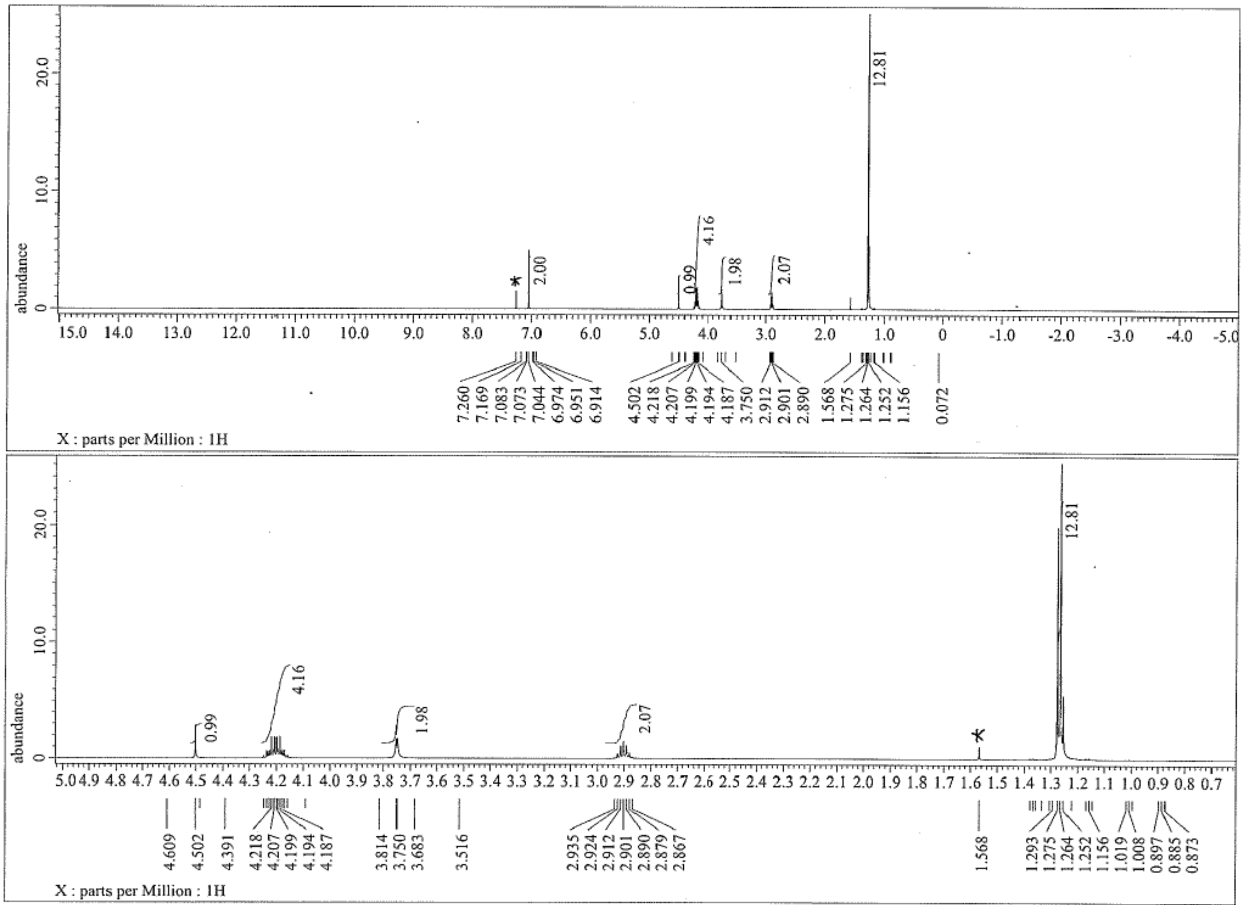

Supplementary Fig. 46. ^{1}H NMR spectra of **S3** in $CDCl_3$ at 25 °C. The peaks marked with * indicate residual solvents.

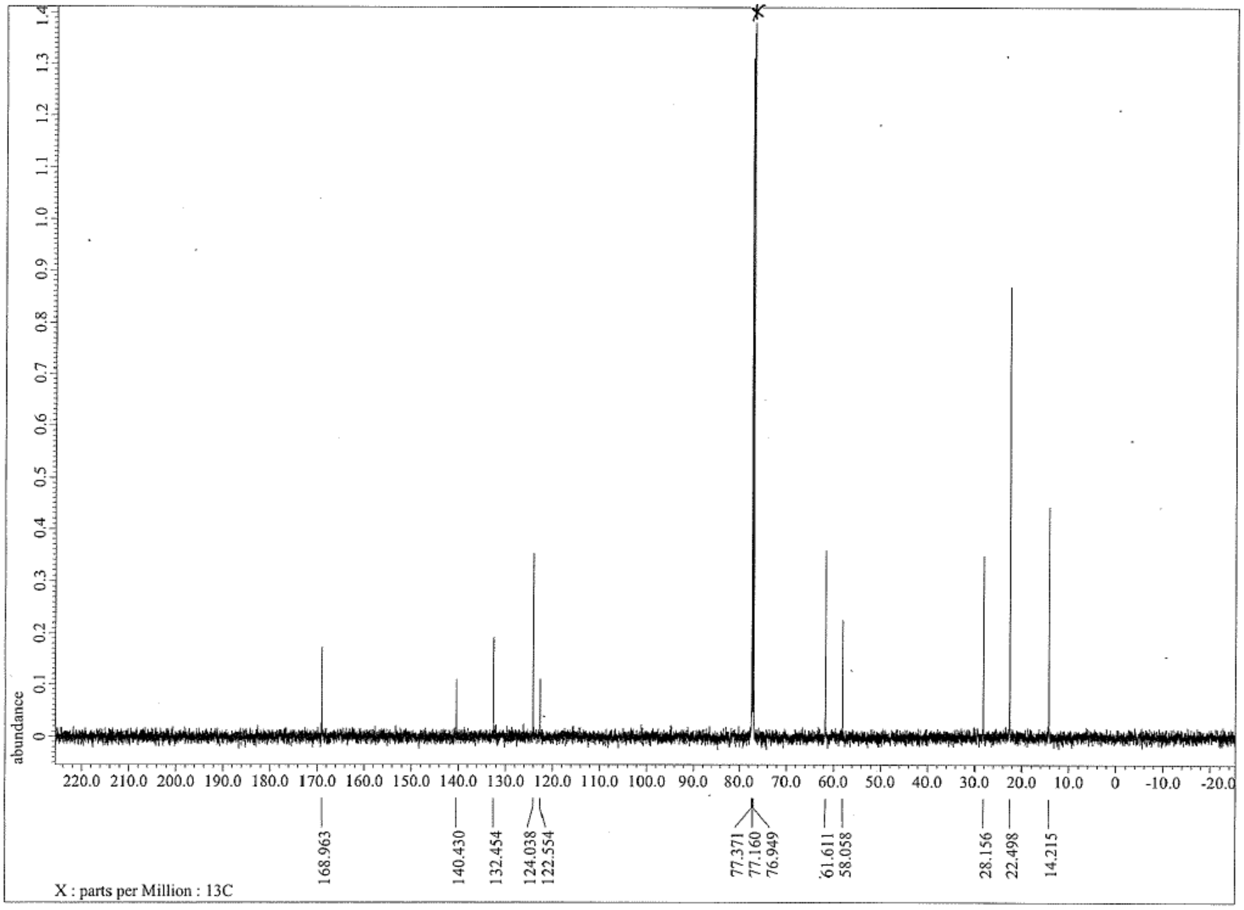

Supplementary Fig. 47. ^{13}C NMR spectrum of **S3** in $CDCl_3$ at 25 °C. The peak marked with * indicates residual solvents.

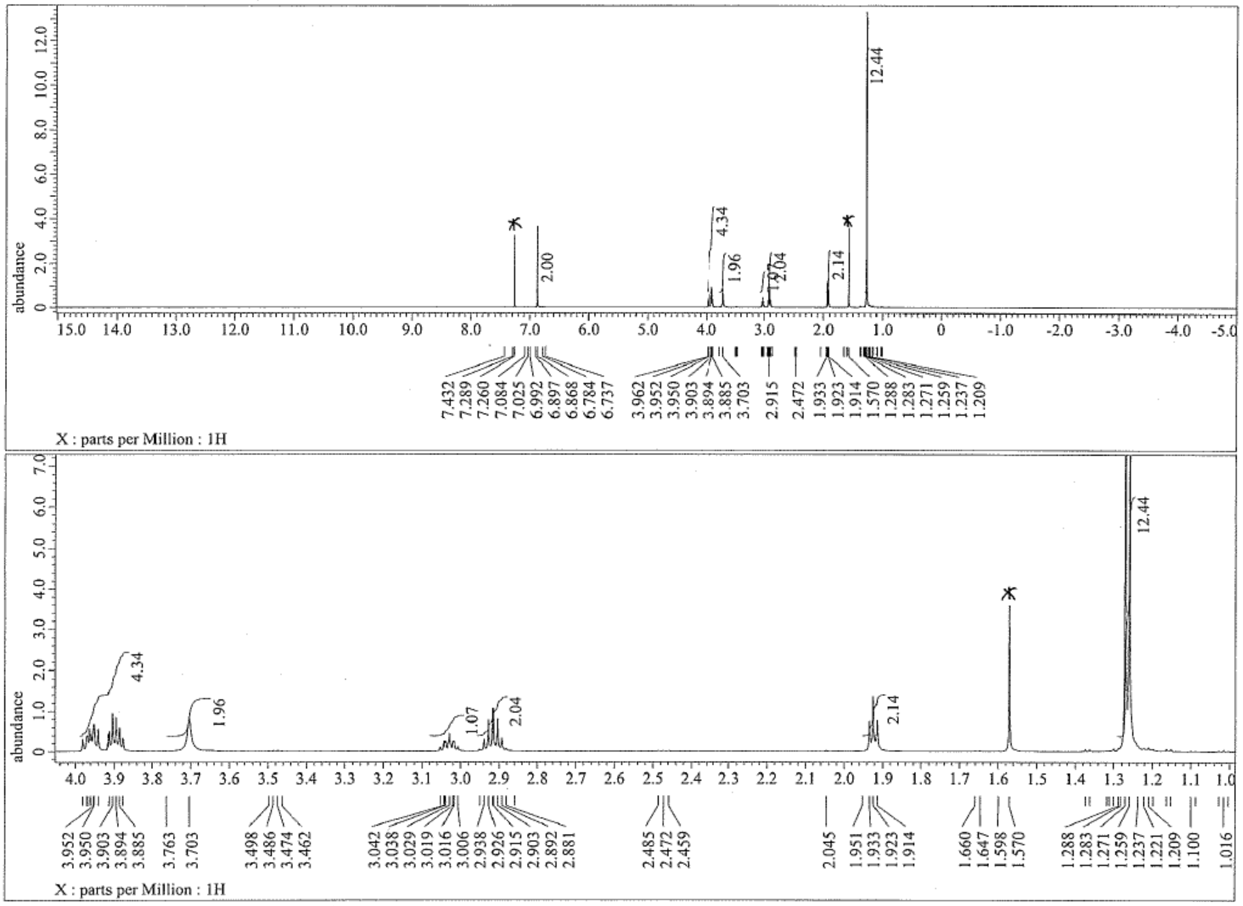

Supplementary Fig. 48. ^{1}H NMR spectra of **S4** in $CDCl_3$ at 25 °C. The peaks marked with * indicate residual solvents.

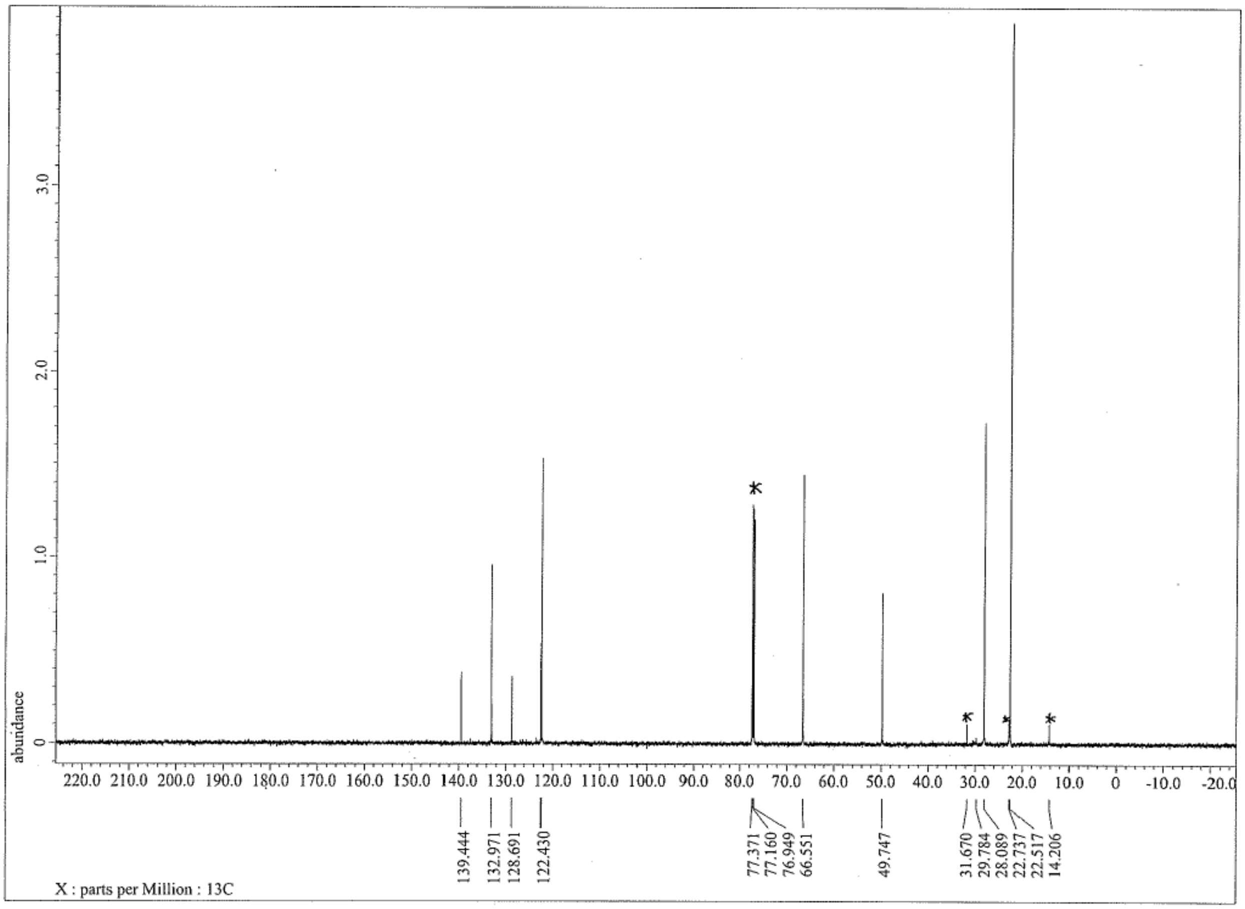

Supplementary Fig. 49. ^{13}C NMR spectrum of **S4** in $CDCl_3$ at 25 °C. The peaks marked with * indicate residual solvents.

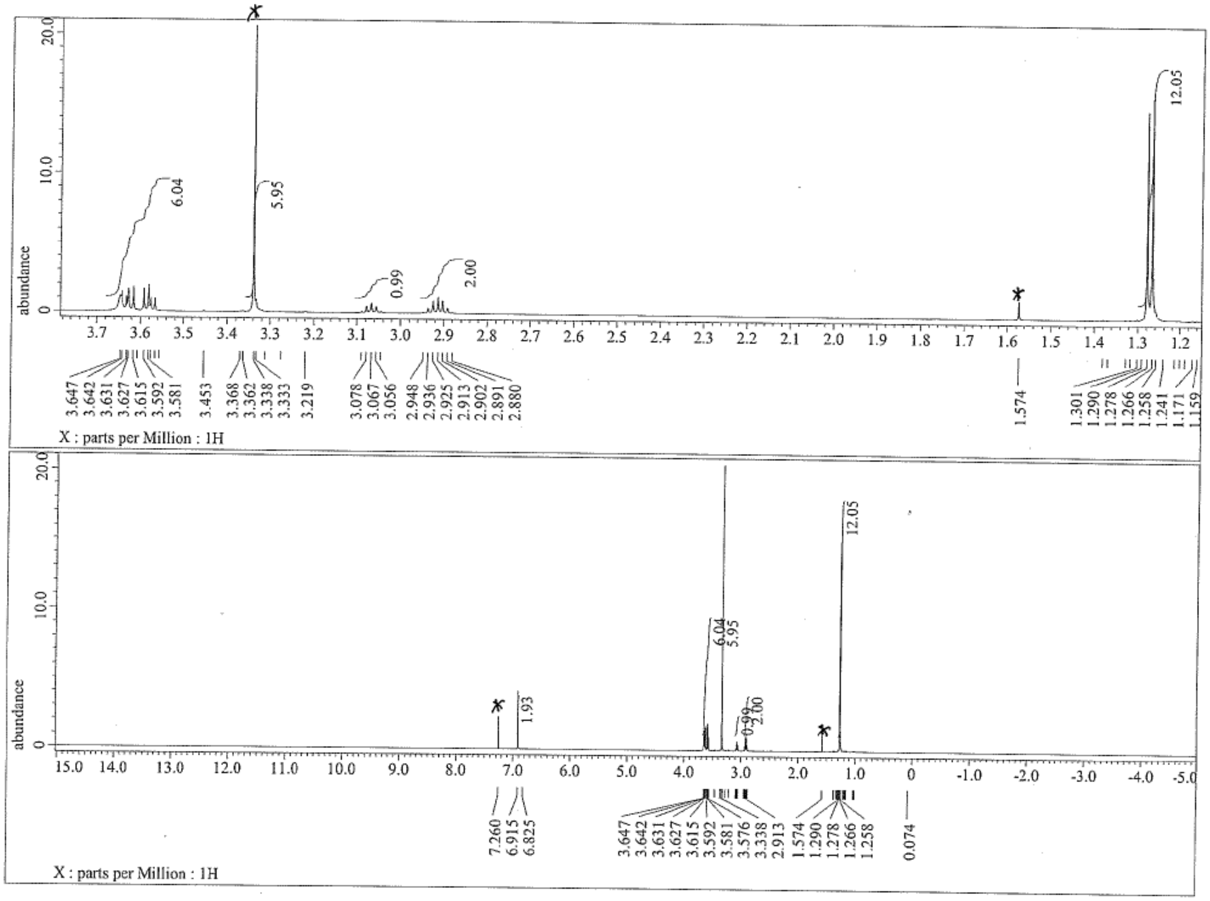

Supplementary Fig. 50. ^{1}H NMR spectra of **S5** in $CDCl_3$ at 25 °C. The peaks marked with * indicate residual solvents.

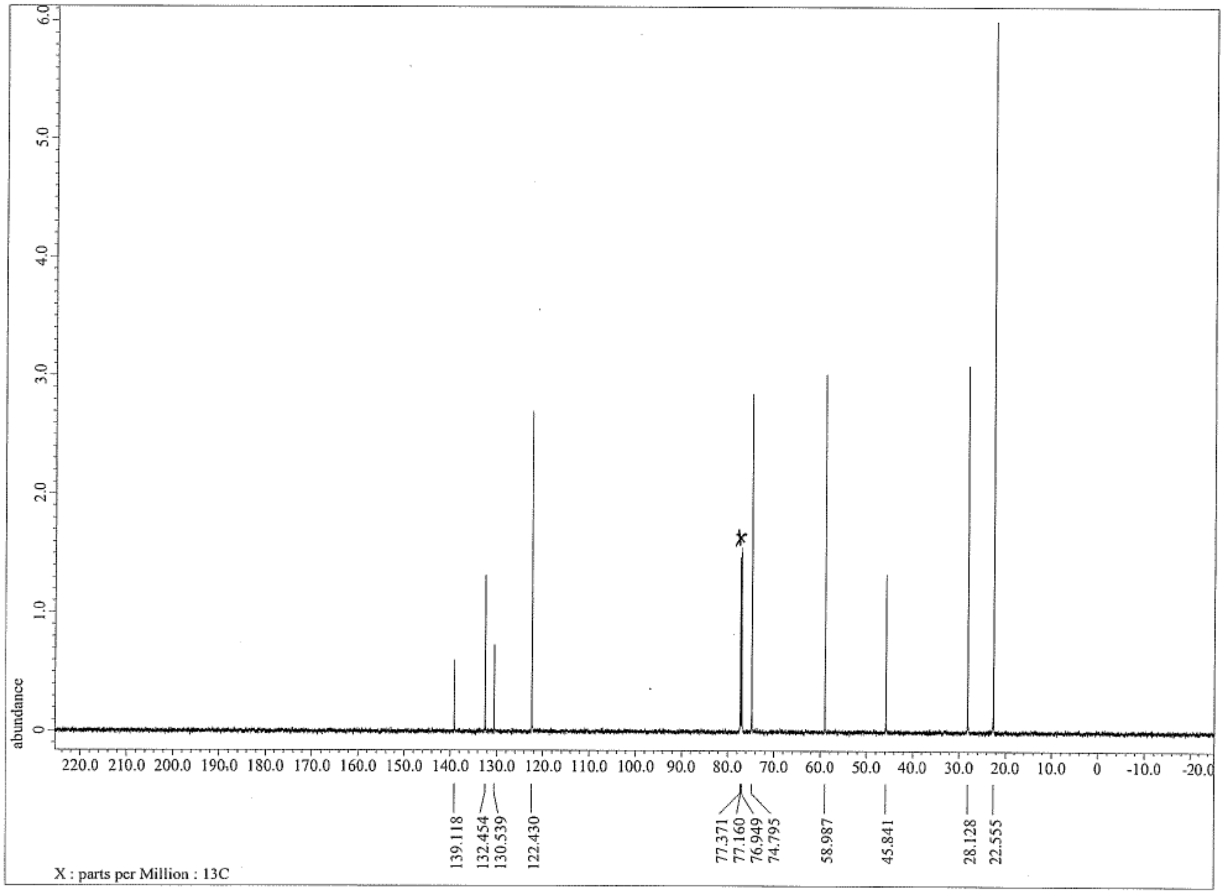

Supplementary Fig. 51. ^{13}C NMR spectrum of **S5** in $CDCl_3$ at 25 °C. The peak marked with * indicates residual solvents.

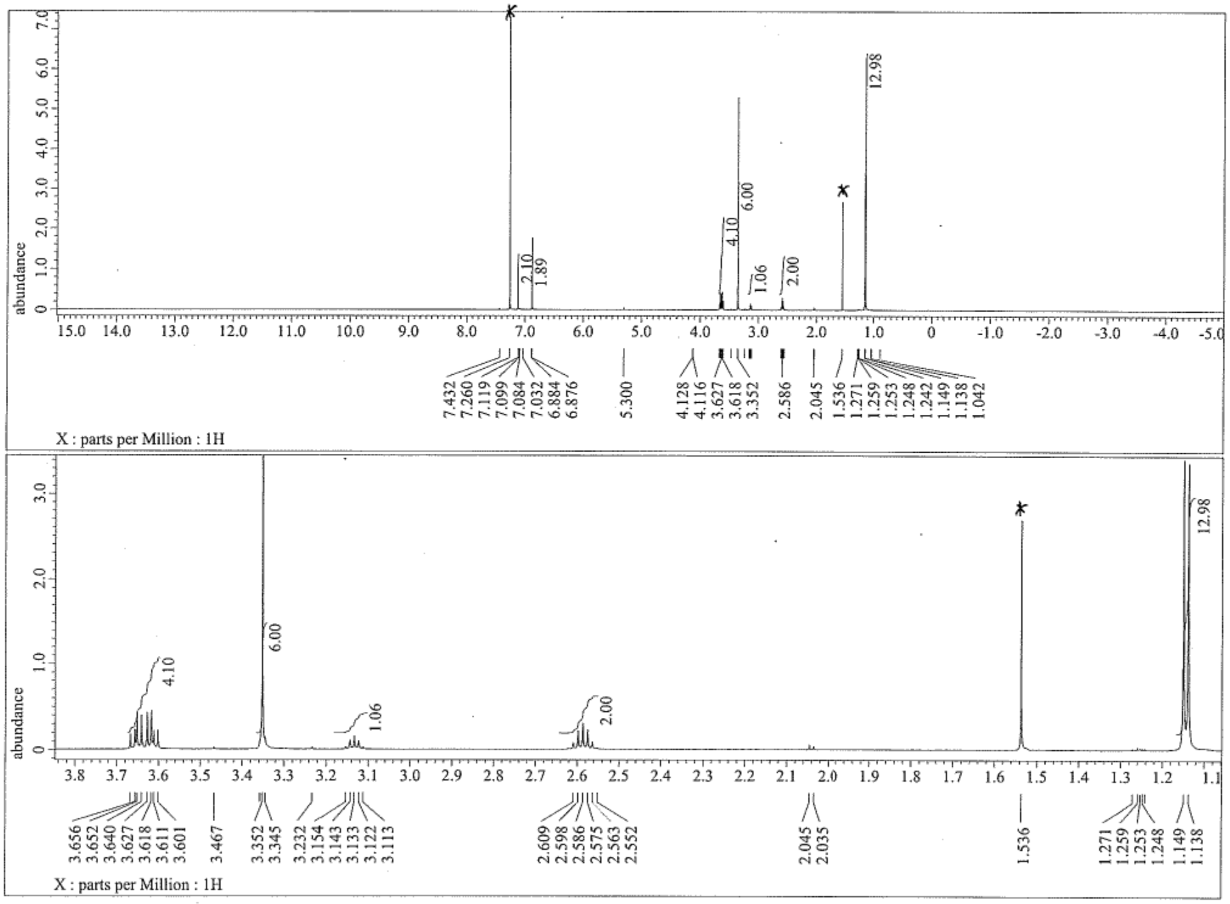

Supplementary Fig. 52. ^{1}H NMR spectra of **S6** in $CDCl_3$ at 25 °C. The peaks marked with * indicate residual solvents.

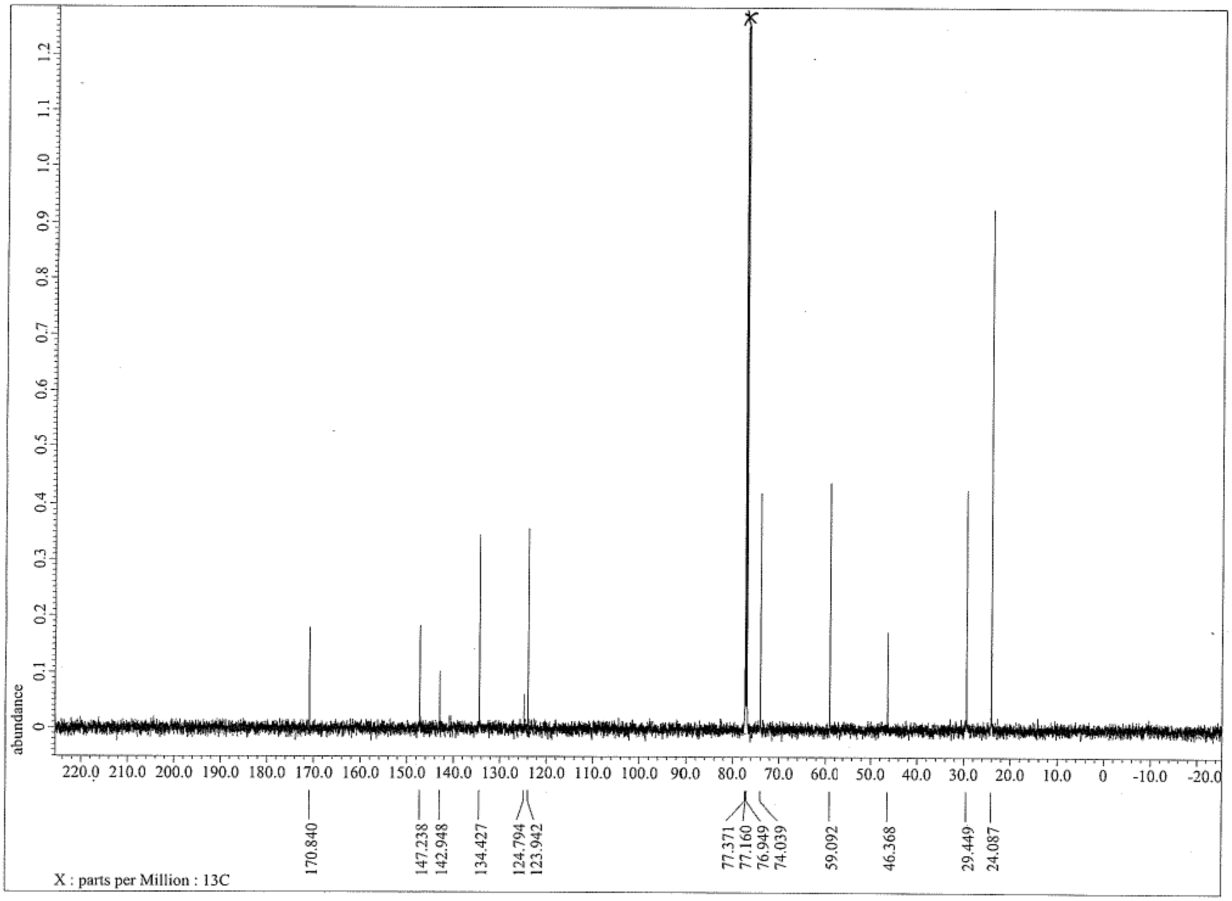

Supplementary Fig. 53. ^{13}C NMR spectrum of **S6** in $CDCl_3$ at 25 °C. The peak marked with * indicates residual solvents.

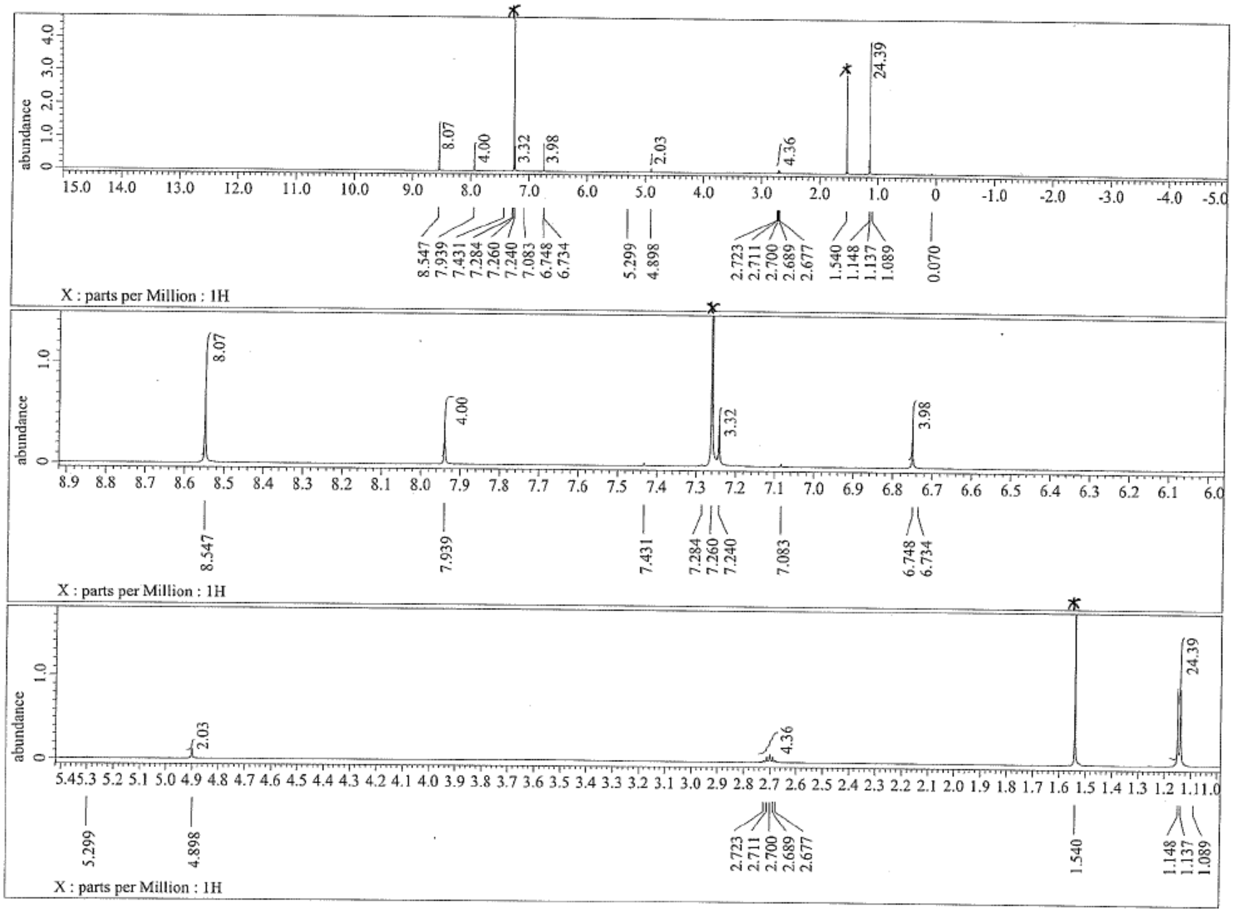

Supplementary Fig. 54. ^{1}H NMR spectra of **FLAP1** in $CDCl_3$ at 25 °C. The peaks marked with * indicate residual solvents.

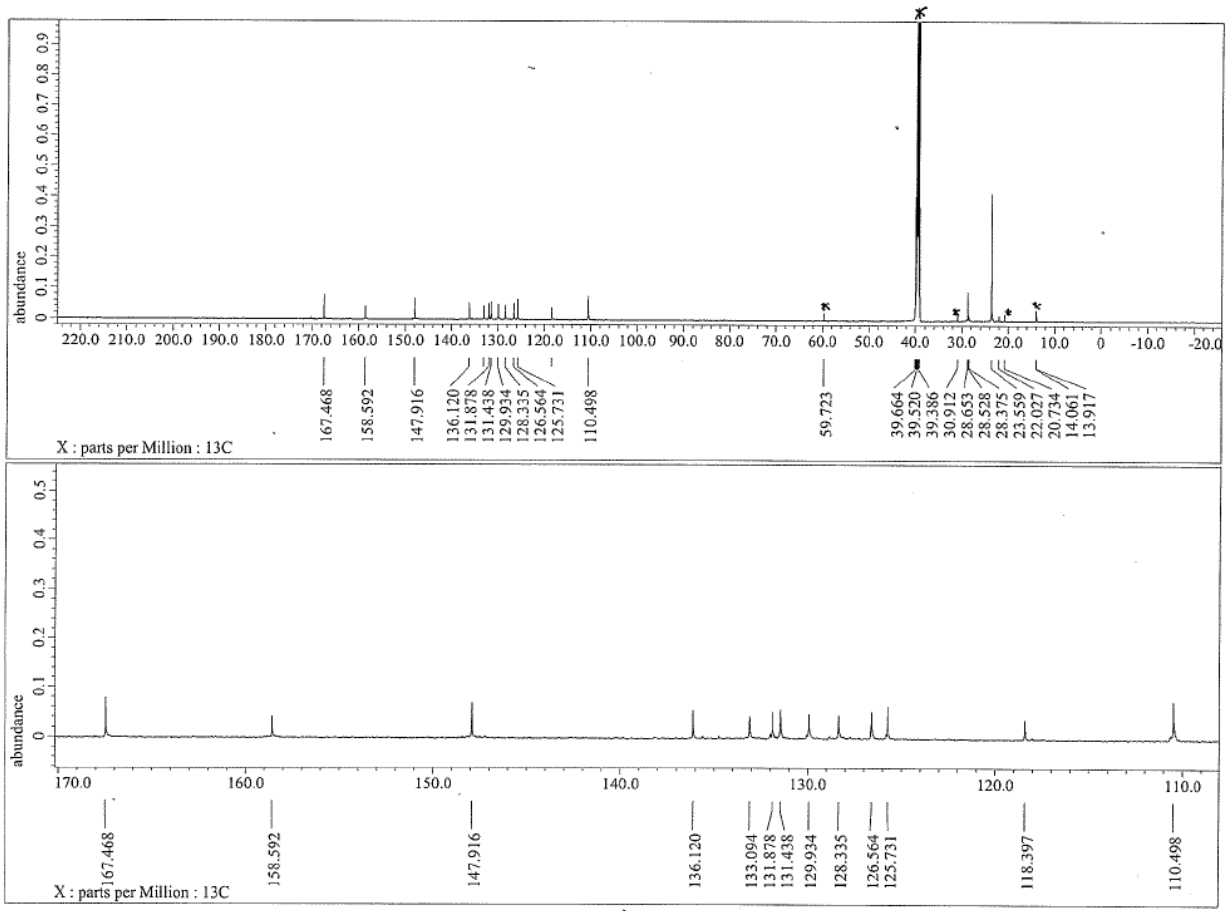

Supplementary Fig. 55. ^{13}C NMR spectra of **FLAP1** in DMSO-d_6 at 25 °C. The peaks marked with * indicate residual solvents.

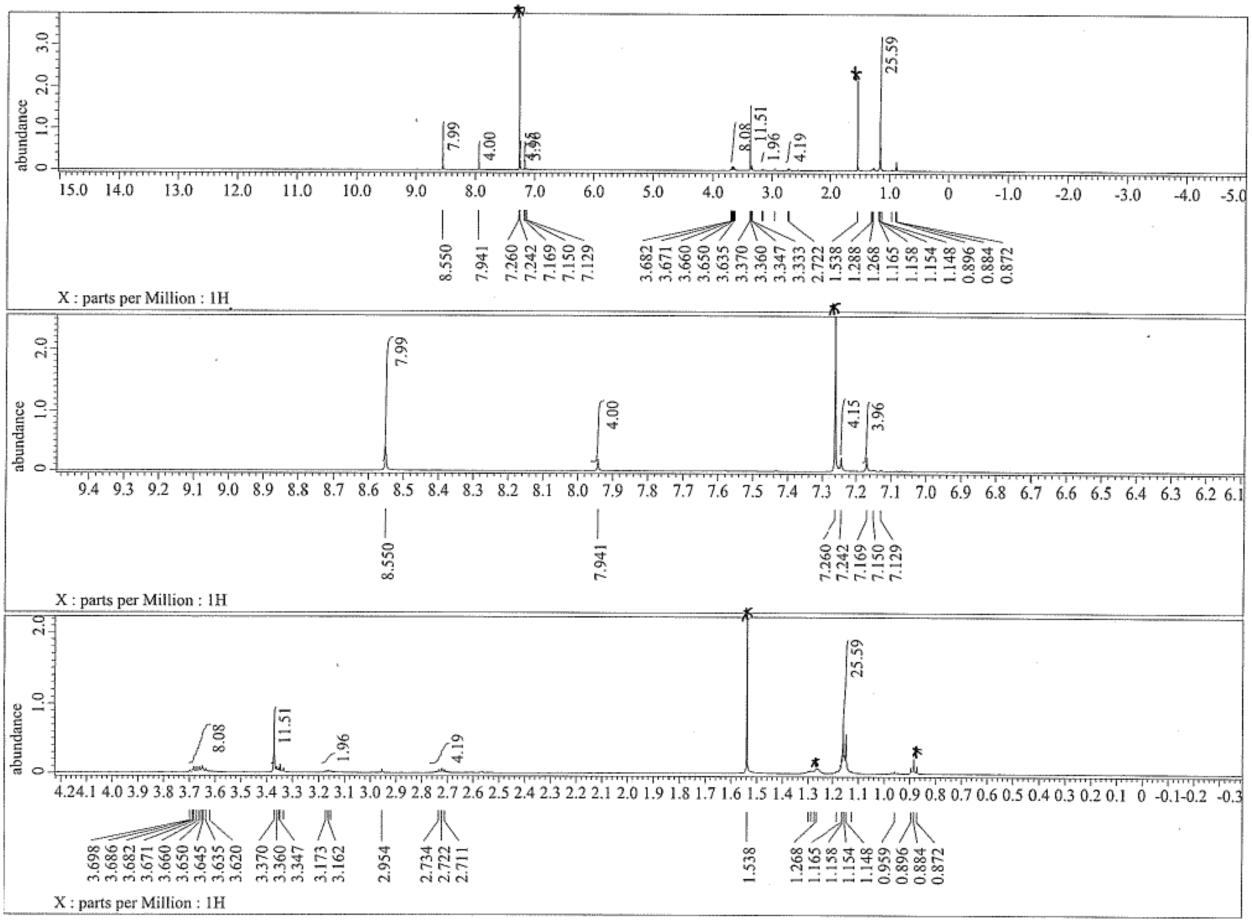

Supplementary Fig. 56. ^{1}H NMR spectra of **S8** in $CDCl_3$ at 25 °C. The peaks marked with * indicate residual solvents.

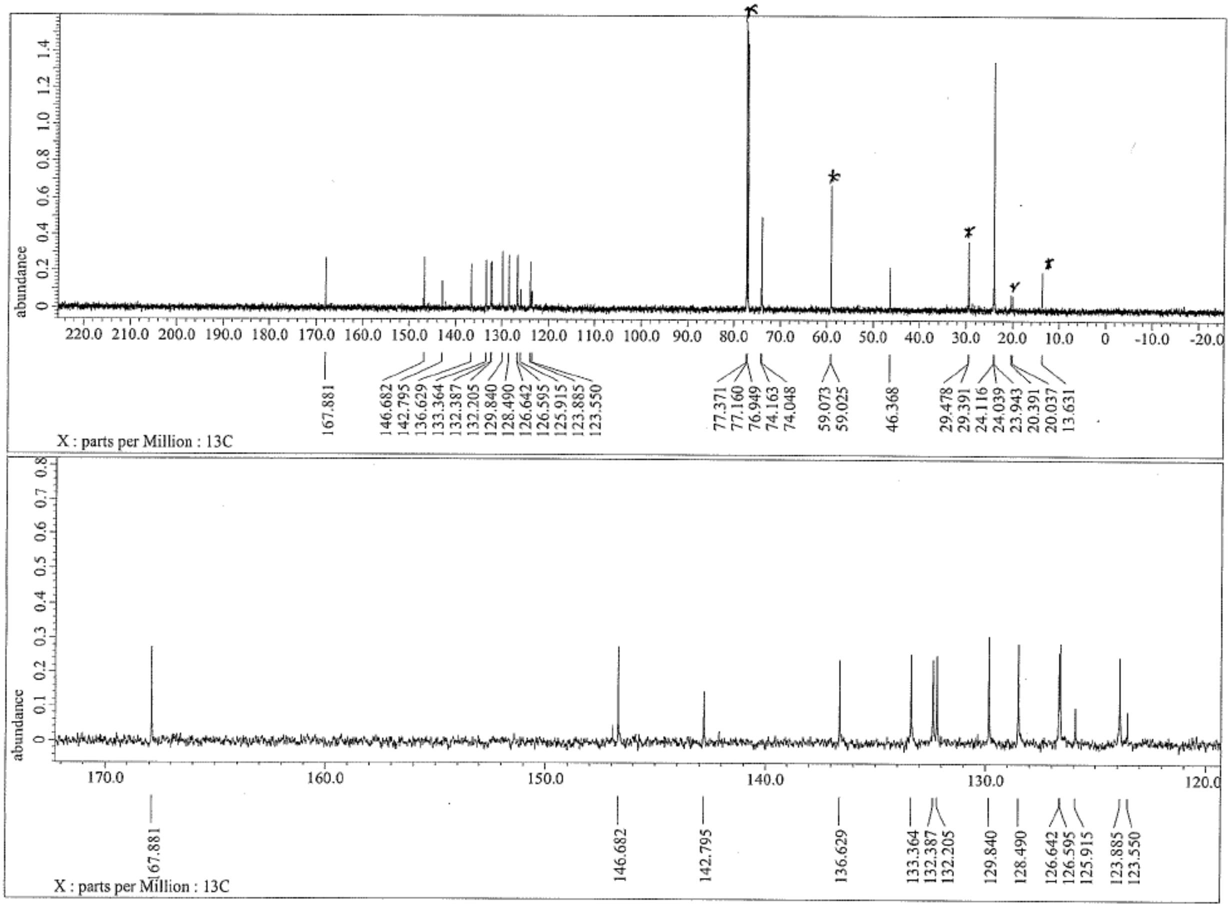

Supplementary Fig. 57. ^{13}C NMR spectra of **S8** in $CDCl_3$ at 25 °C. The peaks marked with * indicate residual solvents.

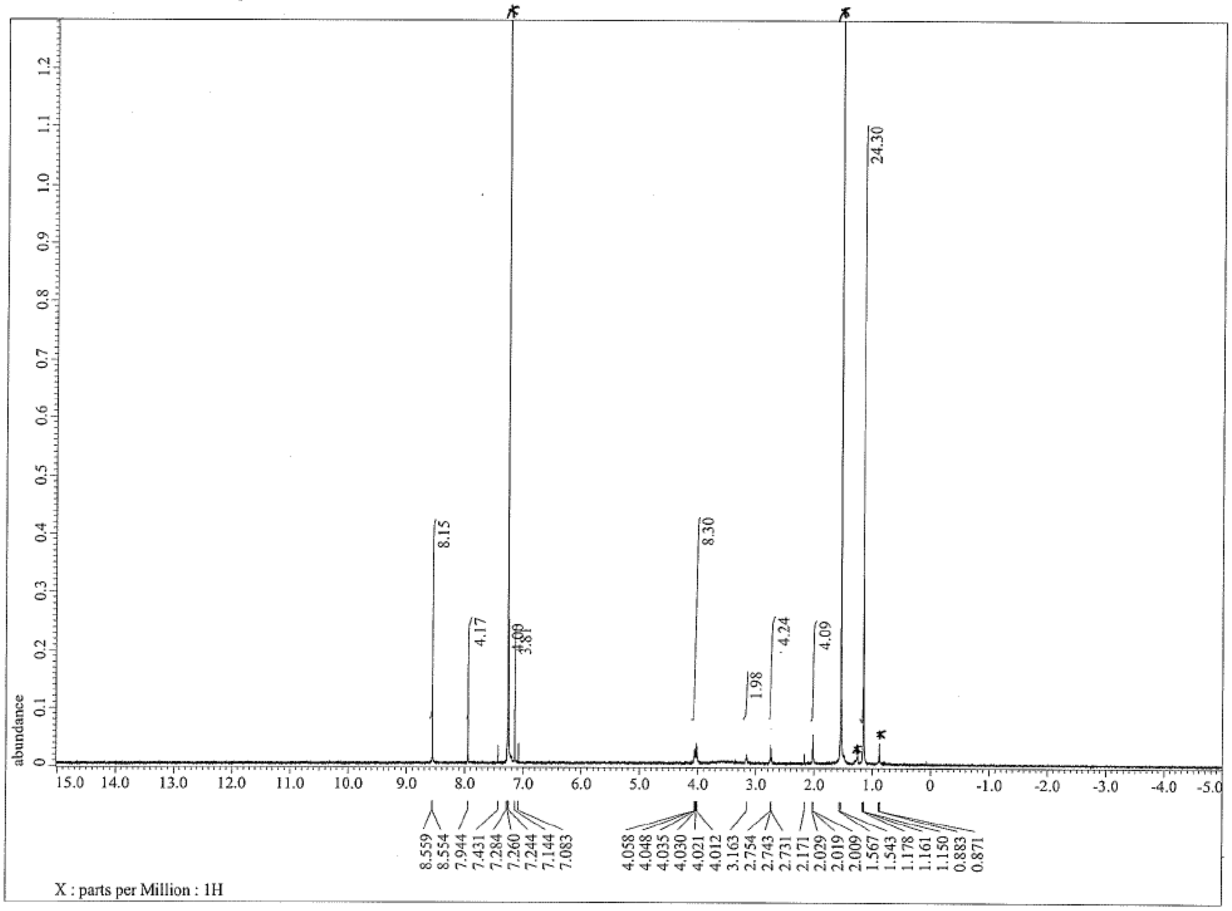

Supplementary Fig. 58. ^{1}H NMR spectrum of **FLAP2** in $CDCl_3$ at 25 °C. The peaks marked with * indicate residual solvents.

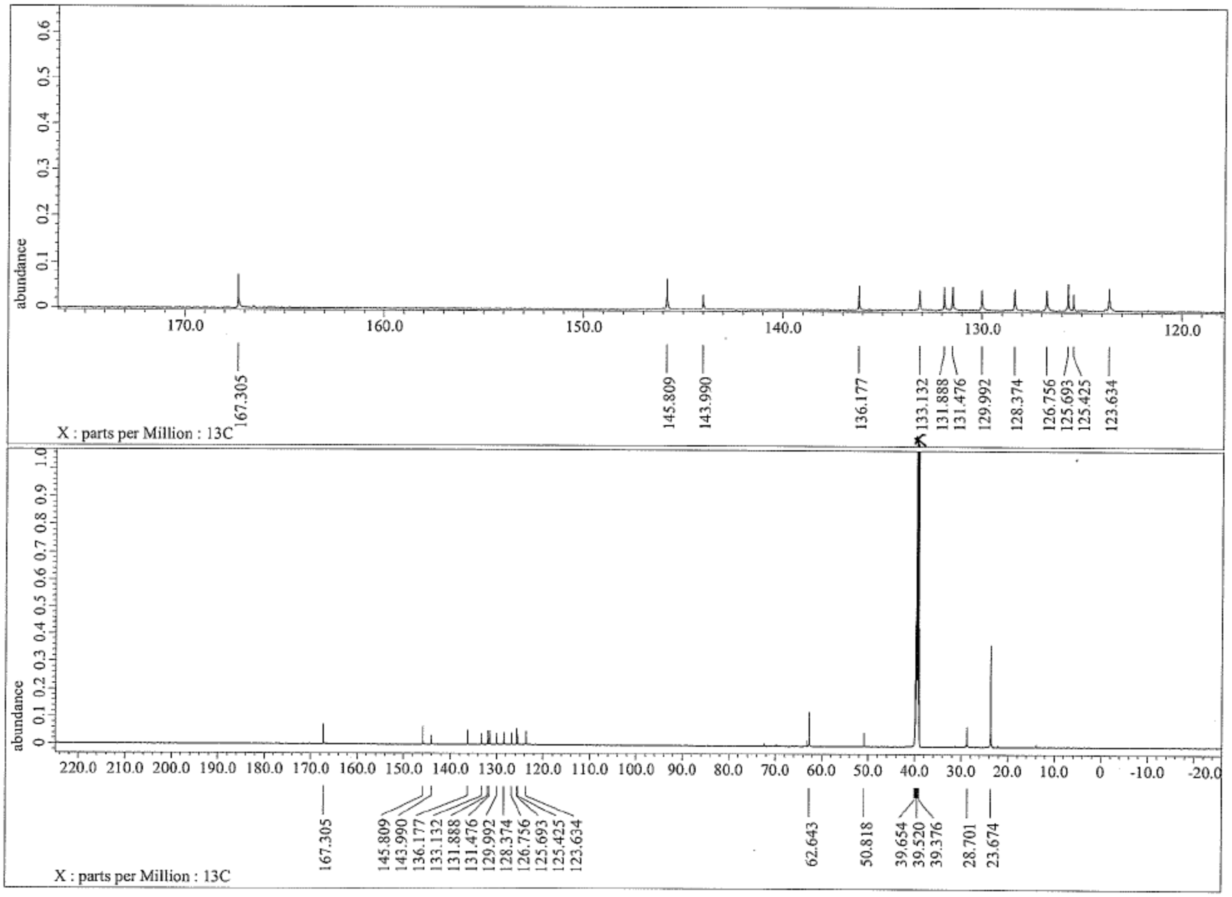

Supplementary Fig. 59. ^{13}C NMR spectra of **FLAP2** in DMSO-d_6 at 25 °C. The peak marked with * indicates residual solvents.

Mass spectra of new compounds

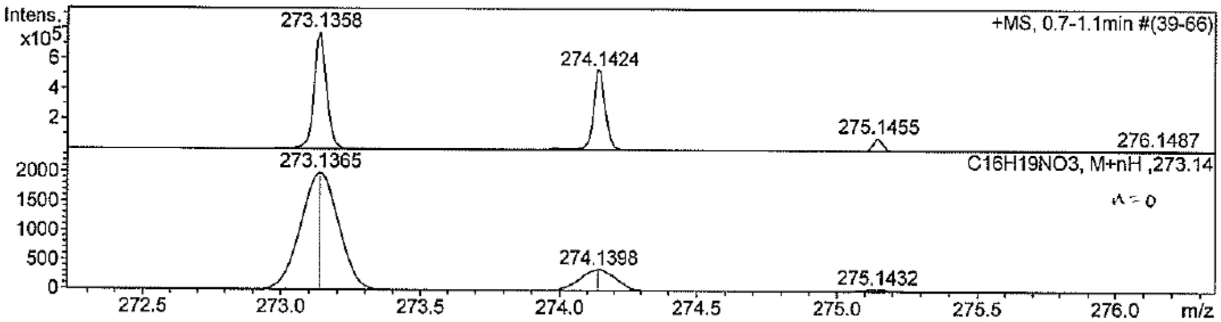

Supplementary Fig. 60. HR-APCI-TOF mass spectra of **S1**. Top: observed, bottom: simulated.

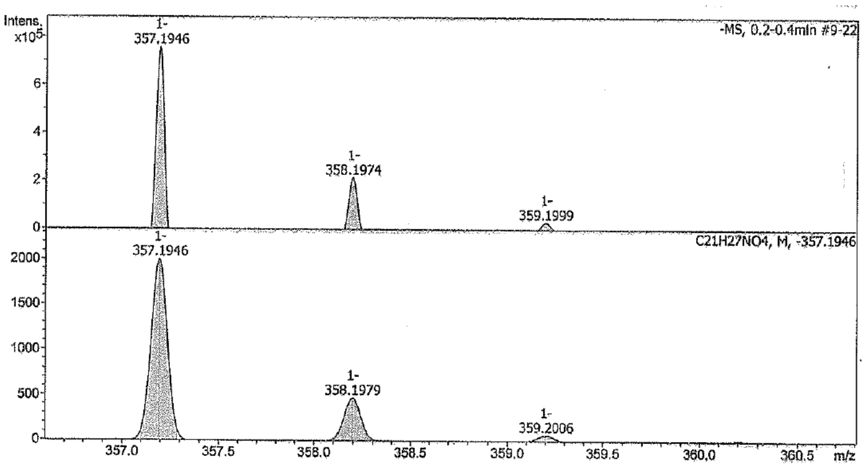

Supplementary Fig. 61. HR-APCI-TOF mass spectra of **S2**. Top: observed, bottom: simulated.

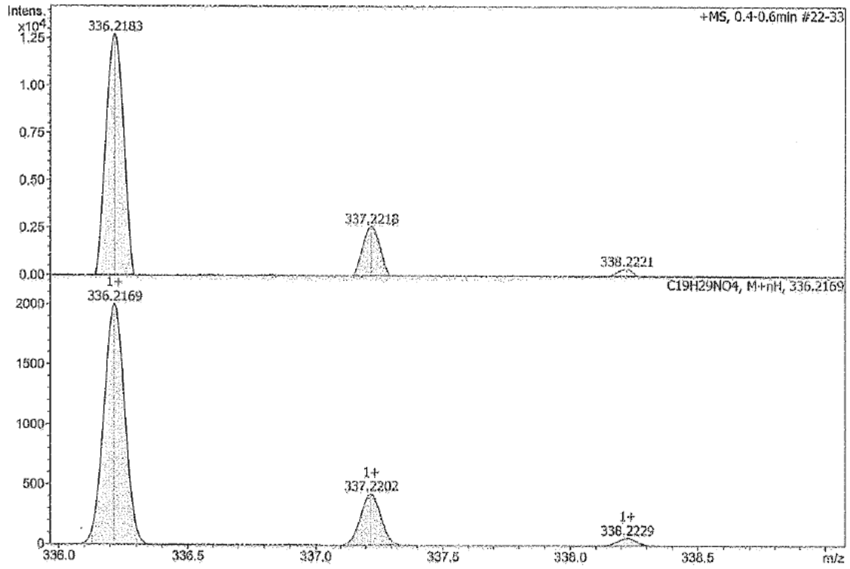

Supplementary Fig. 62. HR-APCI-TOF mass spectra of **S3**. Top: observed, bottom: simulated.

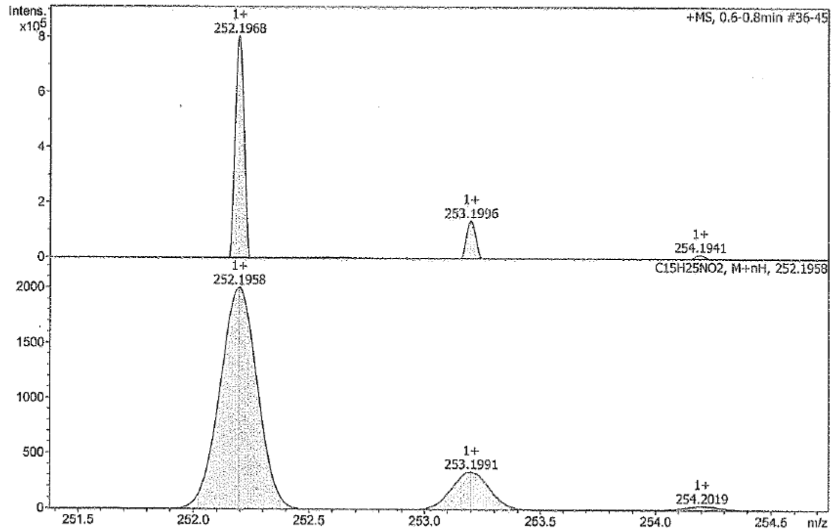

Supplementary Fig. 63. HR-APCI-TOF mass spectra of **S4**. Top: observed, bottom: simulated.

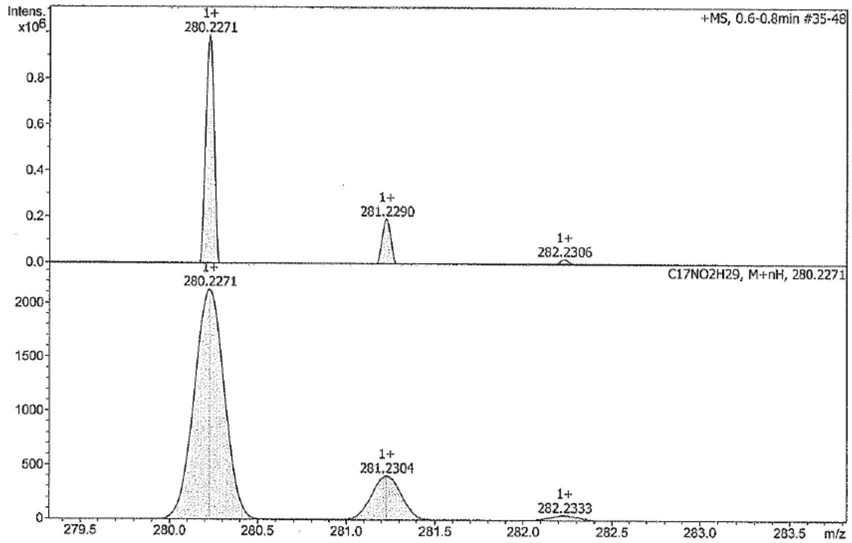

Supplementary Fig. 64. HR-APCI-TOF mass spectra of **S5**. Top: observed, bottom: simulated.

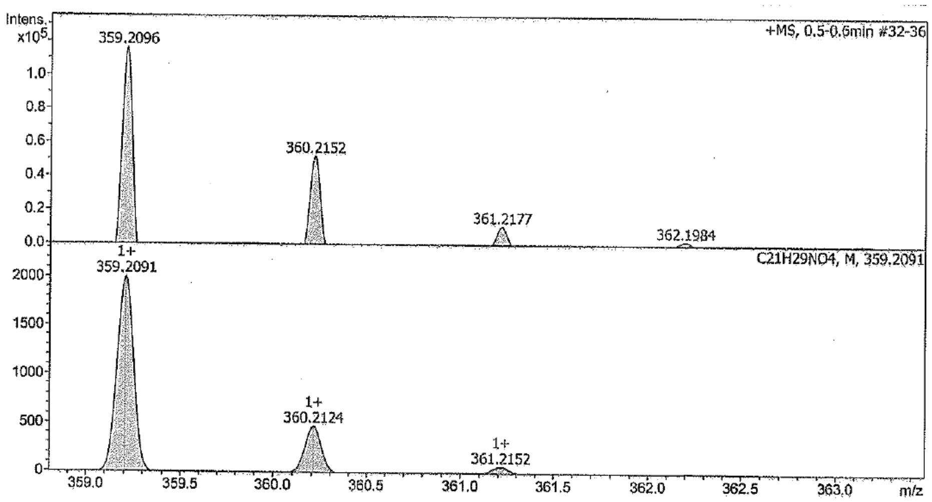

Supplementary Fig. 65. HR-TOF mass spectra of **S6**. Top: observed, bottom: simulated.

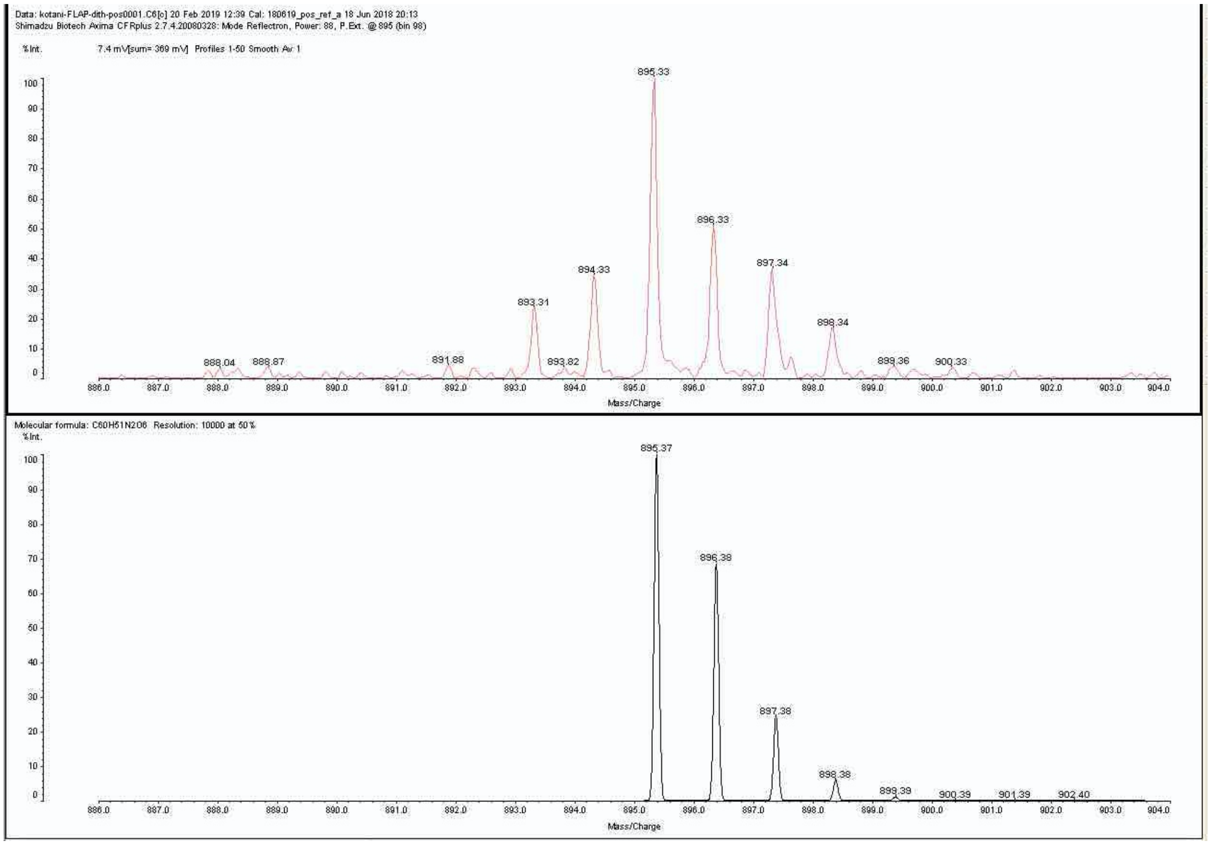

Supplementary Fig. 66. HR-MALDI-TOF mass spectra of **FLAP1**. Top: observed, bottom: simulated.

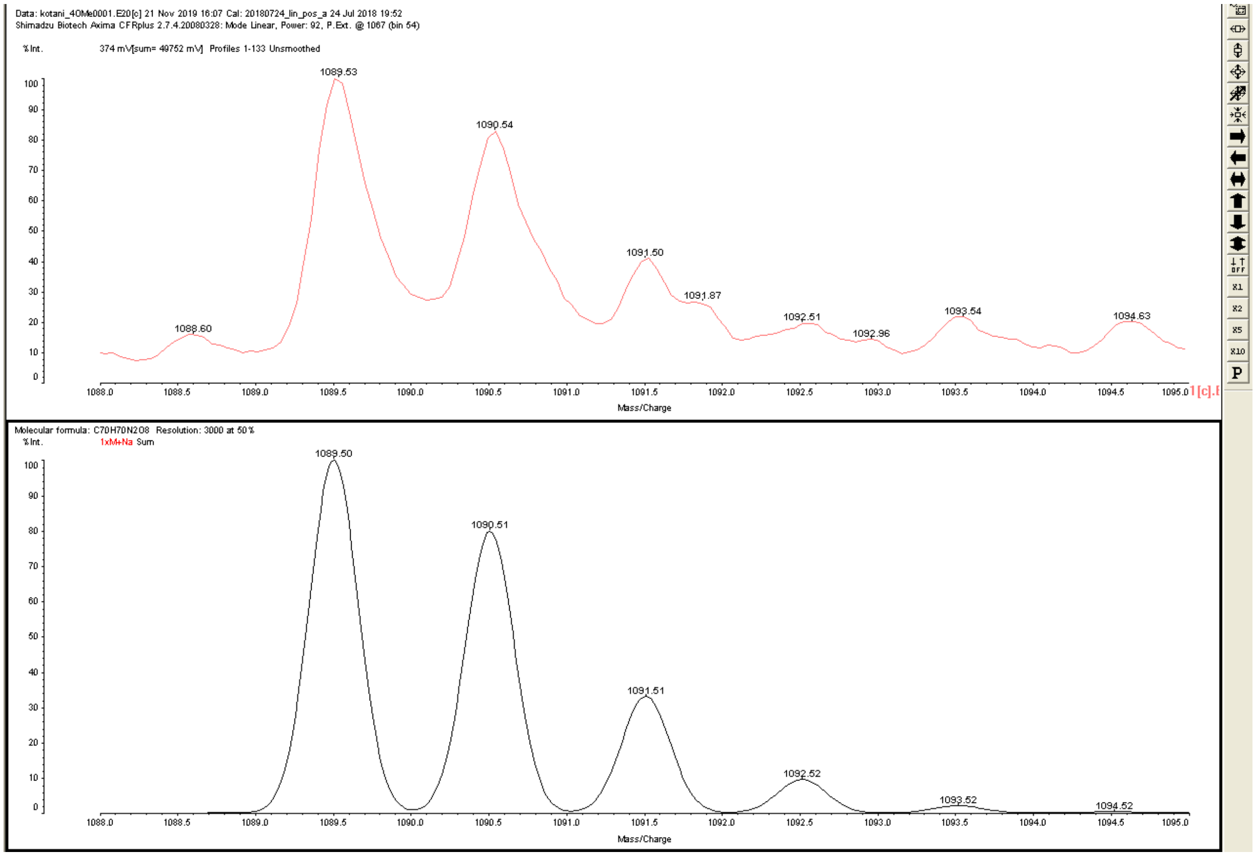

Supplementary Fig. 67. HR-MALDI-TOF mass spectra of **S8**. Top: observed, bottom: simulated.

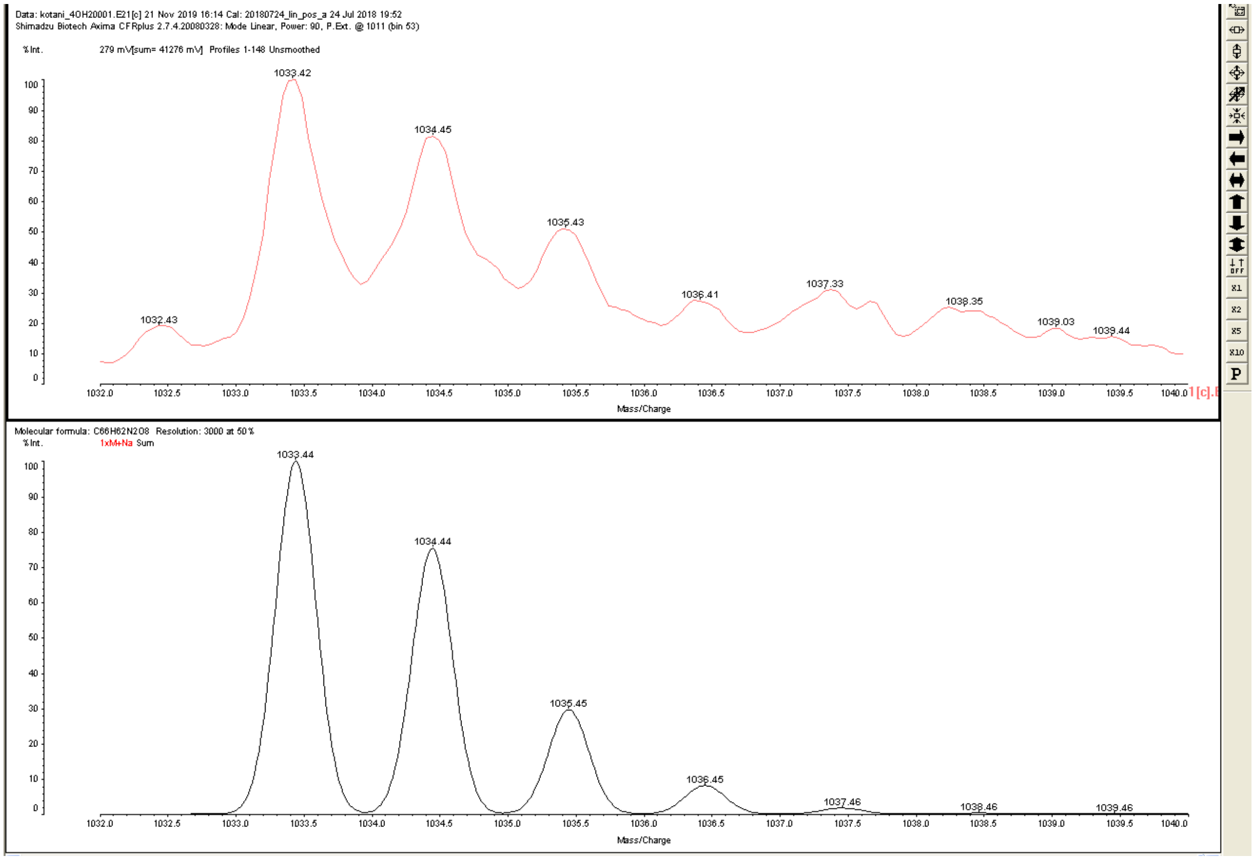

Supplementary Fig. 68. HR-MALDI-TOF mass spectra of **FLAP2**. Top: observed, bottom: simulated.

Supplementary References

1. Krinsky, J. L., Martínez, A., Godard, C., Castillón, S. & Claver, C. Modular Synthesis of Functionalisable Alkoxy-Tethered N-Heterocyclic Carbene Ligands and an Active Catalyst for Buchwald–Hartwig Aminations. *Adv. Syn. Cat.* **356**, 460–474 (2014).
2. Zhang, L., Ren, X., Zhang, Y. & Zhang, K. Step-Growth Polymerization Method for Ultrahigh Molecular Weight Polymers. *ACS Macro Lett.* **8**, 948–954 (2019).
3. Yuan, C., Saito, S., Camacho, C., Irle, S., Hisaki, I. & Yamaguchi, S. A π-conjugated System with Flexibility and Rigidity That Shows Environment-Dependent RGB Luminescence. *J. Am. Chem. Soc.* **135**, 8842–8845 (2013).
4. Kotani, R., Sotome, H., Okajima, H., Yokoyama, S., Nakaike, Y., Kashiwagi, A., Mori, C., Nakada, Y., Yamaguchi, S., Osuka, A., Sakamoto, A., Miyasaka, H. & Saito, S. Flapping viscosity probe that shows polarity-independent ratiometric fluorescence. *J. Mater. Chem. C.* **5**, 5248–5256 (2017).
5. Gaussian 16, Revision A.03, Frisch, M. J., Trucks, G. W., Schlegel, H. B., Scuseria, G. E., Robb, M. A., Cheeseman, J. R., Scalmani, G., Barone, V., Petersson, G. A., Nakatsuji, H., Li, X., Caricato, M., Marenich, A. V., Bloino, J., Janesko, B. G., Gomperts, R., Mennucci, B., Hratchian, H. P., Ortiz, J. V., Izmaylov, A. F., Sonnenberg, J. L., Williams-Young, D., Ding, F., Lipparini, F., Egidi, F., Goings, J., Peng, B., Petrone, A., Henderson, T., Ranasinghe, D., Zakrzewski, V. G., Gao, J., Rega, N., Zheng, G., Liang, W., Hada, M., Ehara, M., Toyota, K., Fukuda, R., Hasegawa, J., Ishida, M., Nakajima, T., Honda, Y., Kitao, O., Nakai, H., Vreven, T., Throssell, K., Montgomery, J. A., Jr., Peralta, J. E., Ogliaro, F., Bearpark, M. J., Heyd, J. J., Brothers, E. N., Kudin, K. N., Staroverov, V. N., Keith, T. A., Kobayashi, R., Normand, J., Raghavachari, K., Rendell, A. P., Burant, J. C., Iyengar, S. S., Tomasi, J., Cossi, M., Millam, J. M., Klene, M., Adamo, C., Cammi, R., Ochterski, J. W., Martin, R. L., Morokuma, K., Farkas, O., Foresman, J. B., Fox, D. J. Gaussian, Inc., Wallingford CT, 2016.
6. Yamakado, T., Otsubo, K., Osuka, A. & Saito, S. Compression of a Flapping Mechanophore Accompanied by Thermal Void Collapse in a Crystalline Phase. *J. Am. Chem. Soc.* **140**, 6245–6248 (2018).
7. Varol, H. S. et al. Nanoparticle amount, and not size, determines chain alignment and nonlinear hardening in polymer nanocomposites. *Proc. Natl. Acad. Sci. USA* E3170–E3177 (2017).
8. Yuan, C., Saito, S., Camacho, C., Kowalczyk, T., Irle, S. & Yamaguchi, S. Hybridization of a Flexible Cyclooctatetraene Core and Rigid Aceneimide Wings for Multiluminescent Flapping π Systems. *Chem. Eur. J.* **20**, 2193–2200 (2014).
