# Supplementary material for: Bridging pico-to-nanonewtons with a ratiometric force probe for monitoring nanoscale polymer physics before damage
Source: Nat Commun. 2022 Jan 13;13:303. doi: 10.1038/s41467-022-27972-y (PMC8758707; doi:10.1038/s41467-022-27972-y)
Supplement: Supplementary file 3 — Description of Additional Supplementary Files [file 41467_2022_27972_MOESM3_ESM.pdf]

Description of Additional Supplementary Files

File Name: Supplementary Movie 1

Description: Monitoring force transmission of the stretched polycarbonate film chemically doped with the dual fluorescent molecular force probe. Rapid growth of the stressed area was visualized much earlier than crack propagation, demonstrating a lower threshold for the fluorescence switch of the force probe, well below the force required for covalent bond scission. Excitation wavelength: 365 nm. The field of view of the microscope: 1 mm diameter. Stretching rate: 5 µm s–1 . Strain rate: 2 ´ 10–4 s–1 .
